# Metataxonomic strategy to decipher gut bacteriome biomarkers of type 2 diabetes and obesity in Tunisian population

**DOI:** 10.1042/BSR20260157

**Published:** 2026-10-13

**Authors:** Meriem Fassatoui, Thierry Pedron, Nadia Kheriji, Meriem Hechmi, Hamza Dallali, Haifa Jmel, Henda Jamoussi, Abdelmajid Abid, Philippe J. Sansonetti, Rym Kefi

**Affiliations:** 1 Laboratory Transmission, Control and Immunobiology of Infections, Pasteur Institute of Tunis, Tunis, Tunisia; 2 University of Tunis El Manar, Campus universitaire Farhat Hached, Tunis, Tunisia; 3 Unité de Pathogénie Microbienne Moléculaire, INSERM U1202, Institut Pasteur, Paris, France; 4 Laboratory of Biomedical Genomics and Oncogenetics, Institut Pasteur de Tunis, Tunis, Tunisia; 5 National Institute of Applied Science and Technology, University of Carthage, Tunis, Tunisia; 6 Research unit on obesity UR18ES01, Faculty of medicine, University Tunis El Manar, Tunis, Tunisia; 7 Chaire de Microbiologie et Maladies Infectieuses, Collège de France, Paris, France

**Keywords:** Bacteriome, Diabetes, Gut, Metataxonomic, Obesity, Tunisia

## Abstract

Metabolic disorders are multifactorial diseases and major global health concerns, with many individuals worldwide responding inadequately to treatments. The gut bacteriome regulates metabolism and offers therapeutic potential; however, research from North Africa remains scarce despite the growing prevalence of non-communicable diseases in the region. This study examines gut bacterial profiles of Tunisian type 2 diabetic (T2D), obese (OB), and non-diabetic (ND) individuals within the framework of personalized medicine. Ninety-five participants were enrolled [33 ND, 26 T2D, 26 T2D with obesity and cardiovascular complications (T2D_OBCV) and 10 OB]. Bacterial 16S rDNA V3–V4 regions were sequenced on Illumina MiSeq and analyzed using the QIIME 2 pipeline. Obese subjects exhibited gut dysbiosis with lower *Akkermansia* (*P*=0.02) and *Ligilactobacillus* (*P*=0.04) levels, an enrichment of *Bifidobacterium* (*P*=0.004), as well as *Sutterella* (*P*=0.03) and *Erysipelatoclostridium* (*P*=0.02). However, the gut bacteriome landscape of T2D patients reveals differences in the distribution of *Shigella* (*P*=0.01) highly abundant in the intestine, and a depletion of *Clostridia* vadin BB 60 group (*P*=0.02) and *Oscillospiraceae* UCG 005 (*P*=0.01). Noting that the gut bacteriome of T2D_OBCV patients is characterized by a depletion of *Asteroleplasma* (*P*=0.01), *Oscillospiraceae* UCG 005 (*P*=0.02), *Eubacterium ruminantium* group (*P*=0.0007) and *Romboutsia* (*P*=0.002) beside an enrichment of *Fusicatenibacter* (*P*=0.02). Gut bacteriome alterations in Tunisian T2D and obese subjects may serve as metabolic biomarkers.

## Introduction

Diabetes mellitus is a serious worldwide health problem affecting 537 million adults worldwide in 2021 according to the International Diabetes Federation [1]. The prevalence of this disease is predicted to reach over 783 million cases by 2045 [1]. Hence, the interest in investigating mechanisms and pathways implicated on the onset of the ailment to prevent the disease and to improve diabetes healthcare and therapy [2]. This disease is associated with insulin resistance as well as an altered production of insulin [2]. The burden of this metabolic disorder is linked to its subsequent complications namely nephropathy, hypertension, blindness, limb amputations and susceptibility to infections [2].

Metabolic disorders are recognized as a worldwide health concern [3]. The tragic increase of the prevalence of those diseases is attributed to multiple factors [3]. Gut microbiota plays a key role in Host physiological regulation [3]. This organ has been investigated for therapeutic purposes [2].

The Human gastrointestinal tract harbors roughly 100 trillion of microbes encompassing archaea, bacteria, fungi, bacteriophages and eukaryotic viruses [4] mostly represented by bacteria [5]. The composition of gut bacteria is dominated by nine major phyla: Firmicutes, Bacteroidetes, Actinobacteria, Fusobacteria, Proteobacteria, Verrucomicrobia, Cyanobacteria, Spirochaetes and VadinBE97 [6]. Gut microbiota composition is influenced by genetic determinants as well as environmental factors (anthropometric parameters, diet and drugs) [5]. The gut microbiota regulates metabolism and plays a key role in Human health homeostasis, host phenotype and fitness due to the rich genomic background held by this community (3 million of genes) compared with the Human genome (23,000 genes) [5]. Indeed, the gut microbiota ensures the fermentation of dietary fibers and produces gases and metabolites, e.g. short chain fatty acids (SCFAs) impacting energy homeostasis and interfering with the maturation and regulation of immune responses and host defense [7,8]. An altered gut microbiota composition promotes the onset of type 2 diabetes (T2D) mellitus and its complications (e.g., kidney failure, hypertension, diabetic retinopathy and atherosclerosis) [9]. Dysbiosis of gut microbiota is promoted by Westernization of lifestyle and high caloric uptake. The etiopathology of metabolic disorders has been attributed to gut bacterial metabolites namely short chain fatty acids, bile acids, branched chain amino acids, tryptophan and indole [3]. Trimethylamine N oxide (TMAO) is also a metabolite produced by gut microbiota with harmful effect on health and which could be associated with cardiovascular diseases [8].

Altered metabolic pathways (lipolysis, aminogenesis, fatty acid, oxidation and ketogenesis) and a dysregulation of phospholipids, triglycerides and TMAO have been reported in subjects with metabolic disorders [3] and could be promoted by certain gut bacterial taxa [10]. It was reported that *Faecalibacterium, Bifidobacterium, Bacteroides* and *Akkermansia* were negatively linked to T2D [11]. Moreover, *Bifidobacterium, Faecalibacterium* and *Akkermansia* could prevent the onset of T2D [11]. *Bacteroides fragili*s and *Akkermansia muciniphila* stimulate the secretion of anti-inflammatory cytokines and chemokines [interleukin 10 (IL10)] protecting against inflammatory reactions and improving insulin sensitivity [11].

In the present study, we deciphered the composition of gut bacteriome and its implication in the pathogenesis of obesity, T2D and its complications in a Tunisian cohort compared with non diabetic subjects using metataxonomic approach.

## Materials and methods

### Description of the study subjects

We enrolled 95 Tunisian participants from the Institut Pasteur de Tunis and the National Institute of Nutrition of Tunis. Patients and non diabetic individuals were recruited among men and women between the ages of 30 and 77 years. Diabetes was screened referring to the standards of medical care in diabetes [12]: Fasting plasma glucose ≥7 millimoles per litre (mmol/l), 2-h plasma glucose ≥11.1 mmol/l, glycated hemoglobin (HbA1c) ≥6.5%. Obesity is defined as an excess of fat mass and was diagnosed according to the World Health Organization (WHO) criteria: [Body Mass Index (BMI) ≥30 kg/m^2^] [13].

Non diabetic subjects have normal fasting plasma glucose and glycated hemoglobin levels.

Patients diagnosed with HIV, cancer, autoimmune diseases or cognitive impairment, as well as those who were pregnant, using immunosuppressants, under antibiotic treatment for at least two months prior to and during stool sampling or had taken probiotics within the past three months, were excluded from this study. Besides, all participants receiving herbal treatments were excluded from this study.

Additionally, participants had been consulting nutritionists for dietary advice.

During enrollment, we evaluated potential confounding factors, including anthropometric parameters (e.g., sex and age) and medication use. Participants exhibiting factors likely to influence gut bacteriome composition (e.g., unhealthy dietary habits) were excluded from the study.

Following clinical and biochemical assessments, the 95 participants were categorized as follows: 26 patients with T2D, 33 ND individuals, 26 T2D_OBCV patients and 10 OB subjects. Participant characteristics are presented in Supplementary Table S1.

Some participants with T2D were treated with metformin, while others received insulin, or a combination of metformin and insulin.

Stool samples from the 95 participants were collected in sterile containers and stored at −80°C until subsequent analysis.

### Microbial DNA extraction

Microbial DNA was extracted from 250 mg of fecal samples using MoBioPower fecal DNA isolation kit (12830, Qiagen) according to the manufacturer’s recommendations. We evaluated the DNA concentrations and purity by means of NanoDrop ND-2000 Spectrophotometer (ND-2000, Thermo Scientific). DNA was stored at −20°C until further processing.

### Library preparation of bacterial 16S rDNA and sequencing

We extracted microbial DNA from fecal samples at a concentration of 5 ng/μl and amplified the 16S rDNA V3–V4 hypervariable regions by polymerase chain reaction (PCR) using the KAPA HiFi HotStart ReadyMix kit (7958935001, Roche Diagnostics) with forward 5′-TCGTCGGCAGCGTCAGATGTGTATAAGAGACAGCCTACGGGNGGCWGCAG-3′ and reverse 5′-GTCTCGTGGGCTCGGAGATGTGTATAAGAGACAGGACTACHVGGGTATCTAATCC-3′ primers including Illumina Overhang adapter sequences. PCR reaction was achieved under the following conditions: 95°C for 3 min, 25 cycles of 95°C for 30 s, 55°C for 30 s, 72°C for 30 s, with a final extension at 72°C for 5 min on a thermal cycler (GeneAmp PCR system 9700, Applied Biosystems). Amplicons were purified using AMPure XP beads (A63881, Beckman Coulter). Illumina adapters sequences and barcodes were attached to sequences by a second PCR using Nextera XT index kit (FC-131-1002, Illumina). The following program was run for amplification: 95°C for 3 min 8 cycles of: 95°C for 30 s, 55°C for 30 s, 72°C for 30 s and final extension 72°C for 5 min. The products were pooled in equimolar concentration and diluted in 10 mM Tris–HCl pH 8.5 to 2 nM. A library quality control check was performed using Fragment Analyzer device (FSv2-CE2, Proteigene). The denaturation of the sequencing library was achieved with 0.2 N NaOH diluted in hybridization buffer containing 10% PhiX, supplied with the PhiX Control Kit v3 (FC-110-3001, Illumina). Paired end (2 × 300 base pairs (bp)) sequencing was performed on Illumina Miseq instrument (Miseq^™^ 2013) using MiSeq reagent kit v3 (MS-102-3003, Illumina).

### Bioinformatic analyses

The sequences were demultiplexed according to their barcodes, each pair were merged by FLASH version 1.2.11 [14]. Primers and adapters sequences were retrieved by cutadapt 2.6 [15]. DNA fragments containing ambiguous bases N and/or sequences were removed using PRINSEQ-lite 0.20.3 tool [16].

We performed quality control check of DNA sequences using QIIME 2, we trimmed low quality sequences (phred score <20) and we clustered features into OTUs referring to the identity threshold of (85%) via VSEARCH method included in the latest QIIME2 version for amplicon analysis [17].

Taxonomic assignment was performed referring to SILVA database including curated ribosomal RNA gene sequences of bacteria and archaea [18] using QIIME 2 script.

Alpha diversity was assessed using the Shannon index, while beta diversity was evaluated based on the Bray–Curtis metric. Principal Coordinate Analysis (PCoA) plots and heatmaps were generated using QIIME 2.

### Statistical methods

Alpha diversity analysis was performed to assess bacterial richness within samples and evaluate changes in the gut microbiota of T2D and obese patients compared with the ND group. The Kruskal–Wallis test was used to compare alpha diversity, representing the total bacterial richness within the intestinal ecosystem. Beta diversity, reflecting bacterial variation between samples, was assessed using the PERMANOVA method.

We used Analysis of Compositions of Microbiomes with Bias Correction (ANCOM-BC) in QIIME 2 to identify differentially abundant bacteria between groups. Additionally, the ADONIS test in QIIME 2 was applied to evaluate the influence of potential confounding variables, including sex, age, and medication.

### Assessment of SCFAs production

Picrust2 [19] was used to predict the metabolic potential of the bacteriome, with a particular focus on pathways involved in the production of the major SCFAs, including acetate, butyrate, and propionate, as well as other fermentation metabolites such as lactate, formate, and succinate. Predicted Enzyme Commission (EC) numbers generated by PICRUSt2 were used to assess the functional potential of the bacteriome. QIIME 2 was subsequently used to generate bar plots and perform differential abundance analysis using the ANCOM-BC method.

## Results

### Gut bacterial profiles of type 2 diabetic, obese, T2D_OBCV and non diabetic participants

We investigated the gut bacterial profile of the cohort subdivided in four groups (ND, T2D, Obese and T2D_OBCV) aiming to decipher changes in the gut bacterial composition between groups. While comparing the profile of intestinal bacteriome between the four groups T2D, T2D_OBCV, obese and ND subjects, we detected a shift in the distribution of *Escherichia Shigella* in T2D patients, as well as in *Bifidobacterium* and *Akkermansia* in obese subjects (Figure 1).

**Figure 1:**
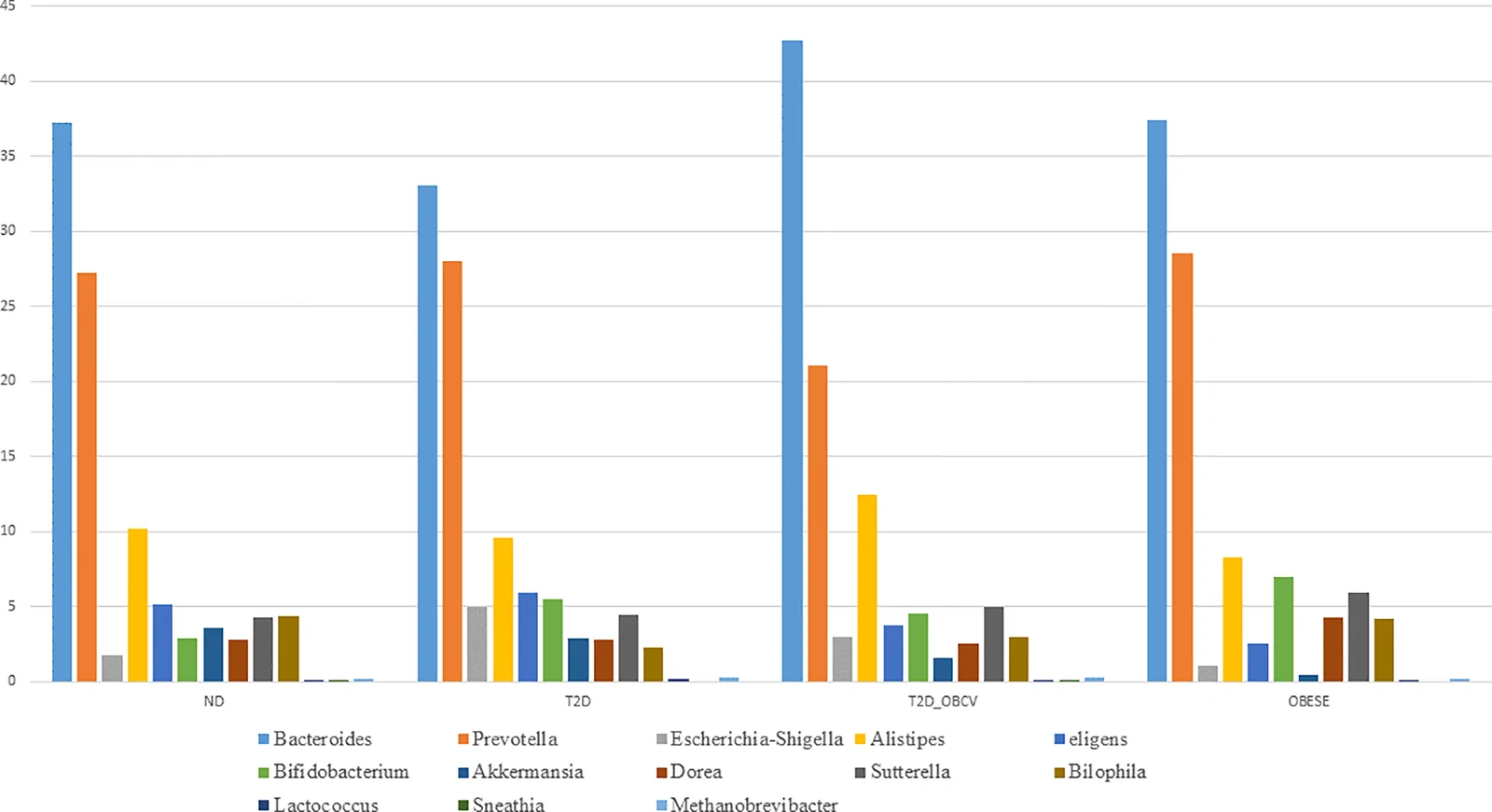
Gut bacterial profile of obese and type 2 diabetic patients compared with controls ND: non diabetic subjects, OB_T2D_OBCV: Obese and type 2 diabetic patients with obesity and cardiovascular complications.

### Investigation of gut bacterial diversity of type 2 diabetic, obese, type 2 diabetic with obesity and cardiovascular complications and non diabetic individuals

We estimated the alpha diversity and changes in bacterial taxa between the four groups: T2D, OB, T2D_OBCV and ND subjects. The alpha diversity is approximately similar in all the prospected cohort (*P*=0.61).

The evaluation of the beta diversity of intestinal bacteria in T2D, T2D_OBCV, obese and ND groups indicates a comparable distribution of gut bacteria (Figure 2, *P*=0.74).

**Figure 2:**
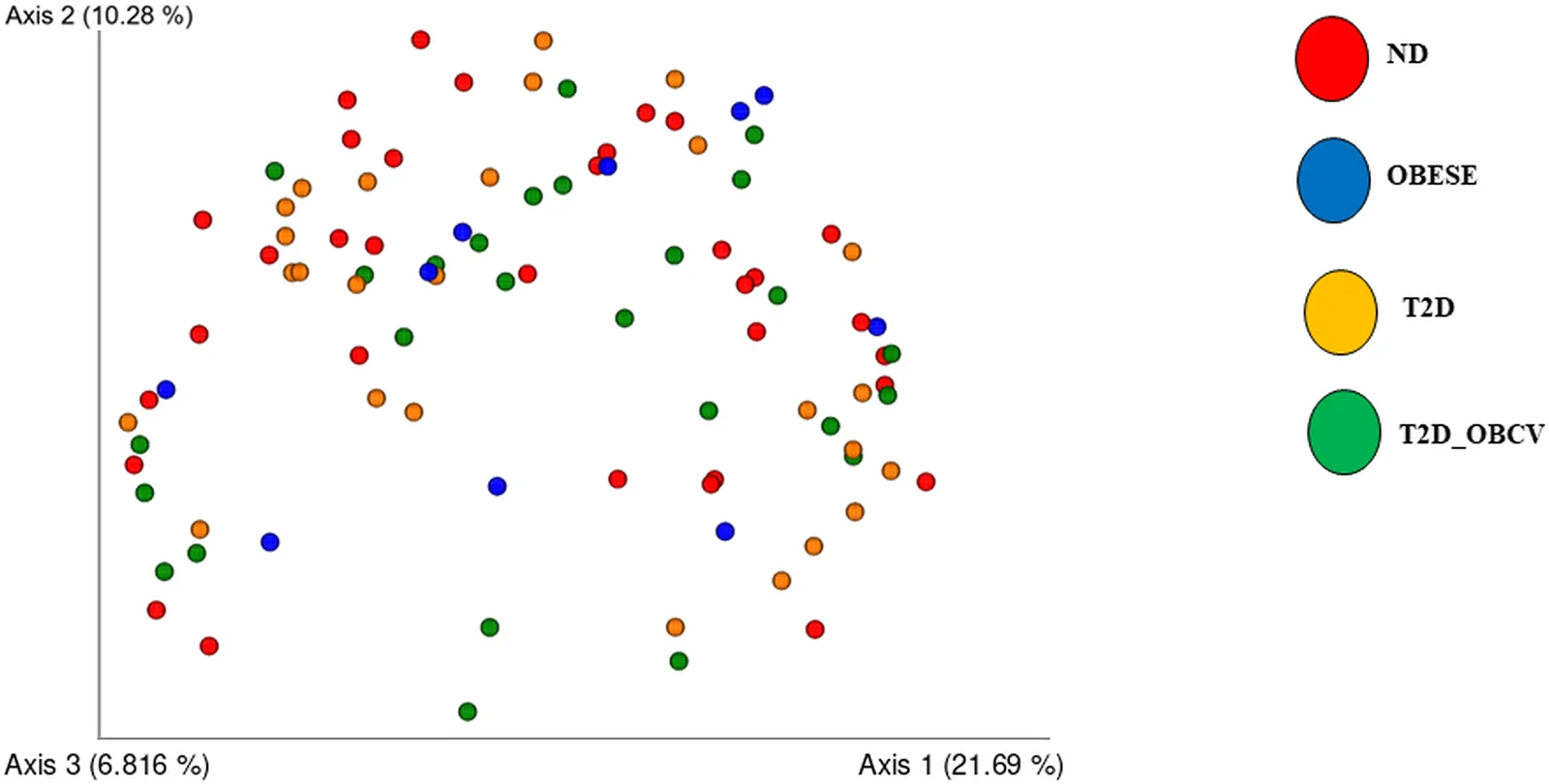
Principal coordinate analysis of gut bacteria composition in the study cohort ND non diabetic group, T2D type 2 diabetic patients, T2D_OBCV type 2 diabetic patients with obesity and cardiovascular complications.

### Estimation of the abundance of gut bacterial species in the investigated cohort

At the species level, we determined first the percent distribution of gut bacteria across the two groups OB_T2D_OBCV and ND participants aiming to detect potential gut bacteriome changes. Obese subjects show an imbalanced gut bacteriome marked by a decline in the proportions of *Akkermansia* (*P*=0.02) and *Ligilactobacillus* (*P*=0.04), an enrichment of *Bifidobacterium* (*P*=0.004), as well as *Sutterella* (*P*=0.03) and *Erysipelatoclostridium* (*P*=0.02) (Figure 3). Changes in the gut bacteriome composition of T2D patients were revealed, characterized by an overgrowth of *Shigella* (*P*=0.01) and a depletion of *Clostridia* vadin BB 60 group (*P*=0.02) and *Oscillospiraceae* UCG 005 (*P*=0.01) (Figure 4).

**Figure 3:**
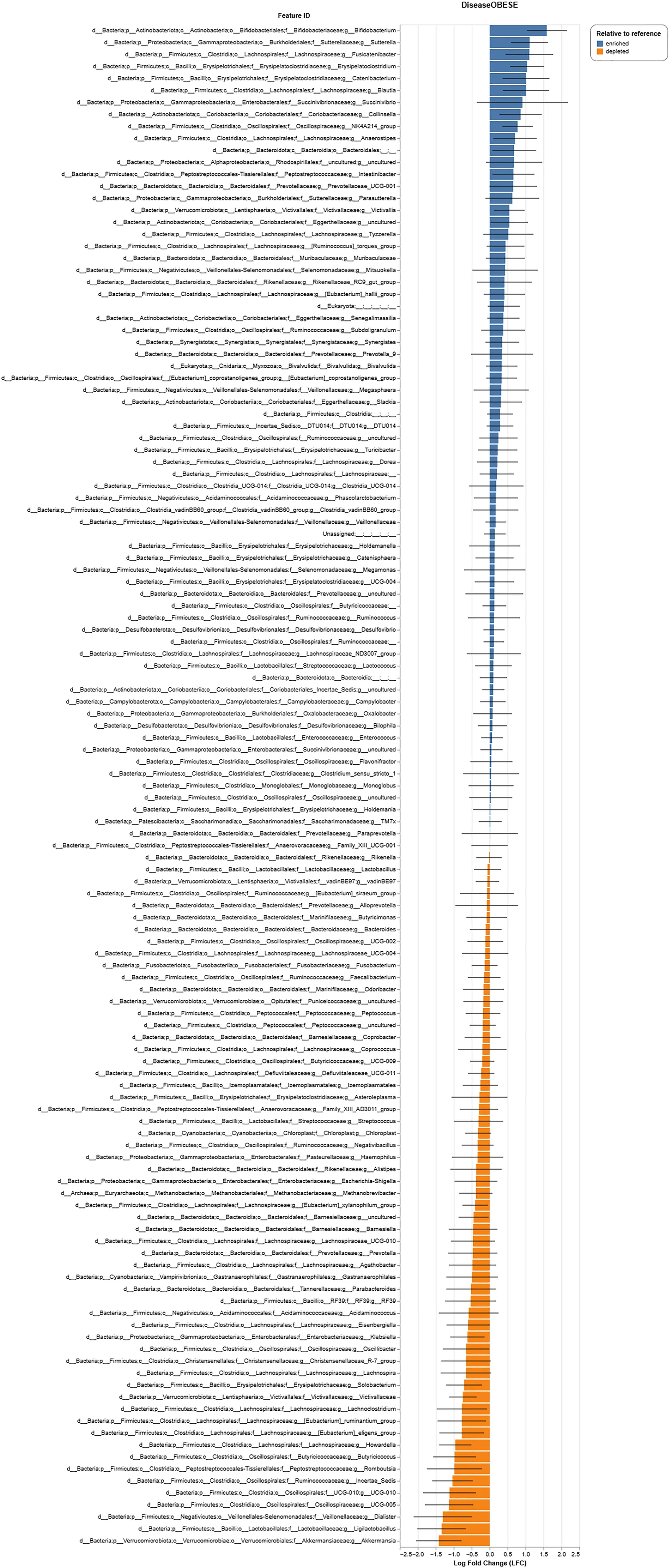
Distribution of gut bacteria across obese group

**Figure 4:**
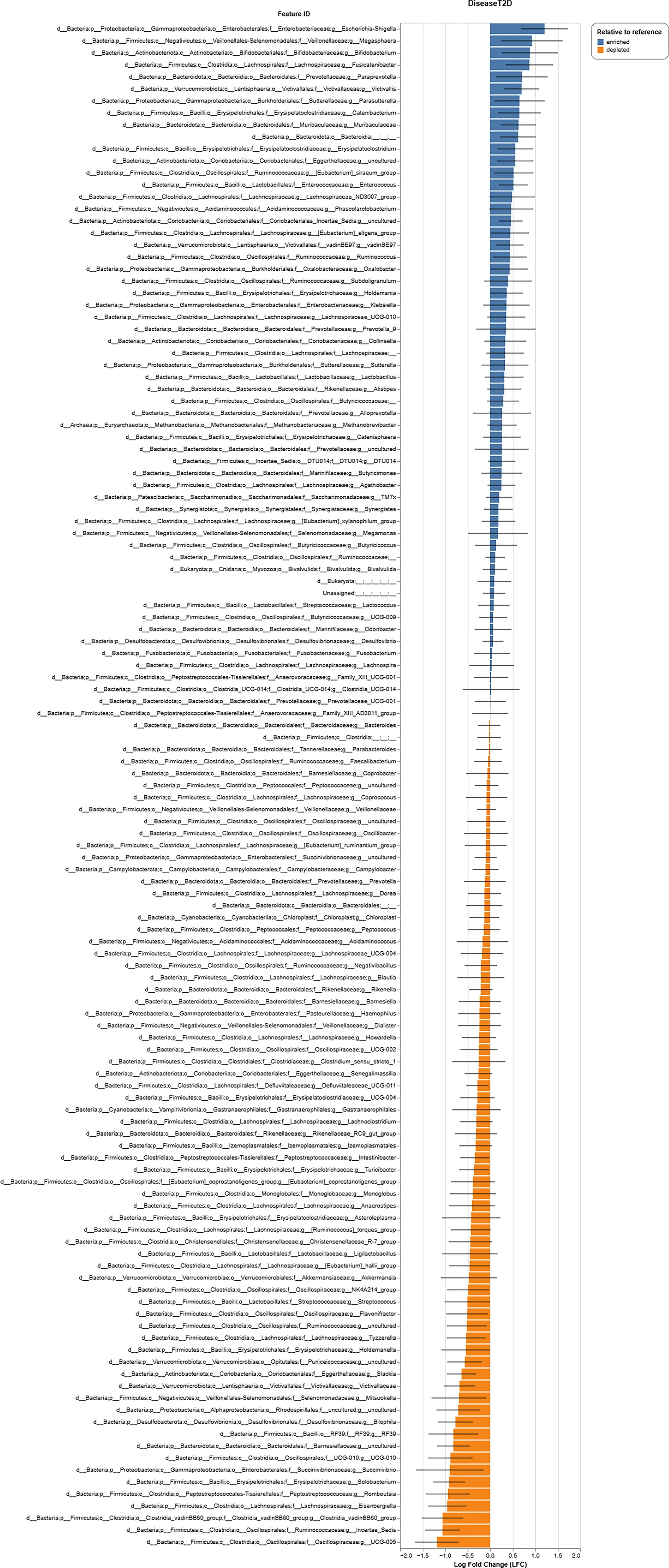
Relative abundance of gut bacteria in T2D patients

Gut bacteriome ecosystem of T2D_OBCV patients is depleted from *Asteroleplasma* (*P*=0.01), *Oscillospiraceae* UCG 005 (*P*=0.02), *Eubacterium ruminantium* group (*P*=0.0007) and *Romboutsia* (*P*=0.002) beside an enrichment of *Fusicatenibacter* (*P*=0.02) (Figure 5).

**Figure 5:**
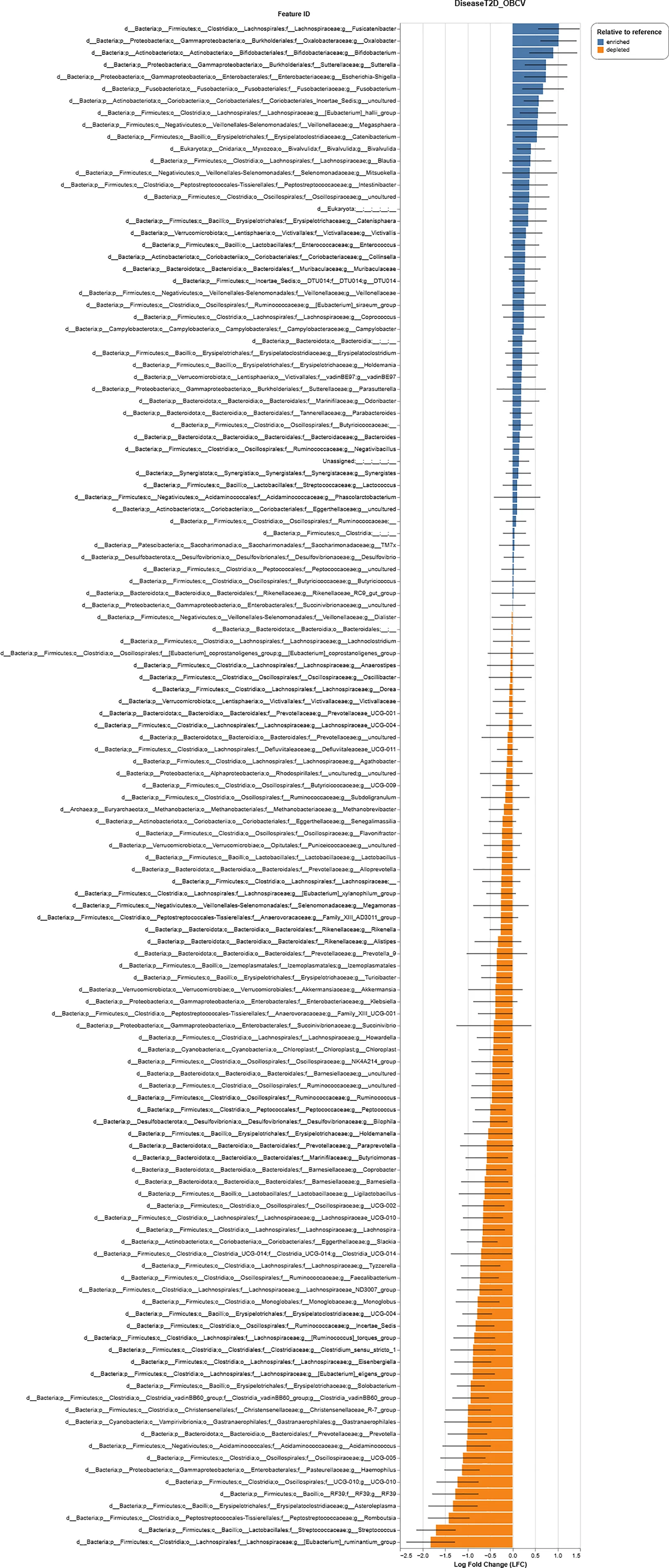
Gut bacteriome landscape of T2D_OBCV patients

Moreover, Heatmap shows that each individual holds a unique gut bacteriome signature (Figure 6). We also categorized the samples into four groups (ND, T2D, Obese, and T2D_OB) for the heatmap analysis and reconstructed the heatmaps accordingly (Supplementary Figure S1). As shown, each group exhibits a distinct gut bacteriome profile while also sharing a common core bacteriome signature.

**Figure 6:**
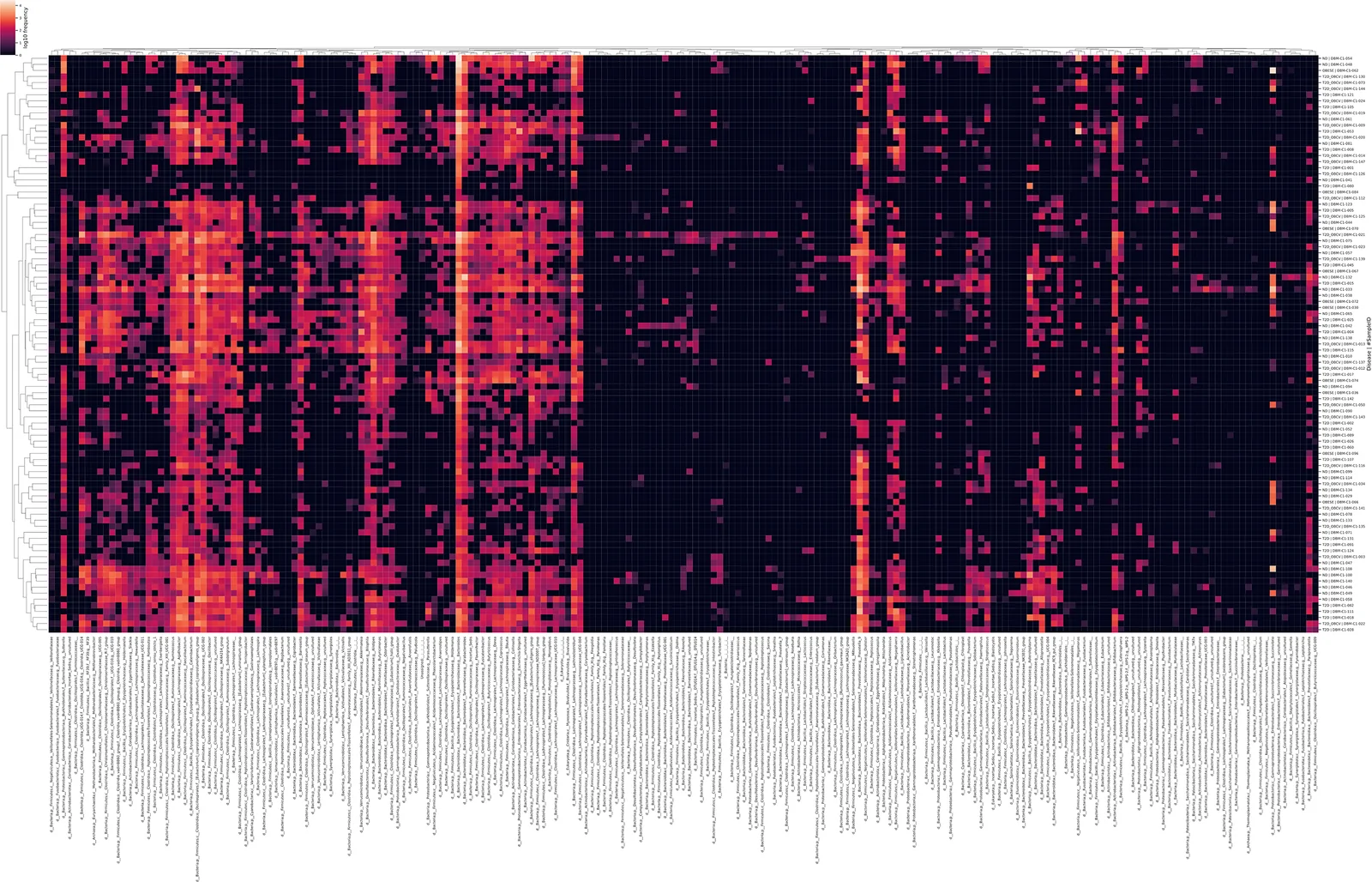
Heatmap revealing the gut bacteriome profile of each individual Orange color indicates that the bacterium genus is highly abundant while black color signifies that the gut bacteria is depleted or low abundant. ND non diabetic group, T2D type 2 diabetic patients, T2D_OBCV type 2 diabetic patients with obesity and cardiovascular complications.

### Impact of confounded variables on gut microbiota composition

We assessed potential confounding factors sex, age and medication use that may affect gut bacteriome composition. According to the result provided by ADONIS test, the above mentioned factors would not affect our study of gut bacteriome [sex (*P-*value = 0.461), age (*P-*value = 0.754)], drug [oral antidiabetic agent (*P-*value = 0.114), and insulin treatment (*P-*value = 0.962)]. We have included the ADONIS test results in the Supplementary tables file (supplementary Table S2).

### Profiling of short-chain fatty acids produced by key gut bacteriome in the study cohort

Relative to the ND group, the results indicate a higher predicted potential for acetate production in obese participants (Supplementary Figure S2), which correlated with an increased abundance of *Bifidobacterium*. Compared with the ND group, participants with T2D (Supplementary Figure S3) and T2D_OBCV (Supplementary Figure S4) exhibited an enrichment of propionate-producing pathways. Across all participant groups, the predicted capacity for butyrate production was reduced, consistent with the depletion of known butyrate-associated taxa, including *Clostridia vadin BB60 group, Oscillospiraceae UCG-005* (*P*=0.01), and *Eubacterium ruminantium*.

## Discussion

This is the first study reporting the gut bacteriome composition in Tunisian T2D, OB and T2D_OBCV patients. Phylum-level analysis across all subjects showed a higher abundance of Firmicutes and Bacteroidetes, consistent with previous studies [20]. We also detected bacterial sequences classified as unassigned or unclassified, potentially representing novel taxa. Additionally, obese subjects exhibited a significant reduction in Verrucomicrobia compared with T2D, T2D_OBCV and ND individuals, consistent with previous reports of decreased Verrucomicrobia in obesity [21].

At the species level, we identified a dysbiotic gut bacterial profile of T2D and obese Tunisian subjects mainly distinguished respectively by a decrease in the amounts of certain bacterial species namely *Clostridia* vadin BB 60 group and *Akkermansia muciniphila* and an increase in the proportions of Enterobacteriaceae (*Shigella*) as well as *Bifidobacterium*. These findings align with a recent study in Romanian T2D patients, which reported a decline in butyrate-producing bacteria alongside an increase in pathogenic bacteria such as *Enterobacteriaceae* [2].

*Clostridia* has been reported to be associated with Bacteroidia in the production of DNA topoisomerase, oxaloacetate decarboxylase and 6-beta-glucosidase, whereas in conjunction with Gamma-proteobacteria, it contributes to the production of 6-phospho-beta-glucosidase and protein N-phosphohistidine sugar phosphotransferase [22] This highlights the role of *Clostridia* in metabolic regulation, and its depletion in Tunisian T2D patients may disrupt key metabolic pathways.

Our findings also revealed elevated levels of Enterobacteriaceae in Tunisian patients with T2D. Previous studies have reported that hyperglycemia facilitates the invasion of pathogens belonging to the Enterobacteriaceae family [23]. According to a joint Chinese–Scandinavian study, Danish and Swedish patients with T2D exhibited a high abundance of Enterobacteriaceae in their gut microbiota, whereas this pattern was not observed in Chinese T2D patients [2]. Indeed, the abundance of Enterobacteriaceae may be influenced by ethnic or demographic factors [2]. High blood glucose levels may promote the invasion of pathogens like *Shigella* in Tunisian T2D patients.

Obesity is a metabolic disorder that may lead to serious complications namely diabetes, cardiovascular disease and hypertension [24]. This disease has been linked to gut microbiota dysbiosis characterized by impaired gut barrier function, increased inflammation, and enhanced energy storage [25].

We examined the gut bacteriome of obese Tunisian individuals and found an imbalance marked by reduced *Verrucomicrobia*. At the species level, *Bifidobacterium* was enriched, while *Akkermansia muciniphila* was depleted.

High body weight may be associated with elevated levels of *Bifidobacterium*, as weight loss has been correlated with a reduction in the proportion of this bacterium [26]. This finding contrasts with a study on gut microbiota composition in mice, which reported that *Bifidobacterium* supplementation in high-fat diet rats exerted beneficial effects by reducing mesenteric white fat accumulation and improving hepatic insulin sensitivity [27]. *Bifidobacterium* has also been shown to stimulate the synthesis and secretion of glucagon-like peptide-1 (GLP-1), thereby enhancing insulin response and improving glycemic regulation [27]. This observation may partly explain why obese Tunisian individuals with elevated levels of *Bifidobacterium* appeared to be protected against diabetes.

A decrease in *Bifidobacterium* abundance was likewise observed in Chinese subjects with visceral obesity [24]. Noting that *Bifidobacterium* regulates energy metabolism, immunity and strength intestinal health [28]. The gut microbiota of obese Tunisian patients exhibits an increased abundance of *Bifidobacterium*. This observation may reflect a gradual modulation of their gut microbiota profile, potentially resulting from dietary guidance received during consultations with nutritionists.

We observed lower levels of *Akkermansia* in obese Tunisian individuals, potentially affecting energy balance. This mucin-degrading bacterium is considered an indicator of a healthy metabolic status [2].

*Akkermansia* ensures the fermentation of mucin and produces oligosaccharides, propionate triggering fatty acid receptors, G protein-coupled receptors GPR41 and GPR43 implicated in the activation of intestinal L cells and the secretion of the incretin hormones GLP 1, GLP2 as well as peptide YY (PYY) [29]. PYY promotes satiety, GLP1 enhances insulin secretion, satiety and retard gastric emptying [29]. Moreover, GPR43 activates anti-lipolytic process in white adipose tissue improving therefore metabolic regulation, leading to a decrease of fat mass establishment as well as dyslipidemia and ameliorating body weight control [29]. This bacteria is also implicated in the consolidation of the integrity of the epithelial gut barrier, triggering therefore the intestinal immunity and preventing leaky gut and inflammation [30]. It was reported that obese subjects show also high bile acids serum levels [3]. Bile acids control triglycerides pathways via fibroblast growth factor 19 (FGF19) and FGF15. The regulation of insulin sensitivity in the liver, insulin secretion in the pancreas energy expenditure, glycogen production and thermogenesis is promoted by Farnesoid X Receptor (FXR) and Takeda G Protein Receptor 5 (TGR5). Altered gut microbiota composition affects bile acids metabolism and favors accumulation of primary bile acid chenodeoxycholic acid (CDCA) promoting intestinal permeability [3]. An enrichment with *Akkermansia* could protect against obesity [31]. *Akkermansia* has been identified as a promising candidate for next-generation probiotics. Probiotic administration can strengthen intestinal barrier function, enhance gut peptide secretion and reduce chronic low-grade inflammation, thereby supporting therapeutic strategies against metabolic dysfunction [32].

Obese Tunisian subjects showed lower *Ligilactobacillus* abundance, a bacterium known for its antimicrobial and antioxidant effects and its role in promoting metabolic health [33]. In the obese cohort, the loss of this strain may compromise normal metabolic processes.

Obese Tunisian individuals showed elevated levels of *Sutterella* and *Erysipelatoclostridium*. Enrichment of *Sutterella* has been connected to specific human health concerns, potentially promoting pro-inflammatory responses [34]. Studies have indicated that a depletion of *Erysipelatoclostridium* promotes the accumulation of sugar acids and sugar alcohols [35]. This supports the role of this bacterium in metabolic regulation. Indeed, its enrichment in obese Tunisian subjects appears to have beneficial effects on metabolic control, potentially helping to prevent the onset of diabetes.

A dysbiosis of the gut bacteriome was observed in T2D_OBCV Tunisian patients, characterized by a depletion of *Asteroleplasma, Oscillospiraceae UCG-005, Eubacterium ruminantium* group and *Romboutsia*, along with an enrichment of *Fusicatenibacter*. Notably, depletion of *Asteroleplasma* has also been reported in patients with ulcerative colitis, suggesting that loss of this bacterium may be linked to disease onset [36].

*Oscillospiraceae UCG-005* has been reported to possess anti-inflammatory properties and to produce SCFAs through the fermentation of dietary fibers, thereby supporting gut homeostasis [37]. The loss of this bacterium in the gut of T2D_OBCV Tunisian patients may contribute to disease progression, potentially linked to heightened inflammatory processes.

*Eubacterium ruminantium*, found in the rumen of cattle and goats, catabolizes carbohydrates and produces unsaturated fatty acids, such as palmitoleic acid [38] Consumption of meat leads to the accumulation of L-carnitine, which is metabolized by gut microbiota into trimethylamine (TMA) and subsequently converted to TMAO via γ-butyrobetaine (γBB), a process associated with an increased risk of cardiovascular diseases [39]. The depletion of this strain may result from the dietary regimen followed by T2D_OBCV Tunisian patients to prevent further deterioration of their health.

*Romboutsia* has been reported to be more abundant in healthy and prediabetic individuals compared to those with T2D. As a butyrate-producing bacterium, it is considered a potential candidate for the treatment of metabolic disorders [40]. The loss of this bacterium in T2D_OBCV Tunisian patients may contribute to metabolic dysfunction.

*Fusicatenibacter* activates anti-inflammatory pathways by inducing IL-10 in the intestinal mucosa and also promotes SCFA production, thereby strengthening mucosal integrity [41]. The enrichment of this bacterium in the gut microbiota of T2D_OBCV Tunisian patients may reflect the healthy dietary habits adopted by this group.

These results are consistent with previous gut microbiome studies in Indian patients with T2D, suggesting that the *Clostridia vadinBB60* group could serve as a potential biomarker for this population [42].

The altered gut bacteriome observed in the Indian T2D cohort was characterized by shifts in the abundance of *Lactobacillus* spp. and *Ruminococcaceae* spp., mirroring the microbial alterations previously identified in Tunisian individuals with T2D [43].

A study of Romanian patients with T2D identified *Sutterella* as being associated with disease onset and complications, a finding that is consistent with the enrichment of this genus observed in our Tunisian T2D cohort. Furthermore, *Sutterella* is known to produce succinate, an organic acid generated in response to metabolic stress. Elevated succinate levels may facilitate pathogen invasion by serving as a nutrient source, thereby potentially contributing to disease progression and gut microbial dysbiosis [44].

An enrichment of *Asteroleplasma* has been reported in Indian individuals with T2D; however, in our Tunisian cohort, this genus was found to be depleted. This discrepancy may reflect population-specific factors, particularly differences in dietary habits that are unique to the Indian population [42].

The normal glucose tolerance Indian cohort was characterized by a predominance of *Akkermansia* and *Ligilactobacillus* in the gut microbiota, in contrast to the obese Tunisian group, which exhibited a depletion of these taxa. These bacteria are known to exert beneficial effects on host health by producing anti-inflammatory metabolites and reinforcing gut barrier integrity, thereby contributing to the regulation of inflammatory responses [42].

As shown by the heatmap (Figure 6), each study participant exhibited a distinct gut bacteriome signature. This finding supports the concept of personalized medicine, where treatments could be tailored according to an individual’s gut microbiota landscape.

To investigate the enrichment and depletion of specific bacterial taxa and their potential implications for the production of gut microbiota-derived SCFAs, including acetate, propionate, and butyrate, we compared our findings with previous reports in the literature.

The gut bacteriome of Tunisian obese subjects is characterized by a depletion of *Akkermansia* and *Ligilactobacillus*. *Akkermansia* has been reported to produce acetate and to indirectly promote propionate production through microbial cross-feeding mechanisms [45,46], whereas *Ligilactobacillus* primarily produces lactate, with only limited acetate production [47]. These observations are in agreement with our predicted functional profiling of the gut bacteriome, which revealed a reduced capacity for propionate production in Tunisian obese subjects, consistent with the depletion of *Akkermansia*. Similarly, the depletion of *Ligilactobacillus* was accompanied by a reduced predicted capacity for lactate production, supporting the potential contribution of this genus to lactate metabolism within the gut ecosystem.

In contrast, obesity in Tunisian subjects was associated with an enrichment of *Bifidobacterium, Sutterella* and *Erysipelatoclostridium*. *Bifidobacterium* produces acetate and lactate through the bifid shunt during carbohydrate fermentation [47] which is consistent with the increased predicted capacity for acetate production observed in the gut microbiome of obese Tunisian subjects. *Sutterella* has been reported to produce low levels of acetate and succinate, although its metabolic potential remains incompletely characterized [45]. Accordingly, the enhanced predicted succinate production in obese Tunisian subjects may be related, at least in part, to the increased abundance of *Sutterella*. Despite the enrichment of *Erysipelatoclostridium* in obese Tunisian subjects, our functional predictions indicated a reduced capacity for butyrate production. This finding suggests that the increased abundance of *Erysipelatoclostridium* alone is insufficient to compensate for the depletion of other butyrate-producing taxa, highlighting the complexity of microbial interactions underlying butyrate biosynthesis.

Several bacterial taxa are recognized as important butyrate producers within the gut microbiota, including *Erysipelatoclostridium*, members of the *Clostridia vadinBB60* group, *Oscillospiraceae UCG-005, Fusicatenibacter* and the *Eubacterium ruminantium* group [47,48]. In this context, the Tunisian T2D group exhibited a reduced abundance of the *Clostridia vadinBB60* group and *Oscillospiraceae UCG-005*, whereas the T2D-CV group was characterized by a depletion of the *Eubacterium ruminantium* group despite an enrichment of *Fusicatenibacter*. These compositional changes are consistent with our functional predictions, which indicated a reduced capacity for butyrate production in both groups. The enrichment of *Fusicatenibacter* alone may not be sufficient to compensate for the loss of other key butyrate-producing taxa, underscoring the collective contribution of multiple bacterial groups to butyrate biosynthesis.

In contrast, *Shigella* is not recognized as a major producer of short-chain fatty acids. Its increased abundance in the Tunisian T2D group may instead be linked to pathogenic processes [49]. Conversely, *Asteroleplasma* exhibits a fermentative metabolism associated with the production of acetate and lactate, while *Romboutsia* displays a similar fermentation profile, with an additional potential for formate production, as suggested by established fermentation pathways [45]. Notably, both *Asteroleplasma* and *Romboutsia* were depleted in the T2D-CV group. Although *Romboutsia* and *Asteroleplasma* were differentially abundant, neither genus is currently recognized as a major propionate producer. Indeed, the predicted shift in propionate production could reflect the collective metabolic potential of the bacteriome community.

SCFAs, such as acetate, propionate, and butyrate, are generated by microbial fermentation of dietary fiber and play an important role in metabolic regulation by promoting insulin secretion and improving insulin sensitivity, while also inhibiting gluconeogenesis and lipolysis [45]. Indeed, a reduction in key SCFA-producing bacterial strains can impair these metabolic processes, contributing to hyperglycemia and increased fat accumulation, whereas enrichment of these beneficial microbes enhances SCFA production and supports improved metabolic health.

In the present study, Tunisian T2D participants receiving metformin showed a limited therapeutic response. It has been reported that the efficacy of this oral antidiabetic drug is influenced by the composition of the gut microbiota [50]. Given the dysbiotic gut bacteriome of Tunisian diabetic subjects, metformin administration may be insufficient to achieve effective glycemic control. It is also recognized that metformin can modulate metabolic regulation through gastrointestinal pathways independently of the gut microbiota, influencing bile acid metabolism, intestinal hormone secretion, and suppressing hepatic gluconeogenesis [51]. Further studies on the variants influencing metformin response are needed to elucidate the reasons for its limited efficacy among Tunisian patients. Both genetic and microbial factors are likely to play a role in determining individual responses to this drug.

In summary, we observed alterations in the gut bacterial profiles of OB, T2D and T2D_OBCV Tunisian patients compared to non-diabetic individuals. These bacterial biomarkers (*Akkermansia, Ligilactobacillus, Bifidobacterium, Sutterella, Erysipelatoclostridium, Shigella, Clostridia* vadin BB 60 group, *Oscillospiraceae* UCG 005, *Asteroleplasma, Oscillospiraceae* UCG 005, *Eubacterium ruminantium* group, *Romboutsia*, and *Fusicatenibacter*) may serve as a valuable and precise tool for diagnosing metabolic disorders and could complement conventional biochemical tests, such as fasting plasma glucose and HbA1c measurements, to enable comprehensive diabetes screening.

This study is limited by the sample size. Although over 110 participants were initially recruited, some declined to provide fecal samples. Additional functional and metabolomic pathway analyses are necessary to clarify the mechanisms by which dysbiotic gut microbiota contributes to the development of metabolic disorders. We acknowledge that not performing shotgun metagenomic sequencing constitutes a limitation of this study, as it restricted our capacity to obtain high-resolution taxonomic profiling and to directly link bacteriome signatures with functional metabolic pathways.

Studies involving a larger cohort are needed to clarify how gut bacteria modulate metabolic pathways and prevent complications. Profiling the gut microbiota could also support early detection of metabolic dysfunction and enable targeted interventions through personalized medicine programs.

## Supplementary Material

Supplementary Figures S1-S4 and Tables S1-S2

## Data Availability

The data associated with this paper are publicly accessible via the following link: https://www.ncbi.nlm.nih.gov/bioproject/PRJNA1095229 and Sequence Read Archive (SRA) accession number PRJNA1095229. Institute Pasteur de Tunis is the owner of these data.

## Abbreviations

ANCOM-BC: Analysis of Compositions of Microbiomes with Bias Correction
B: Beta
BMI: body mass index
Bp: base pairs
CDCA: chenodeoxycholic acid
EC: Enzyme Commission number
FGF: fibroblast growth factor
FXR: Farnesoid X Receptor
GLP: glucagon-like peptide
GPCR: G-protein coupled receptors
HbA1c: glycated hemoglobin
HITDB: Human Intestinal Database
IFNγ: interferon γ
IL: interleukin
ND: non diabetic
NF-κB: nuclear factor κB
OB_T2D_OBCV: obese, type 2 diabetic patients and type 2 diabetic patients with obesity and cardiovascular complications
PCOa: Principal Coordinate Analysis
PCR: polymerase chain reaction
PYY: Peptide YY
SCFA: short chain fatty acid
T2D: Type 2 diabetes
T2D_OBCV: Type 2 diabetic with obesity and cardiovascular complications
TGR: Takeda G Protein Receptor
Th17: T helper 17
TMAO: Trimethyl amine N oxide
WHO: World Health Organization
