# Supplementary material for: Metataxonomic strategy to decipher gut bacteriome biomarkers of type 2 diabetes and obesity in Tunisian population
Source: Biosci Rep. 2026 Oct 13;46(10):BSR20260157. doi: 10.1042/BSR20260157 (PMC13673178; doi:10.1042/BSR20260157)
Supplement: Supplementary file 1 [file BSR-2026-0157_supp.pdf]

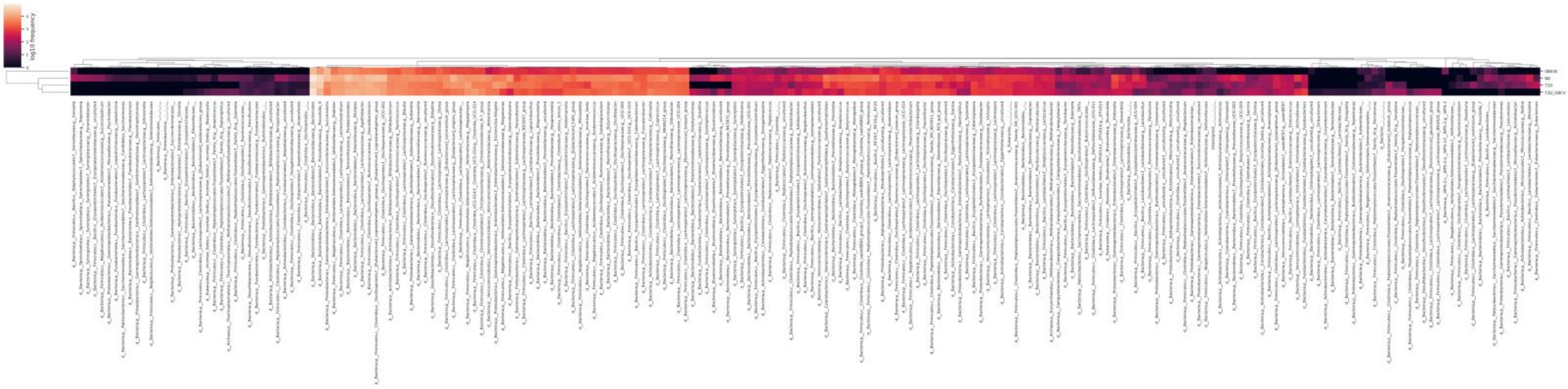

Supplementary figure 1. Heatmap representation of the gut bacteriome profile across each cohort group

Orange color indicates that the bacterium genus is highly abundant while black color signifies that the gut bacteria is depleted or low abundant

ND non diabetic group, T2D type 2 diabetic patients, T2D_OBCV type 2 diabetic patients with obesity and cardiovascular complications.

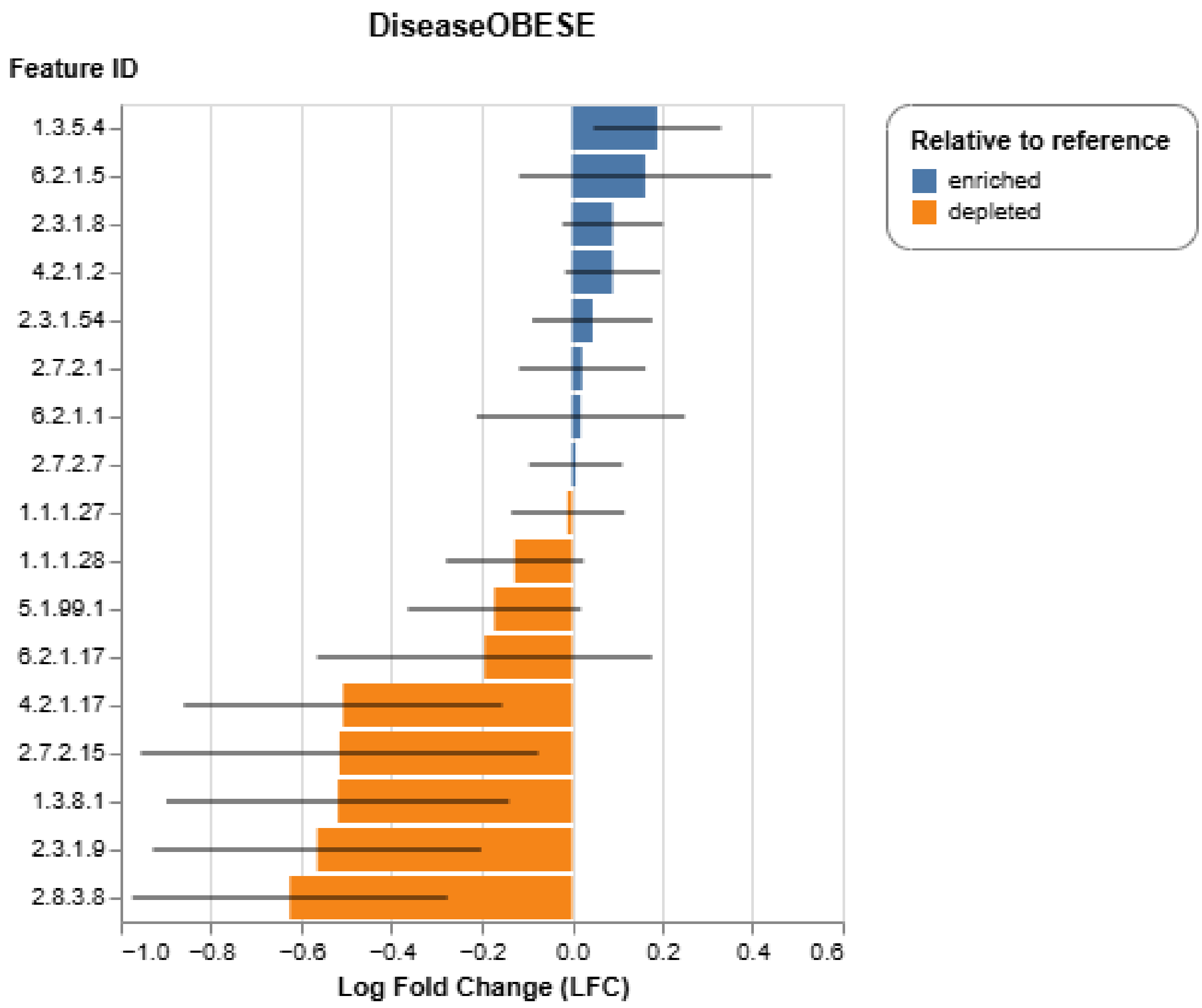

Supplementary figure 2. Functional assessment of SCFA production in the obese Tunisian group.

ACETATE (2.3.1.8- 2.7.2.1- 6.2.1.1), BUTYRATE (2.3.1.9 - 1.3.8.1- 2.8.3.8 - 2.7.2.7), PROPIONATE (6.2.1.17- 5.1.99.1 - 4.2.1.17 - 2.7.2.15) , LACTATE (1.1.1.27 - 1.1.1.28) , SUCCINATE (1.3.5.4 - 4.2.1.2 - 6.2.1.5) , FORMATE (2.3.1.54 - 1.2.1.2 - 1.17.98.3)

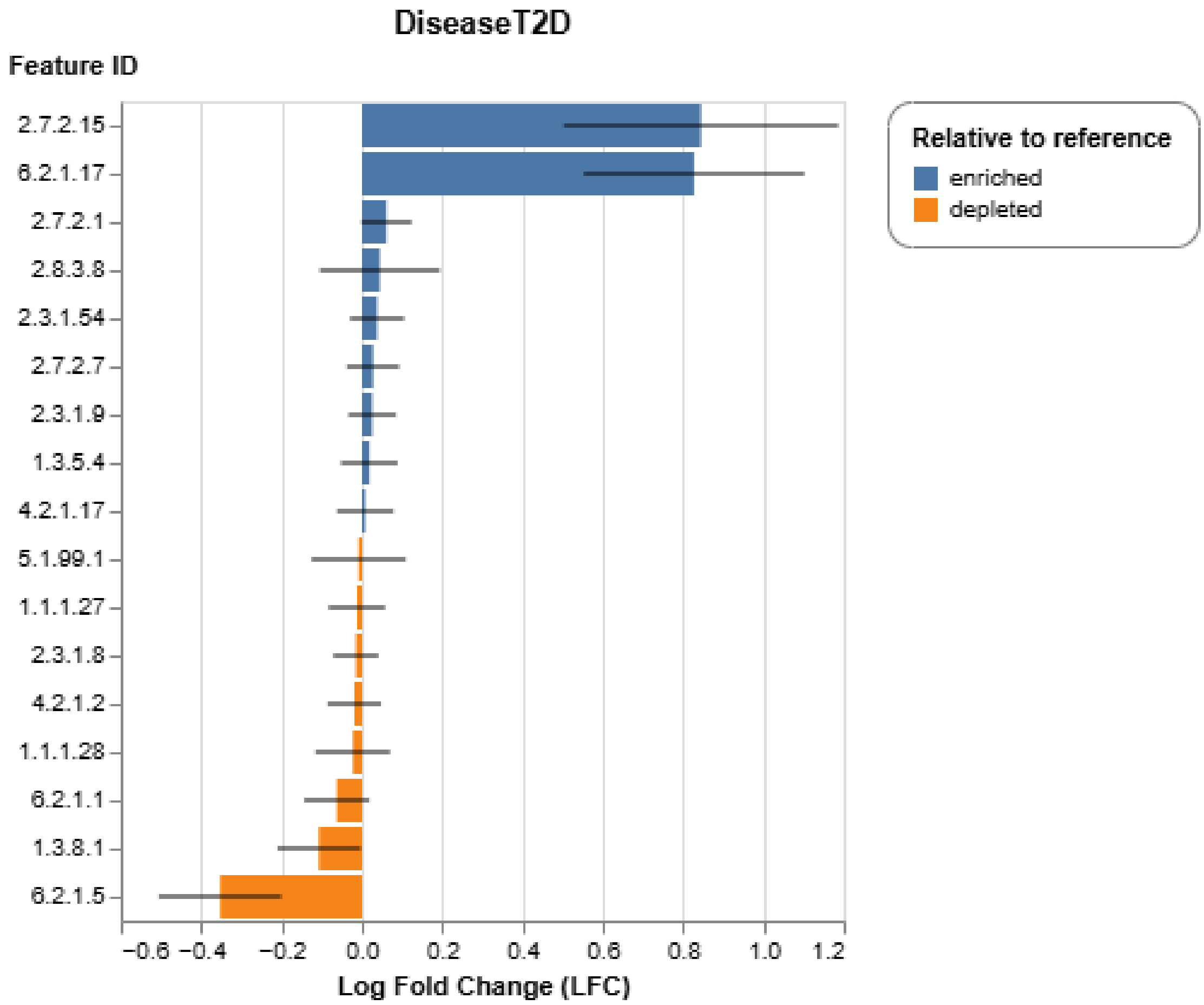

Supplementary figure 3. Evaluation of SCFAs production potential in the T2D Tunisian group.

ACETATE (2.3.1.8- 2.7.2.1- 6.2.1.1), BUTYRATE (2.3.1.9 - 1.3.8.1- 2.8.3.8 - 2.7.2.7), PROPIONATE (6.2.1.17- 5.1.99.1 - 4.2.1.17 - 2.7.2.15) , LACTATE (1.1.1.27 - 1.1.1.28) , SUCCINATE (1.3.5.4 - 4.2.1.2 - 6.2.1.5) , FORMATE (2.3.1.54 - 1.2.1.2 - 1.17.98.3)

T2D type 2 diabetic patients

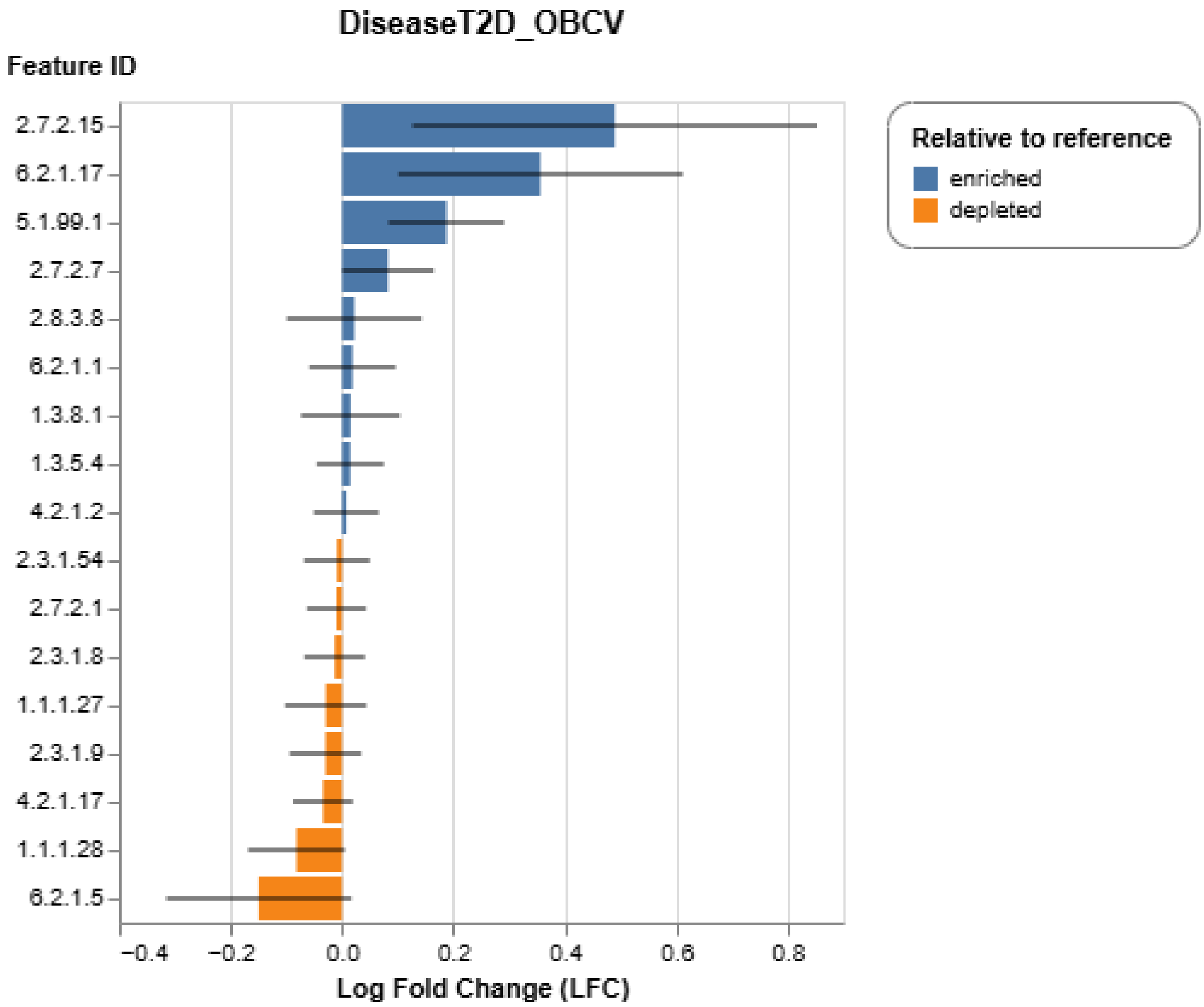

Supplementary figure 4. Assessment of SCFAs production potential in the T2D_OBCV Tunisian group.

ACETATE (2.3.1.8- 2.7.2.1- 6.2.1.1), BUTYRATE (2.3.1.9 - 1.3.8.1- 2.8.3.8 - 2.7.2.7), PROPIONATE (6.2.1.17- 5.1.99.1 - 4.2.1.17 - 2.7.2.15) , LACTATE (1.1.1.27 - 1.1.1.28) , SUCCINATE (1.3.5.4 - 4.2.1.2 - 6.2.1.5) , FORMATE (2.3.1.54 - 1.2.1.2 - 1.17.98.3)

T2D_OBCV type 2 diabetic patients with obesity and cardiovascular complications.

Supplementary Table 1. Clinical Characteristics of participants

Patients CODES	Clinical status	Sex	Age	BMIs (kg/m-2)	Glucose (mmol/l)	HbA1c (%)
DBM-C1-010_S9	ND	M	41	25,61	5,16	5,54
DBM-C1-029_S8	ND	F	58	22,31	5,93	5,77
DBM-C1-033_S53	ND	F	40	25,2	4,71	4,92
DBM-C1-038_S16	ND	F	36	23,3	5,16	5,11
DBM-C1-041_S2	ND	M	45	29,4	6,21	5,1
DBM-C1-042_S1	ND	F	44	27,43	4,77	5,1
DBM-C1-044_S50	ND	F	44	29,38	4,88	5,21
DBM-C1-046_S28	ND	F	59	24,5	5,21	5,67
DBM-C1-047_S45	ND	F	54	23,6	5,49	5,49
DBM-C1-048_S51	ND	F	31	29,37	5,49	5,27
DBM-C1-049_S43	ND	M	64	29,7	5,49	5,19
DBM-C1-052_S4	ND	M	57	26,49	5,27	5,79
DBM-C1-054_S15	ND	F	36	24,52	5,05	5,01
DBM-C1-057_S19	ND	F	47	23,33	4,99	5,57
DBM-C1-058_S23	ND	F	56	22,15	5,21	5,75
DBM-C1-065_S26	ND	M	42	26,11	5,55	5,44
DBM-C1-071_S42	ND	F	42	22,01	4,99	5,5
DBM-C1-075_S13	ND	F	47	23,73	4,82	5,21
DBM-C1-078_S47	ND	F	64	24,7	6,1	5,8
DBM-C1-081_S48	ND	F	54	22,81	5,43	5,54
DBM-C1-090_S48	ND	F	58	25	6,1	5,72
DBM-C1-094_S16	ND	F	46	19,68	5,38	4,68
DBM-C1-099_S29	ND	M	46	18	5,55	6,17
DBM-C1-100_S20	ND	M	71	24,9	5,1	6,46
DBM-C1-108_S17	ND	F	49	22,94	4,66	5,62
DBM-C1-114_S43	ND	M	42	22,58	5,16	5,89
DBM-C1-123_S52	ND	M	43	23,1	5,38	5,35
DBM-C1-132_S50	ND	M	69	22,038	5,88	6,8
DBM-C1-133_S20	ND	F	60	26,7	6,16	6,38
DBM-C1-134_S1	ND	F	60	24,52	6,27	6,57
DBM-C1-138_S39	ND	M	71	24,09	5,05	5,8
DBM-C1-061_S10	ND	F	46	22,5	5	4,5
DBM-C1-140_S12	ND	M	61	24,074	5,32	5,57
DBM-C1-030_S22	OBESE	F	30	33,05	4,99	5,42
DBM-C1-036_S49	OBESE	M	50	32,79	5,16	5,67
DBM-C1-062_S35	OBESE	F	46	30,1	4,88	5,79
DBM-C1-066_S33	OBESE	F	43	30,5	5,49	5,64

DBM-C1-067_S40	OBESE	F	52	31,25	5,99	5,14
DBM-C1-070_S56	OBESE	F	58	31,611	5,38	5,07
DBM-C1-072_S6	OBESE	M	42	37,64	4,82	5,46
DBM-C1-074_S17	OBESE	F	61	33,694	4,99	5,42
DBM-C1-084_S53	OBESE	M	55	34,31	4,82	5,47
DBM-C1-096_S52	OBESE	M	49	30,02	5,1	6,3
DBM-C1-001_S21	T2D	M	59	25,78	9,87	7,82
DBM-C1-002_S7	T2D	M	66	29,41	7,06	7,14
DBM-C1-004_S5	T2D	M	66	28,68	6,54	9,09
DBM-C1-005_S6	T2D	M	58	25,9	9,71	8,17
DBM-C1-008_S41	T2D	M	57	27,25	8,6	6,39
DBM-C1-015_S39	T2D	F	63	26,8	6,99	8,22
DBM-C1-017_S36	T2D	M	46	25,35	6,6	12,6
DBM-C1-018_S7	T2D	M	54	26,29	15,6	12,7
DBM-C1-025_S37	T2D	M	53	28,73	6,1	9,5
DBM-C1-026_S9	T2D	M	60	26,49	16	9,69
DBM-C1-028_S30	T2D	F	56	29,38	9,76	9,51
DBM-C1-045_S3	T2D	M	66	24,69	14,3	9,94
DBM-C1-053_S59	T2D	F	77	26,5	6,99	6,92
DBM-C1-060_S44	T2D	F	69	22,72	7,49	7,53
DBM-C1-080_S55	T2D	M	66	28,5	5,99	6,16
DBM-C1-082_S25	T2D	M	57	27,22	8,99	7,65
DBM-C1-089_S26	T2D	F	57	23,11	9,71	7,78
DBM-C1-091_S28	T2D	M	72	26,5	8,6	6,58
DBM-C1-105_S42	T2D	M	60	25,22	10,6	8,42
DBM-C1-107_S60	T2D	F	60	26,8	16,2	12,9
DBM-C1-111_S29	T2D	M	48	28,9	7,1	6,93
DBM-C1-115_S25	T2D	M	66	29,41	6,16	8,11
DBM-C1-121_S38	T2D	M	48	28,37	9,99	9,13
DBM-C1-124_S30	T2D	F	66	25,2	6,27	5,65
DBM-C1-131_S8	T2D	F	59	27,34	12,5	10,7
DBM-C1-142_S36	T2D	F	55	25,7	7,04	6,56
DBM-C1-003_S10	T2D_OBCV	M	57	30,2	7,43	6,96
DBM-C1-116_S27	T2D_OBCV	F	57	30,1	6,6	7,05
DBM-C1-126_S34	T2D_OBCV	M	72	30,6	16,1	7,6
DBM-C1-135_S41	T2D_OBCV	F	63	30,9	10,4	9,62
DBM-C1-012_S32	T2D_OBCV	F	65	34,29	8,26	6,21
DBM-C1-013_S31	T2D_OBCV	M	50	32,71	6,93	7,74
DBM-C1-014_S22	T2D_OBCV	M	51	32,9	7,99	6,52
DBM-C1-019_S12	T2D_OBCV	M	63	31,55	8,93	7,75
DBM-C1-020_S31	T2D_OBCV	F	62	30	9,82	10,5

DBM-C1-021_S56	T2D_OBCV	F	66	30,5	4,6	7,63
DBM-C1-022_S58	T2D_OBCV	F	63	32,4	12	8,71
DBM-C1-023_S14	T2D_OBCV	F	76	32,89	9,49	7,31
DBM-C1-024_S33	T2D_OBCV	M	66	37,75	10,4	8,06
DBM-C1-034_S19	T2D_OBCV	F	50	31,221	11,1	9,86
DBM-C1-050_S45	T2D_OBCV	F	57	32,27	7,77	7,31
DBM-C1-073_S57	T2D_OBCV	F	66	34,69	7,88	7,93
DBM-C1-125_S23	T2D_OBCV	F	57	30,46	8,38	9,15
DBM-C1-130_S4	T2D_OBCV	F	50	32,5	13,2	12,3
DBM-C1-137_S18	T2D_OBCV	F	61	30,46	16,4	10,7
DBM-C1-139_S11	T2D_OBCV	F	50	38	7,71	8,81
DBM-C1-141_S21	T2D_OBCV	F	51	37,63	9,6	9,84
DBM-C1-143_S40	T2D_OBCV	F	52	30,8	10,9	6,77
DBM-C1-144_S35	T2D_OBCV	M	59	30,5	7,82	7,61
DBM-C1-009_S27	T2D_OBCV	M	56	31,88	13,2	9,46
DBM-C1-112_S57	T2D_OBCV	M	73	32,4	10,6	13,4
DBM-C1-147_S14	T2D_OBCV	M	55	36,4	9,71	8,66

BMI : Body Mass Index, HbA1c : Hemoglobin A1c

Supplementary table 2 Adonis test results

	Df	SumsOfSqs	MeanSqs	F.Model	R2	Pr(>F)
Sex	1	0.245	0.245	0.943	0.010	0.461
Age	36	8.881	0.246	0.948	0.370	0.754
Oral Antidiabetic Agent	1	0.365	0.365	1.403	0.015	0.114
Insulin treatment	1	0.141	0.141	0.542	0.005	0.962

Df : Degrees of freedom associated with the factor

SumsOfSqs : Sum of squares attributable to that factor

MeanSqs : Mean square, calculated as **SumsOfSqs / Df**.

F.Model : Pseudo-F statistic, comparing variation among groups to variation within groups

R^2 : Coefficient of determination

Pr(>F) : P-value obtained by permutation testing.

Study SRP512155: HUMAN GUT METAGENOME Raw sequence reads

Sample SRS21535626: gut metagenome
Experiment SRX24822309: Metagenomic study : Human gut microbiota
Run SRR29305354 with 14645678 bases and 24487 spots

Sample SRS21535627: gut metagenome
Experiment SRX24822310: Metagenomic study : Human gut microbiota
Run SRR29305353 with 42529546 bases and 70900 spots

Sample SRS21535628: gut metagenome
Experiment SRX24822311: Metagenomic study : Human gut microbiota
Run SRR29305352 with 135112895 bases and 225046 spots

Sample SRS21535629: gut metagenome
Experiment SRX24822312: Metagenomic study : Human gut microbiota
Run SRR29305351 with 288635766 bases and 480821 spots

Sample SRS21535630: gut metagenome
Experiment SRX24822313: Metagenomic study : Human gut microbiota
Run SRR29305350 with 40369289 bases and 67290 spots

Sample SRS21535631: gut metagenome
Experiment SRX24822314: Metagenomic study : Human gut microbiota
Run SRR29305349 with 147223167 bases and 245353 spots

Sample SRS21535632: gut metagenome
Experiment SRX24822315: Metagenomic study : Human gut microbiota
Run SRR29305348 with 295494700 bases and 493265 spots

Sample SRS21535633: gut metagenome
Experiment SRX24822316: Metagenomic study : Human gut microbiota
Run SRR29305347 with 64715162 bases and 107786 spots

Sample SRS21535634: gut metagenome
Experiment SRX24822317: Metagenomic study : Human gut microbiota
Run SRR29305346 with 48763675 bases and 81202 spots

Sample SRS21535635: gut metagenome
Experiment SRX24822318: Metagenomic study : Human gut microbiota
Run SRR29305345 with 389211753 bases and 648605 spots

Sample SRS21535636: gut metagenome
Experiment SRX24822319: Metagenomic study : Human gut microbiota
Run SRR29305344 with 168799037 bases and 281149 spots

Sample SRS21535637: gut metagenome
Experiment SRX24822320: Metagenomic study : Human gut microbiota
Run SRR29305343 with 32947321 bases and 54983 spots

Sample SRS21535638: gut metagenome
Experiment SRX24822321: Metagenomic study : Human gut microbiota
Run SRR29305342 with 185728214 bases and 309296 spots

Sample SRS21535639: gut metagenome
Experiment SRX24822322: Metagenomic study : Human gut microbiota
Run SRR29305341 with 269987403 bases and 449922 spots

Sample SRS21535640: gut metagenome
Experiment SRX24822323: Metagenomic study : Human gut microbiota
Run SRR29305340 with 35913997 bases and 60351 spots

Sample SRS21535641: gut metagenome
Experiment SRX24822324: Metagenomic study : Human gut microbiota
Run SRR29305339 with 48698229 bases and 81087 spots

Sample SRS21535642: gut metagenome
Experiment SRX24822325: Metagenomic study : Human gut microbiota
Run SRR29305338 with 336821234 bases and 561381 spots

```
Sample SRS21535643: gut metagenome
        Experiment SRX24822326: Metagenomic study : Human gut microbiota
                Run SRR29305337 with 39342163 bases and 65802 spots

Sample SRS21535644: gut metagenome
        Experiment SRX24822327: Metagenomic study : Human gut microbiota
                Run SRR29305336 with 165060585 bases and 274966 spots

Sample SRS21535645: gut metagenome
        Experiment SRX24822328: Metagenomic study : Human gut microbiota
                Run SRR29305335 with 242806163 bases and 404775 spots

Sample SRS21535646: gut metagenome
        Experiment SRX24822329: Metagenomic study : Human gut microbiota
                Run SRR29305334 with 184601610 bases and 307841 spots

Sample SRS21535647: gut metagenome
        Experiment SRX24822330: Metagenomic study : Human gut microbiota
                Run SRR29305333 with 60663628 bases and 102060 spots

Sample SRS21535648: gut metagenome
        Experiment SRX24822331: Metagenomic study : Human gut microbiota
                Run SRR29305332 with 73661547 bases and 122710 spots

Sample SRS21535649: gut metagenome
        Experiment SRX24822332: Metagenomic study : Human gut microbiota
                Run SRR29305331 with 17868944 bases and 29771 spots

Sample SRS21535650: gut metagenome
        Experiment SRX24822333: Metagenomic study : Human gut microbiota
                Run SRR29305330 with 193195632 bases and 322118 spots

Sample SRS21535651: gut metagenome
        Experiment SRX24822334: Metagenomic study : Human gut microbiota
                Run SRR29305329 with 324182856 bases and 539851 spots

Sample SRS21535652: gut metagenome
        Experiment SRX24822335: Metagenomic study : Human gut microbiota
                Run SRR29305328 with 205018607 bases and 341730 spots

Sample SRS21535653: gut metagenome
        Experiment SRX24822336: Metagenomic study : Human gut microbiota
                Run SRR29305327 with 64684252 bases and 107855 spots

Sample SRS21535654: gut metagenome
        Experiment SRX24822337: Metagenomic study : Human gut microbiota
                Run SRR29305326 with 26947284 bases and 44915 spots

Sample SRS21535655: gut metagenome
        Experiment SRX24822338: Metagenomic study : Human gut microbiota
                Run SRR29305325 with 80499795 bases and 134249 spots

Sample SRS21535656: gut metagenome
        Experiment SRX24822339: Metagenomic study : Human gut microbiota
                Run SRR29305324 with 228499931 bases and 381254 spots

Sample SRS21535657: gut metagenome
        Experiment SRX24822340: Metagenomic study : Human gut microbiota
                Run SRR29305323 with 242919630 bases and 404728 spots

Sample SRS21535658: gut metagenome
        Experiment SRX24822341: Metagenomic study : Human gut microbiota
                Run SRR29305322 with 59664351 bases and 99374 spots

Sample SRS21535659: gut metagenome
        Experiment SRX24822342: Metagenomic study : Human gut microbiota
                Run SRR29305321 with 36265238 bases and 60943 spots
```

```
Sample SRS21535660: gut metagenome
        Experiment SRX24822343: Metagenomic study : Human gut microbiota
                Run SRR29305320 with 209968637 bases and 350128 spots

Sample SRS21535661: gut metagenome
        Experiment SRX24822344: Metagenomic study : Human gut microbiota
                Run SRR29305319 with 95418292 bases and 159062 spots

Sample SRS21535662: gut metagenome
        Experiment SRX24822345: Metagenomic study : Human gut microbiota
                Run SRR29305318 with 56512506 bases and 94257 spots

Sample SRS21535663: gut metagenome
        Experiment SRX24822346: Metagenomic study : Human gut microbiota
                Run SRR29305317 with 137152141 bases and 228584 spots

Sample SRS21535664: gut metagenome
        Experiment SRX24822347: Metagenomic study : Human gut microbiota
                Run SRR29305316 with 283875141 bases and 475093 spots

Sample SRS21535665: gut metagenome
        Experiment SRX24822348: Metagenomic study : Human gut microbiota
                Run SRR29305315 with 351865482 bases and 588447 spots

Sample SRS21535666: gut metagenome
        Experiment SRX24822349: Metagenomic study : Human gut microbiota
                Run SRR29305314 with 116915625 bases and 195432 spots

Sample SRS21535667: gut metagenome
        Experiment SRX24822350: Metagenomic study : Human gut microbiota
                Run SRR29305313 with 94384408 bases and 157353 spots

Sample SRS21535668: gut metagenome
        Experiment SRX24822351: Metagenomic study : Human gut microbiota
                Run SRR29305312 with 66040710 bases and 109988 spots

Sample SRS21535669: gut metagenome
        Experiment SRX24822352: Metagenomic study : Human gut microbiota
                Run SRR29305311 with 156215303 bases and 260448 spots

Sample SRS21535670: gut metagenome
        Experiment SRX24822353: Metagenomic study : Human gut microbiota
                Run SRR29305310 with 11765906 bases and 19668 spots

Sample SRS21535671: gut metagenome
        Experiment SRX24822354: Metagenomic study : Human gut microbiota
                Run SRR29305309 with 62998386 bases and 104943 spots

Sample SRS21535672: gut metagenome
        Experiment SRX24822355: Metagenomic study : Human gut microbiota
                Run SRR29305308 with 380254491 bases and 633324 spots

Sample SRS21535673: gut metagenome
        Experiment SRX24822356: Metagenomic study : Human gut microbiota
                Run SRR29305307 with 542891204 bases and 904709 spots

Sample SRS21535674: gut metagenome
        Experiment SRX24822357: Metagenomic study : Human gut microbiota
                Run SRR29305306 with 97084537 bases and 161852 spots

Sample SRS21535675: gut metagenome
        Experiment SRX24822358: Metagenomic study : Human gut microbiota
                Run SRR29305305 with 92649098 bases and 154506 spots

Sample SRS21535676: gut metagenome
        Experiment SRX24822359: Metagenomic study : Human gut microbiota
                Run SRR29305304 with 314920381 bases and 524888 spots

Sample SRS21535677: gut metagenome
```

Experiment SRX24822360: Metagenomic study : Human gut microbiota
Run SRR29305303 with 66554160 bases and 110965 spots

Sample SRS21535678: gut metagenome
Experiment SRX24822361: Metagenomic study : Human gut microbiota
Run SRR29305302 with 144824226 bases and 241262 spots

Sample SRS21535679: gut metagenome
Experiment SRX24822362: Metagenomic study : Human gut microbiota
Run SRR29305301 with 170576914 bases and 284381 spots

Sample SRS21535680: gut metagenome
Experiment SRX24822363: Metagenomic study : Human gut microbiota
Run SRR29305300 with 186764587 bases and 311464 spots

Sample SRS21535681: gut metagenome
Experiment SRX24822364: Metagenomic study : Human gut microbiota
Run SRR29305299 with 60577131 bases and 101029 spots

Sample SRS21535682: gut metagenome
Experiment SRX24822365: Metagenomic study : Human gut microbiota
Run SRR29305298 with 443707142 bases and 739587 spots

Sample SRS21535683: gut metagenome
Experiment SRX24822366: Metagenomic study : Human gut microbiota
Run SRR29305297 with 7918202 bases and 13201 spots

Sample SRS21535684: gut metagenome
Experiment SRX24822367: Metagenomic study : Human gut microbiota
Run SRR29305296 with 124235591 bases and 207645 spots

Sample SRS21535685: gut metagenome
Experiment SRX24822368: Metagenomic study : Human gut microbiota
Run SRR29305295 with 71648830 bases and 119433 spots

Sample SRS21535686: gut metagenome
Experiment SRX24822369: Metagenomic study : Human gut microbiota
Run SRR29305294 with 85200901 bases and 141984 spots

Sample SRS21535687: gut metagenome
Experiment SRX24822370: Metagenomic study : Human gut microbiota
Run SRR29305293 with 289777724 bases and 482415 spots

Sample SRS21535688: gut metagenome
Experiment SRX24822371: Metagenomic study : Human gut microbiota
Run SRR29305292 with 71122380 bases and 118508 spots

Sample SRS21535689: gut metagenome
Experiment SRX24822372: Metagenomic study : Human gut microbiota
Run SRR29305291 with 257328545 bases and 428813 spots

Sample SRS21535690: gut metagenome
Experiment SRX24822373: Metagenomic study : Human gut microbiota
Run SRR29305290 with 263222442 bases and 438827 spots

Sample SRS21535691: gut metagenome
Experiment SRX24822374: Metagenomic study : Human gut microbiota
Run SRR29305289 with 142051115 bases and 236556 spots

Sample SRS21535692: gut metagenome
Experiment SRX24822375: Metagenomic study : Human gut microbiota
Run SRR29305288 with 643752768 bases and 1074309 spots

Sample SRS21535693: gut metagenome
Experiment SRX24822376: Metagenomic study : Human gut microbiota
Run SRR29305287 with 283511650 bases and 473562 spots

Sample SRS21535694: gut metagenome
Experiment SRX24822377: Metagenomic study : Human gut microbiota

Run SRR29305286 with 242783746 bases and 404531 spots

Sample SRS21535695: gut metagenome
Experiment SRX24822378: Metagenomic study : Human gut microbiota
Run SRR29305285 with 62358662 bases and 103973 spots

Sample SRS21535696: gut metagenome
Experiment SRX24822379: Metagenomic study : Human gut microbiota
Run SRR29305284 with 212209183 bases and 353591 spots

Sample SRS21535697: gut metagenome
Experiment SRX24822380: Metagenomic study : Human gut microbiota
Run SRR29305283 with 21326280 bases and 35519 spots

Sample SRS21535698: gut metagenome
Experiment SRX24822381: Metagenomic study : Human gut microbiota
Run SRR29305282 with 34427454 bases and 57439 spots

Sample SRS21535699: gut metagenome
Experiment SRX24822382: Metagenomic study : Human gut microbiota
Run SRR29305281 with 109084573 bases and 182614 spots

Sample SRS21535700: gut metagenome
Experiment SRX24822383: Metagenomic study : Human gut microbiota
Run SRR29305280 with 328947933 bases and 547958 spots

Sample SRS21535701: gut metagenome
Experiment SRX24822384: Metagenomic study : Human gut microbiota
Run SRR29305279 with 163444659 bases and 272289 spots

Sample SRS21535702: gut metagenome
Experiment SRX24822385: Metagenomic study : Human gut microbiota
Run SRR29305278 with 7794253 bases and 13055 spots

Sample SRS21535703: gut metagenome
Experiment SRX24822386: Metagenomic study : Human gut microbiota
Run SRR29305277 with 51396533 bases and 85610 spots

Sample SRS21535704: gut metagenome
Experiment SRX24822387: Metagenomic study : Human gut microbiota
Run SRR29305276 with 20405198 bases and 34009 spots

Sample SRS21535705: gut metagenome
Experiment SRX24822388: Metagenomic study : Human gut microbiota
Run SRR29305275 with 89455913 bases and 149044 spots

Sample SRS21535706: gut metagenome
Experiment SRX24822389: Metagenomic study : Human gut microbiota
Run SRR29305274 with 49995725 bases and 83241 spots

Sample SRS21535707: gut metagenome
Experiment SRX24822390: Metagenomic study : Human gut microbiota
Run SRR29305273 with 59401170 bases and 99520 spots

Sample SRS21535708: gut metagenome
Experiment SRX24822391: Metagenomic study : Human gut microbiota
Run SRR29305272 with 127283603 bases and 212049 spots

Sample SRS21535709: gut metagenome
Experiment SRX24822392: Metagenomic study : Human gut microbiota
Run SRR29305271 with 68171236 bases and 113688 spots

Sample SRS21535710: gut metagenome
Experiment SRX24822393: Metagenomic study : Human gut microbiota
Run SRR29305270 with 128725235 bases and 214975 spots

Sample SRS21535711: gut metagenome
Experiment SRX24822394: Metagenomic study : Human gut microbiota
Run SRR29305269 with 151787099 bases and 252975 spots

```
Sample SRS21535712: gut metagenome
        Experiment SRX24822395: Metagenomic study : Human gut microbiota
                Run SRR29305268 with 134702674 bases and 224425 spots

Sample SRS21535713: gut metagenome
        Experiment SRX24822396: Metagenomic study : Human gut microbiota
                Run SRR29305267 with 97662498 bases and 162977 spots

Sample SRS21535714: gut metagenome
        Experiment SRX24822397: Metagenomic study : Human gut microbiota
                Run SRR29305266 with 32410354 bases and 54108 spots

Sample SRS21535715: gut metagenome
        Experiment SRX24822398: Metagenomic study : Human gut microbiota
                Run SRR29305265 with 326896666 bases and 545176 spots

Sample SRS21535716: gut metagenome
        Experiment SRX24822399: Metagenomic study : Human gut microbiota
                Run SRR29305264 with 363926572 bases and 609638 spots

Sample SRS21535717: gut metagenome
        Experiment SRX24822400: Metagenomic study : Human gut microbiota
                Run SRR29305263 with 74298708 bases and 123942 spots

Sample SRS21535718: gut metagenome
        Experiment SRX24822401: Metagenomic study : Human gut microbiota
                Run SRR29305262 with 278458224 bases and 464777 spots

Sample SRS21535719: gut metagenome
        Experiment SRX24822402: Metagenomic study : Human gut microbiota
                Run SRR29305261 with 30608230 bases and 51041 spots

Sample SRS21535720: gut metagenome
        Experiment SRX24822403: Metagenomic study : Human gut microbiota
                Run SRR29305260 with 53208556 bases and 88589 spots

Sample SRS21535721: gut metagenome
        Experiment SRX24822404: Metagenomic study : Human gut microbiota
                Run SRR29305259 with 285638397 bases and 476193 spots

Sample SRS21535722: gut metagenome
        Experiment SRX24822405: Metagenomic study : Human gut microbiota
                Run SRR29305258 with 66718051 bases and 111138 spots

Sample SRS21535723: gut metagenome
        Experiment SRX24822406: Metagenomic study : Human gut microbiota
                Run SRR29305257 with 71411002 bases and 118873 spots

Sample SRS21535724: gut metagenome
        Experiment SRX24822407: Metagenomic study : Human gut microbiota
                Run SRR29305256 with 84440564 bases and 140680 spots

Sample SRS21535725: gut metagenome
        Experiment SRX24822408: Metagenomic study : Human gut microbiota
                Run SRR29305255 with 178760520 bases and 298260 spots

Sample SRS21535726: gut metagenome
        Experiment SRX24822409: Metagenomic study : Human gut microbiota
                Run SRR29305254 with 169410833 bases and 282487 spots

Sample SRS21535727: gut metagenome
        Experiment SRX24822410: Metagenomic study : Human gut microbiota
                Run SRR29305253 with 248693888 bases and 414154 spots

Sample SRS21535728: gut metagenome
        Experiment SRX24822411: Metagenomic study : Human gut microbiota
                Run SRR29305252 with 199628814 bases and 332754 spots
```

```
Sample SRS21535729: gut metagenome
        Experiment SRX24822412: Metagenomic study : Human gut microbiota
                Run SRR29305251 with 65230570 bases and 108824 spots

Sample SRS21535730: gut metagenome
        Experiment SRX24822413: Metagenomic study : Human gut microbiota
                Run SRR29305250 with 55700522 bases and 92839 spots

Sample SRS21535731: gut metagenome
        Experiment SRX24822414: Metagenomic study : Human gut microbiota
                Run SRR29305249 with 31241594 bases and 52134 spots

Sample SRS21535732: gut metagenome
        Experiment SRX24822415: Metagenomic study : Human gut microbiota
                Run SRR29305248 with 357200630 bases and 595005 spots

Sample SRS21535733: gut metagenome
        Experiment SRX24822416: Metagenomic study : Human gut microbiota
                Run SRR29305247 with 27076091 bases and 45110 spots

Sample SRS21535734: gut metagenome
        Experiment SRX24822417: Metagenomic study : Human gut microbiota
                Run SRR29305246 with 314947645 bases and 526281 spots

Sample SRS21535735: gut metagenome
        Experiment SRX24822418: Metagenomic study : Human gut microbiota
                Run SRR29305245 with 76007283 bases and 126546 spots
```

Study	Sample	Experiment	Run	ExperimentTitle	LibraryStrategy	LibrarySource	LibrarySelection	LibraryLayout	Model	RunSpots	RunBases	BioSample	BioProject
SRP512155	SRS21535626	SRX24822309	SRR29305354	Metagenomic study : Human gut microbiota	AMPLICON	METAGENOMIC	PCR	PAIRED	Illumina MiSeq	24487	14645678	SAMN41705560	PRJNA1095229
SRP512155	SRS21535627	SRX24822310	SRR29305353	Metagenomic study : Human gut microbiota	AMPLICON	METAGENOMIC	PCR	PAIRED	Illumina MiSeq	70900	42529546	SAMN41705561	PRJNA1095229
SRP512155	SRS21535628	SRX24822311	SRR29305352	Metagenomic study : Human gut microbiota	AMPLICON	METAGENOMIC	PCR	PAIRED	Illumina MiSeq	225046	135112895	SAMN41705570	PRJNA1095229
SRP512155	SRS21535629	SRX24822312	SRR29305351	Metagenomic study : Human gut microbiota	AMPLICON	METAGENOMIC	PCR	PAIRED	Illumina MiSeq	480821	288635766	SAMN41705660	PRJNA1095229
SRP512155	SRS21535630	SRX24822313	SRR29305350	Metagenomic study : Human gut microbiota	AMPLICON	METAGENOMIC	PCR	PAIRED	Illumina MiSeq	67290	40369289	SAMN41705661	PRJNA1095229
SRP512155	SRS21535631	SRX24822314	SRR29305349	Metagenomic study : Human gut microbiota	AMPLICON	METAGENOMIC	PCR	PAIRED	Illumina MiSeq	245353	147223167	SAMN41705662	PRJNA1095229
SRP512155	SRS21535632	SRX24822315	SRR29305348	Metagenomic study : Human gut microbiota	AMPLICON	METAGENOMIC	PCR	PAIRED	Illumina MiSeq	493265	295494700	SAMN41705663	PRJNA1095229
SRP512155	SRS21535633	SRX24822316	SRR29305347	Metagenomic study : Human gut microbiota	AMPLICON	METAGENOMIC	PCR	PAIRED	Illumina MiSeq	107786	64715162	SAMN41705664	PRJNA1095229
SRP512155	SRS21535634	SRX24822317	SRR29305346	Metagenomic study : Human gut microbiota	AMPLICON	METAGENOMIC	PCR	PAIRED	Illumina MiSeq	81202	48763675	SAMN41705665	PRJNA1095229
SRP512155	SRS21535635	SRX24822318	SRR29305345	Metagenomic study : Human gut microbiota	AMPLICON	METAGENOMIC	PCR	PAIRED	Illumina MiSeq	648605	389211753	SAMN41705666	PRJNA1095229
SRP512155	SRS21535636	SRX24822319	SRR29305344	Metagenomic study : Human gut microbiota	AMPLICON	METAGENOMIC	PCR	PAIRED	Illumina MiSeq	281149	168799037	SAMN41705667	PRJNA1095229
SRP512155	SRS21535637	SRX24822320	SRR29305343	Metagenomic study : Human gut microbiota	AMPLICON	METAGENOMIC	PCR	PAIRED	Illumina MiSeq	54983	32947321	SAMN41705668	PRJNA1095229
SRP512155	SRS21535638	SRX24822321	SRR29305342	Metagenomic study : Human gut microbiota	AMPLICON	METAGENOMIC	PCR	PAIRED	Illumina MiSeq	309296	185728214	SAMN41705669	PRJNA1095229
SRP512155	SRS21535639	SRX24822322	SRR29305341	Metagenomic study : Human gut microbiota	AMPLICON	METAGENOMIC	PCR	PAIRED	Illumina MiSeq	449922	269987403	SAMN41705571	PRJNA1095229
SRP512155	SRS21535640	SRX24822323	SRR29305340	Metagenomic study : Human gut microbiota	AMPLICON	METAGENOMIC	PCR	PAIRED	Illumina MiSeq	60351	35913997	SAMN41705572	PRJNA1095229
SRP512155	SRS21535641	SRX24822324	SRR29305339	Metagenomic study : Human gut microbiota	AMPLICON	METAGENOMIC	PCR	PAIRED	Illumina MiSeq	81087	48698229	SAMN41705573	PRJNA1095229
SRP512155	SRS21535642	SRX24822325	SRR29305338	Metagenomic study : Human gut microbiota	AMPLICON	METAGENOMIC	PCR	PAIRED	Illumina MiSeq	561381	336821234	SAMN41705574	PRJNA1095229
SRP512155	SRS21535643	SRX24822326	SRR29305337	Metagenomic study : Human gut microbiota	AMPLICON	METAGENOMIC	PCR	PAIRED	Illumina MiSeq	65802	39342163	SAMN41705575	PRJNA1095229
SRP512155	SRS21535644	SRX24822327	SRR29305336	Metagenomic study : Human gut microbiota	AMPLICON	METAGENOMIC	PCR	PAIRED	Illumina MiSeq	274966	165060585	SAMN41705576	PRJNA1095229
SRP512155	SRS21535645	SRX24822328	SRR29305335	Metagenomic study : Human gut microbiota	AMPLICON	METAGENOMIC	PCR	PAIRED	Illumina MiSeq	404775	242806163	SAMN41705577	PRJNA1095229
SRP512155	SRS21535646	SRX24822329	SRR29305334	Metagenomic study : Human gut microbiota	AMPLICON	METAGENOMIC	PCR	PAIRED	Illumina MiSeq	307841	184601610	SAMN41705578	PRJNA1095229
SRP512155	SRS21535647	SRX24822330	SRR29305333	Metagenomic study : Human gut microbiota	AMPLICON	METAGENOMIC	PCR	PAIRED	Illumina MiSeq	102060	60663628	SAMN41705579	PRJNA1095229

SRP512155	SRS21535648	SRX24822331	SRR29305332	Metagenomic study : Human gut microbiota	AMPLICON	METAGENOMIC	PCR	PAIRED	Illumina MiSeq	122710	73661547	SAMN41705562	PRJNA1095229
SRP512155	SRS21535649	SRX24822332	SRR29305331	Metagenomic study : Human gut microbiota	AMPLICON	METAGENOMIC	PCR	PAIRED	Illumina MiSeq	29771	17868944	SAMN41705580	PRJNA1095229
SRP512155	SRS21535650	SRX24822333	SRR29305330	Metagenomic study : Human gut microbiota	AMPLICON	METAGENOMIC	PCR	PAIRED	Illumina MiSeq	322118	193195632	SAMN41705581	PRJNA1095229
SRP512155	SRS21535651	SRX24822334	SRR29305329	Metagenomic study : Human gut microbiota	AMPLICON	METAGENOMIC	PCR	PAIRED	Illumina MiSeq	539851	324182856	SAMN41705582	PRJNA1095229
SRP512155	SRS21535652	SRX24822335	SRR29305328	Metagenomic study : Human gut microbiota	AMPLICON	METAGENOMIC	PCR	PAIRED	Illumina MiSeq	341730	205018607	SAMN41705583	PRJNA1095229
SRP512155	SRS21535653	SRX24822336	SRR29305327	Metagenomic study : Human gut microbiota	AMPLICON	METAGENOMIC	PCR	PAIRED	Illumina MiSeq	107855	64684252	SAMN41705584	PRJNA1095229
SRP512155	SRS21535654	SRX24822337	SRR29305326	Metagenomic study : Human gut microbiota	AMPLICON	METAGENOMIC	PCR	PAIRED	Illumina MiSeq	44915	26947284	SAMN41705585	PRJNA1095229
SRP512155	SRS21535655	SRX24822338	SRR29305325	Metagenomic study : Human gut microbiota	AMPLICON	METAGENOMIC	PCR	PAIRED	Illumina MiSeq	134249	80499795	SAMN41705586	PRJNA1095229
SRP512155	SRS21535656	SRX24822339	SRR29305324	Metagenomic study : Human gut microbiota	AMPLICON	METAGENOMIC	PCR	PAIRED	Illumina MiSeq	381254	228499931	SAMN41705587	PRJNA1095229
SRP512155	SRS21535657	SRX24822340	SRR29305323	Metagenomic study : Human gut microbiota	AMPLICON	METAGENOMIC	PCR	PAIRED	Illumina MiSeq	404728	242919630	SAMN41705588	PRJNA1095229
SRP512155	SRS21535658	SRX24822341	SRR29305322	Metagenomic study : Human gut microbiota	AMPLICON	METAGENOMIC	PCR	PAIRED	Illumina MiSeq	99374	59664351	SAMN41705647	PRJNA1095229
SRP512155	SRS21535659	SRX24822342	SRR29305321	Metagenomic study : Human gut microbiota	AMPLICON	METAGENOMIC	PCR	PAIRED	Illumina MiSeq	60943	36265238	SAMN41705648	PRJNA1095229
SRP512155	SRS21535660	SRX24822343	SRR29305320	Metagenomic study : Human gut microbiota	AMPLICON	METAGENOMIC	PCR	PAIRED	Illumina MiSeq	350128	209968637	SAMN41705649	PRJNA1095229
SRP512155	SRS21535661	SRX24822344	SRR29305319	Metagenomic study : Human gut microbiota	AMPLICON	METAGENOMIC	PCR	PAIRED	Illumina MiSeq	159062	95418292	SAMN41705569	PRJNA1095229
SRP512155	SRS21535662	SRX24822345	SRR29305318	Metagenomic study : Human gut microbiota	AMPLICON	METAGENOMIC	PCR	PAIRED	Illumina MiSeq	94257	56512506	SAMN41705650	PRJNA1095229
SRP512155	SRS21535663	SRX24822346	SRR29305317	Metagenomic study : Human gut microbiota	AMPLICON	METAGENOMIC	PCR	PAIRED	Illumina MiSeq	228584	137152141	SAMN41705651	PRJNA1095229
SRP512155	SRS21535664	SRX24822347	SRR29305316	Metagenomic study : Human gut microbiota	AMPLICON	METAGENOMIC	PCR	PAIRED	Illumina MiSeq	475093	283875141	SAMN41705652	PRJNA1095229
SRP512155	SRS21535665	SRX24822348	SRR29305315	Metagenomic study : Human gut microbiota	AMPLICON	METAGENOMIC	PCR	PAIRED	Illumina MiSeq	588447	351865482	SAMN41705653	PRJNA1095229
SRP512155	SRS21535666	SRX24822349	SRR29305314	Metagenomic study : Human gut microbiota	AMPLICON	METAGENOMIC	PCR	PAIRED	Illumina MiSeq	195432	116915625	SAMN41705654	PRJNA1095229
SRP512155	SRS21535667	SRX24822350	SRR29305313	Metagenomic study : Human gut microbiota	AMPLICON	METAGENOMIC	PCR	PAIRED	Illumina MiSeq	157353	94384408	SAMN41705655	PRJNA1095229
SRP512155	SRS21535668	SRX24822351	SRR29305312	Metagenomic study : Human gut microbiota	AMPLICON	METAGENOMIC	PCR	PAIRED	Illumina MiSeq	109988	66040710	SAMN41705656	PRJNA1095229
SRP512155	SRS21535669	SRX24822352	SRR29305311	Metagenomic study : Human gut microbiota	AMPLICON	METAGENOMIC	PCR	PAIRED	Illumina MiSeq	260448	156215303	SAMN41705657	PRJNA1095229
SRP512155	SRS21535670	SRX24822353	SRR29305310	Metagenomic study : Human gut microbiota	AMPLICON	METAGENOMIC	PCR	PAIRED	Illumina MiSeq	19668	11765906	SAMN41705658	PRJNA1095229

SRP512155 SRS21535671 SRX24822354 SRR29305309 Metagenomic study : Human gut
microbiota AMPLICON METAGENOMIC PCR PAIRED Illumina MiSeq 104943 62998386
SAMN41705659 PRJNA1095229
SRP512155 SRS21535672 SRX24822355 SRR29305308 Metagenomic study : Human gut
microbiota AMPLICON METAGENOMIC PCR PAIRED Illumina MiSeq 633324 380254491
SAMN41705589 PRJNA1095229
SRP512155 SRS21535673 SRX24822356 SRR29305307 Metagenomic study : Human gut
microbiota AMPLICON METAGENOMIC PCR PAIRED Illumina MiSeq 904709 542891204
SAMN41705563 PRJNA1095229
SRP512155 SRS21535674 SRX24822357 SRR29305306 Metagenomic study : Human gut
microbiota AMPLICON METAGENOMIC PCR PAIRED Illumina MiSeq 161852 97084537
SAMN41705590 PRJNA1095229
SRP512155 SRS21535675 SRX24822358 SRR29305305 Metagenomic study : Human gut
microbiota AMPLICON METAGENOMIC PCR PAIRED Illumina MiSeq 154506 92649098
SAMN41705591 PRJNA1095229
SRP512155 SRS21535676 SRX24822359 SRR29305304 Metagenomic study : Human gut
microbiota AMPLICON METAGENOMIC PCR PAIRED Illumina MiSeq 524888 314920381
SAMN41705592 PRJNA1095229
SRP512155 SRS21535677 SRX24822360 SRR29305303 Metagenomic study : Human gut
microbiota AMPLICON METAGENOMIC PCR PAIRED Illumina MiSeq 110965 66554160
SAMN41705593 PRJNA1095229
SRP512155 SRS21535678 SRX24822361 SRR29305302 Metagenomic study : Human gut
microbiota AMPLICON METAGENOMIC PCR PAIRED Illumina MiSeq 241262 144824226
SAMN41705594 PRJNA1095229
SRP512155 SRS21535679 SRX24822362 SRR29305301 Metagenomic study : Human gut
microbiota AMPLICON METAGENOMIC PCR PAIRED Illumina MiSeq 284381 170576914
SAMN41705595 PRJNA1095229
SRP512155 SRS21535680 SRX24822363 SRR29305300 Metagenomic study : Human gut
microbiota AMPLICON METAGENOMIC PCR PAIRED Illumina MiSeq 311464 186764587
SAMN41705596 PRJNA1095229
SRP512155 SRS21535681 SRX24822364 SRR29305299 Metagenomic study : Human gut
microbiota AMPLICON METAGENOMIC PCR PAIRED Illumina MiSeq 101029 60577131
SAMN41705597 PRJNA1095229
SRP512155 SRS21535682 SRX24822365 SRR29305298 Metagenomic study : Human gut
microbiota AMPLICON METAGENOMIC PCR PAIRED Illumina MiSeq 739587 443707142
SAMN41705598 PRJNA1095229
SRP512155 SRS21535683 SRX24822366 SRR29305297 Metagenomic study : Human gut
microbiota AMPLICON METAGENOMIC PCR PAIRED Illumina MiSeq 13201 7918202
SAMN41705599 PRJNA1095229
SRP512155 SRS21535684 SRX24822367 SRR29305296 Metagenomic study : Human gut
microbiota AMPLICON METAGENOMIC PCR PAIRED Illumina MiSeq 207645 124235591
SAMN41705564 PRJNA1095229
SRP512155 SRS21535685 SRX24822368 SRR29305295 Metagenomic study : Human gut
microbiota AMPLICON METAGENOMIC PCR PAIRED Illumina MiSeq 119433 71648830
SAMN41705600 PRJNA1095229
SRP512155 SRS21535686 SRX24822369 SRR29305294 Metagenomic study : Human gut
microbiota AMPLICON METAGENOMIC PCR PAIRED Illumina MiSeq 141984 85200901
SAMN41705601 PRJNA1095229
SRP512155 SRS21535687 SRX24822370 SRR29305293 Metagenomic study : Human gut
microbiota AMPLICON METAGENOMIC PCR PAIRED Illumina MiSeq 482415 289777724
SAMN41705602 PRJNA1095229
SRP512155 SRS21535688 SRX24822371 SRR29305292 Metagenomic study : Human gut
microbiota AMPLICON METAGENOMIC PCR PAIRED Illumina MiSeq 118508 71122380
SAMN41705603 PRJNA1095229
SRP512155 SRS21535689 SRX24822372 SRR29305291 Metagenomic study : Human gut
microbiota AMPLICON METAGENOMIC PCR PAIRED Illumina MiSeq 428813 257328545
SAMN41705604 PRJNA1095229
SRP512155 SRS21535690 SRX24822373 SRR29305290 Metagenomic study : Human gut
microbiota AMPLICON METAGENOMIC PCR PAIRED Illumina MiSeq 438827 263222442
SAMN41705605 PRJNA1095229
SRP512155 SRS21535691 SRX24822374 SRR29305289 Metagenomic study : Human gut
microbiota AMPLICON METAGENOMIC PCR PAIRED Illumina MiSeq 236556 142051115
SAMN41705606 PRJNA1095229
SRP512155 SRS21535692 SRX24822375 SRR29305288 Metagenomic study : Human gut
microbiota AMPLICON METAGENOMIC PCR PAIRED Illumina MiSeq 1074309 643752768
SAMN41705607 PRJNA1095229
SRP512155 SRS21535693 SRX24822376 SRR29305287 Metagenomic study : Human gut
microbiota AMPLICON METAGENOMIC PCR PAIRED Illumina MiSeq 473562 283511650
SAMN41705608 PRJNA1095229

SRP512155 SRS21535694 SRX24822377 SRR29305286 Metagenomic study : Human gut
microbiota AMPLICON METAGENOMIC PCR PAIRED Illumina MiSeq 404531 242783746
SAMN41705609 PRJNA1095229
SRP512155 SRS21535695 SRX24822378 SRR29305285 Metagenomic study : Human gut
microbiota AMPLICON METAGENOMIC PCR PAIRED Illumina MiSeq 103973 62358662
SAMN41705565 PRJNA1095229
SRP512155 SRS21535696 SRX24822379 SRR29305284 Metagenomic study : Human gut
microbiota AMPLICON METAGENOMIC PCR PAIRED Illumina MiSeq 353591 212209183
SAMN41705610 PRJNA1095229
SRP512155 SRS21535697 SRX24822380 SRR29305283 Metagenomic study : Human gut
microbiota AMPLICON METAGENOMIC PCR PAIRED Illumina MiSeq 35519 21326280
SAMN41705611 PRJNA1095229
SRP512155 SRS21535698 SRX24822381 SRR29305282 Metagenomic study : Human gut
microbiota AMPLICON METAGENOMIC PCR PAIRED Illumina MiSeq 57439 34427454
SAMN41705612 PRJNA1095229
SRP512155 SRS21535699 SRX24822382 SRR29305281 Metagenomic study : Human gut
microbiota AMPLICON METAGENOMIC PCR PAIRED Illumina MiSeq 182614 109084573
SAMN41705613 PRJNA1095229
SRP512155 SRS21535700 SRX24822383 SRR29305280 Metagenomic study : Human gut
microbiota AMPLICON METAGENOMIC PCR PAIRED Illumina MiSeq 547958 328947933
SAMN41705614 PRJNA1095229
SRP512155 SRS21535701 SRX24822384 SRR29305279 Metagenomic study : Human gut
microbiota AMPLICON METAGENOMIC PCR PAIRED Illumina MiSeq 272289 163444659
SAMN41705615 PRJNA1095229
SRP512155 SRS21535702 SRX24822385 SRR29305278 Metagenomic study : Human gut
microbiota AMPLICON METAGENOMIC PCR PAIRED Illumina MiSeq 13055 7794253
SAMN41705616 PRJNA1095229
SRP512155 SRS21535703 SRX24822386 SRR29305277 Metagenomic study : Human gut
microbiota AMPLICON METAGENOMIC PCR PAIRED Illumina MiSeq 85610 51396533
SAMN41705617 PRJNA1095229
SRP512155 SRS21535704 SRX24822387 SRR29305276 Metagenomic study : Human gut
microbiota AMPLICON METAGENOMIC PCR PAIRED Illumina MiSeq 34009 20405198
SAMN41705618 PRJNA1095229
SRP512155 SRS21535705 SRX24822388 SRR29305275 Metagenomic study : Human gut
microbiota AMPLICON METAGENOMIC PCR PAIRED Illumina MiSeq 149044 89455913
SAMN41705619 PRJNA1095229
SRP512155 SRS21535706 SRX24822389 SRR29305274 Metagenomic study : Human gut
microbiota AMPLICON METAGENOMIC PCR PAIRED Illumina MiSeq 83241 49995725
SAMN41705566 PRJNA1095229
SRP512155 SRS21535707 SRX24822390 SRR29305273 Metagenomic study : Human gut
microbiota AMPLICON METAGENOMIC PCR PAIRED Illumina MiSeq 99520 59401170
SAMN41705620 PRJNA1095229
SRP512155 SRS21535708 SRX24822391 SRR29305272 Metagenomic study : Human gut
microbiota AMPLICON METAGENOMIC PCR PAIRED Illumina MiSeq 212049 127283603
SAMN41705621 PRJNA1095229
SRP512155 SRS21535709 SRX24822392 SRR29305271 Metagenomic study : Human gut
microbiota AMPLICON METAGENOMIC PCR PAIRED Illumina MiSeq 113688 68171236
SAMN41705622 PRJNA1095229
SRP512155 SRS21535710 SRX24822393 SRR29305270 Metagenomic study : Human gut
microbiota AMPLICON METAGENOMIC PCR PAIRED Illumina MiSeq 214975 128725235
SAMN41705623 PRJNA1095229
SRP512155 SRS21535711 SRX24822394 SRR29305269 Metagenomic study : Human gut
microbiota AMPLICON METAGENOMIC PCR PAIRED Illumina MiSeq 252975 151787099
SAMN41705624 PRJNA1095229
SRP512155 SRS21535712 SRX24822395 SRR29305268 Metagenomic study : Human gut
microbiota AMPLICON METAGENOMIC PCR PAIRED Illumina MiSeq 224425 134702674
SAMN41705625 PRJNA1095229
SRP512155 SRS21535713 SRX24822396 SRR29305267 Metagenomic study : Human gut
microbiota AMPLICON METAGENOMIC PCR PAIRED Illumina MiSeq 162977 97662498
SAMN41705626 PRJNA1095229
SRP512155 SRS21535714 SRX24822397 SRR29305266 Metagenomic study : Human gut
microbiota AMPLICON METAGENOMIC PCR PAIRED Illumina MiSeq 54108 32410354
SAMN41705627 PRJNA1095229
SRP512155 SRS21535715 SRX24822398 SRR29305265 Metagenomic study : Human gut
microbiota AMPLICON METAGENOMIC PCR PAIRED Illumina MiSeq 545176 326896666
SAMN41705628 PRJNA1095229
SRP512155 SRS21535716 SRX24822399 SRR29305264 Metagenomic study : Human gut
microbiota AMPLICON METAGENOMIC PCR PAIRED Illumina MiSeq 609638 363926572
SAMN41705629 PRJNA1095229

SRP512155	SRS21535717	SRX24822400	SRR29305263	Metagenomic study : Human gut microbiota	AMPLICON	METAGENOMIC	PCR	PAIRED	Illumina MiSeq	123942	74298708	SAMN41705567	PRJNA1095229
SRP512155	SRS21535718	SRX24822401	SRR29305262	Metagenomic study : Human gut microbiota	AMPLICON	METAGENOMIC	PCR	PAIRED	Illumina MiSeq	464777	278458224	SAMN41705630	PRJNA1095229
SRP512155	SRS21535719	SRX24822402	SRR29305261	Metagenomic study : Human gut microbiota	AMPLICON	METAGENOMIC	PCR	PAIRED	Illumina MiSeq	51041	30608230	SAMN41705631	PRJNA1095229
SRP512155	SRS21535720	SRX24822403	SRR29305260	Metagenomic study : Human gut microbiota	AMPLICON	METAGENOMIC	PCR	PAIRED	Illumina MiSeq	88589	53208556	SAMN41705632	PRJNA1095229
SRP512155	SRS21535721	SRX24822404	SRR29305259	Metagenomic study : Human gut microbiota	AMPLICON	METAGENOMIC	PCR	PAIRED	Illumina MiSeq	476193	285638397	SAMN41705633	PRJNA1095229
SRP512155	SRS21535722	SRX24822405	SRR29305258	Metagenomic study : Human gut microbiota	AMPLICON	METAGENOMIC	PCR	PAIRED	Illumina MiSeq	111138	66718051	SAMN41705634	PRJNA1095229
SRP512155	SRS21535723	SRX24822406	SRR29305257	Metagenomic study : Human gut microbiota	AMPLICON	METAGENOMIC	PCR	PAIRED	Illumina MiSeq	118873	71411002	SAMN41705635	PRJNA1095229
SRP512155	SRS21535724	SRX24822407	SRR29305256	Metagenomic study : Human gut microbiota	AMPLICON	METAGENOMIC	PCR	PAIRED	Illumina MiSeq	140680	84440564	SAMN41705636	PRJNA1095229
SRP512155	SRS21535725	SRX24822408	SRR29305255	Metagenomic study : Human gut microbiota	AMPLICON	METAGENOMIC	PCR	PAIRED	Illumina MiSeq	298260	178760520	SAMN41705637	PRJNA1095229
SRP512155	SRS21535726	SRX24822409	SRR29305254	Metagenomic study : Human gut microbiota	AMPLICON	METAGENOMIC	PCR	PAIRED	Illumina MiSeq	282487	169410833	SAMN41705638	PRJNA1095229
SRP512155	SRS21535727	SRX24822410	SRR29305253	Metagenomic study : Human gut microbiota	AMPLICON	METAGENOMIC	PCR	PAIRED	Illumina MiSeq	414154	248693888	SAMN41705639	PRJNA1095229
SRP512155	SRS21535728	SRX24822411	SRR29305252	Metagenomic study : Human gut microbiota	AMPLICON	METAGENOMIC	PCR	PAIRED	Illumina MiSeq	332754	199628814	SAMN41705568	PRJNA1095229
SRP512155	SRS21535729	SRX24822412	SRR29305251	Metagenomic study : Human gut microbiota	AMPLICON	METAGENOMIC	PCR	PAIRED	Illumina MiSeq	108824	65230570	SAMN41705640	PRJNA1095229
SRP512155	SRS21535730	SRX24822413	SRR29305250	Metagenomic study : Human gut microbiota	AMPLICON	METAGENOMIC	PCR	PAIRED	Illumina MiSeq	92839	55700522	SAMN41705641	PRJNA1095229
SRP512155	SRS21535731	SRX24822414	SRR29305249	Metagenomic study : Human gut microbiota	AMPLICON	METAGENOMIC	PCR	PAIRED	Illumina MiSeq	52134	31241594	SAMN41705642	PRJNA1095229
SRP512155	SRS21535732	SRX24822415	SRR29305248	Metagenomic study : Human gut microbiota	AMPLICON	METAGENOMIC	PCR	PAIRED	Illumina MiSeq	595005	357200630	SAMN41705643	PRJNA1095229
SRP512155	SRS21535733	SRX24822416	SRR29305247	Metagenomic study : Human gut microbiota	AMPLICON	METAGENOMIC	PCR	PAIRED	Illumina MiSeq	45110	27076091	SAMN41705644	PRJNA1095229
SRP512155	SRS21535734	SRX24822417	SRR29305246	Metagenomic study : Human gut microbiota	AMPLICON	METAGENOMIC	PCR	PAIRED	Illumina MiSeq	526281	314947645	SAMN41705645	PRJNA1095229
SRP512155	SRS21535735	SRX24822418	SRR29305245	Metagenomic study : Human gut microbiota	AMPLICON	METAGENOMIC	PCR	PAIRED	Illumina MiSeq	126546	76007283	SAMN41705646	PRJNA1095229

acc name organism tag value
SRS21535626 aUv2 gut metagenome host Homo sapiens
SRS21535626 aUv2 gut metagenome collection_date 2018
SRS21535626 aUv2 gut metagenome geo_loc_name Tunisia
SRS21535626 aUv2 gut metagenome lat_lon 33.886917 N 9.537499 E
SRS21535626 aUv2 gut metagenome samp_size 110
SRS21535626 aUv2 gut metagenome participants_features Adult
SRS21535626 aUv2 gut metagenome sex male
SRS21535626 aUv2 gut metagenome age 41
SRS21535626 aUv2 gut metagenome study_design Case-control
SRS21535626 aUv2 gut metagenome disease Non Diabetic
SRS21535626 aUv2 gut metagenome study_title Metataxonomic strategy to decipher gut bacteriome biomarkers of type 2 diabetes and obesity in Tunisian population
SRS21535626 aUv2 gut metagenome continent Africa
SRS21535626 aUv2 gut metagenome region North Africa
SRS21535626 aUv2 gut metagenome assay_type Amplicon
SRS21535626 aUv2 gut metagenome technology Illumina
SRS21535626 aUv2 gut metagenome platform Illumina MiSeq
SRS21535626 aUv2 gut metagenome average_spotlength 601
SRS21535626 aUv2 gut metagenome library_layout Paired
SRS21535626 aUv2 gut metagenome target_amplicon V3-V4
SRS21535626 aUv2 gut metagenome submitters Meriem FASSATOUI/Abdelmajid ABID/Rym KEFI
SRS21535626 aUv2 gut metagenome Isolate met
SRS21535626 aUv2 gut metagenome BioSampleModel Metagenome or environmental
SRS21535627 BD5A gut metagenome host Homo sapiens
SRS21535627 BD5A gut metagenome collection_date 2018
SRS21535627 BD5A gut metagenome geo_loc_name Tunisia
SRS21535627 BD5A gut metagenome lat_lon 33.886917 N 9.537499 E
SRS21535627 BD5A gut metagenome samp_size 110
SRS21535627 BD5A gut metagenome participants_features Adult
SRS21535627 BD5A gut metagenome sex female
SRS21535627 BD5A gut metagenome age 37
SRS21535627 BD5A gut metagenome study_design Case-control
SRS21535627 BD5A gut metagenome disease Non Diabetic
SRS21535627 BD5A gut metagenome study_title Metataxonomic strategy to decipher gut bacteriome biomarkers of type 2 diabetes and obesity in Tunisian population
SRS21535627 BD5A gut metagenome continent Africa
SRS21535627 BD5A gut metagenome region North Africa
SRS21535627 BD5A gut metagenome assay_type Amplicon
SRS21535627 BD5A gut metagenome technology Illumina
SRS21535627 BD5A gut metagenome platform Illumina MiSeq
SRS21535627 BD5A gut metagenome average_spotlength 601
SRS21535627 BD5A gut metagenome library_layout Paired
SRS21535627 BD5A gut metagenome target_amplicon V3-V4
SRS21535627 BD5A gut metagenome submitters Meriem FASSATOUI/Abdelmajid ABID/Rym KEFI
SRS21535627 BD5A gut metagenome Isolate for
SRS21535627 BD5A gut metagenome BioSampleModel Metagenome or environmental
SRS21535628 47LU gut metagenome host Homo sapiens
SRS21535628 47LU gut metagenome collection_date 2018
SRS21535628 47LU gut metagenome geo_loc_name Tunisia
SRS21535628 47LU gut metagenome lat_lon 33.886917 N 9.537499 E
SRS21535628 47LU gut metagenome samp_size 110
SRS21535628 47LU gut metagenome participants_features Adult
SRS21535628 47LU gut metagenome sex female
SRS21535628 47LU gut metagenome age 54
SRS21535628 47LU gut metagenome study_design Case-control
SRS21535628 47LU gut metagenome disease Non Diabetic
SRS21535628 47LU gut metagenome study_title Metataxonomic strategy to decipher gut bacteriome biomarkers of type 2 diabetes and obesity in Tunisian population
SRS21535628 47LU gut metagenome continent Africa
SRS21535628 47LU gut metagenome region North Africa
SRS21535628 47LU gut metagenome assay_type Amplicon
SRS21535628 47LU gut metagenome technology Illumina
SRS21535628 47LU gut metagenome platform Illumina MiSeq
SRS21535628 47LU gut metagenome average_spotlength 595
SRS21535628 47LU gut metagenome library_layout Paired
SRS21535628 47LU gut metagenome target_amplicon V3-V4
SRS21535628 47LU gut metagenome submitters Meriem FASSATOUI/Abdelmajid ABID/Rym KEFI
SRS21535628 47LU gut metagenome Isolate PRAL

SRS21535628 47LU gut metagenome BioSampleModel Metagenome or environmental
SRS21535629 HBC gut metagenome host Homo sapiens
SRS21535629 HBC gut metagenome collection_date 2018
SRS21535629 HBC gut metagenome geo_loc_name Tunisia
SRS21535629 HBC gut metagenome lat_lon 33.886917 N 9.537499 E
SRS21535629 HBC gut metagenome samp_size 110
SRS21535629 HBC gut metagenome participants_features Adult
SRS21535629 HBC gut metagenome sex female
SRS21535629 HBC gut metagenome age 57
SRS21535629 HBC gut metagenome study_design Case-control
SRS21535629 HBC gut metagenome disease Type 2 diabetic with obesity and cardiovascular complications
SRS21535629 HBC gut metagenome disease_ontology_id 9352
SRS21535629 HBC gut metagenome study_title Metataxonomic strategy to decipher gut bacteriome biomarkers of type 2 diabetes and obesity in Tunisian population
SRS21535629 HBC gut metagenome continent Africa
SRS21535629 HBC gut metagenome region North Africa
SRS21535629 HBC gut metagenome assay_type Amplicon
SRS21535629 HBC gut metagenome technology Illumina
SRS21535629 HBC gut metagenome platform Illumina MiSeq
SRS21535629 HBC gut metagenome average_spotlength 601
SRS21535629 HBC gut metagenome library_layout Paired
SRS21535629 HBC gut metagenome target_amplicon V3-V4
SRS21535629 HBC gut metagenome submitters Meriem FASSATOUI/Abdelmajid ABID/Rym KEFI
SRS21535629 HBC gut metagenome Isolate naoh
SRS21535629 HBC gut metagenome BioSampleModel Metagenome or environmental
SRS21535630 MONKa gut metagenome host Homo sapiens
SRS21535630 MONKa gut metagenome collection_date 2018
SRS21535630 MONKa gut metagenome geo_loc_name Tunisia
SRS21535630 MONKa gut metagenome lat_lon 33.886917 N 9.537499 E
SRS21535630 MONKa gut metagenome samp_size 110
SRS21535630 MONKa gut metagenome participants_features Adult
SRS21535630 MONKa gut metagenome sex female
SRS21535630 MONKa gut metagenome age 50
SRS21535630 MONKa gut metagenome study_design Case-control
SRS21535630 MONKa gut metagenome disease Type 2 diabetic with obesity and cardiovascular complications
SRS21535630 MONKa gut metagenome disease_ontology_id 9352
SRS21535630 MONKa gut metagenome study_title Metataxonomic strategy to decipher gut bacteriome biomarkers of type 2 diabetes and obesity in Tunisian population
SRS21535630 MONKa gut metagenome continent Africa
SRS21535630 MONKa gut metagenome region North Africa
SRS21535630 MONKa gut metagenome assay_type Amplicon
SRS21535630 MONKa gut metagenome technology Illumina
SRS21535630 MONKa gut metagenome platform Illumina MiSeq
SRS21535630 MONKa gut metagenome average_spotlength 601
SRS21535630 MONKa gut metagenome library_layout Paired
SRS21535630 MONKa gut metagenome target_amplicon V3-V4
SRS21535630 MONKa gut metagenome submitters Meriem FASSATOUI/Abdelmajid ABID/Rym KEFI
SRS21535630 MONKa gut metagenome Isolate clima
SRS21535630 MONKa gut metagenome BioSampleModel Metagenome or environmental
SRS21535631 HIVPOX gut metagenome host Homo sapiens
SRS21535631 HIVPOX gut metagenome collection_date 2018
SRS21535631 HIVPOX gut metagenome geo_loc_name Tunisia
SRS21535631 HIVPOX gut metagenome lat_lon 33.886917 N 9.537499 E
SRS21535631 HIVPOX gut metagenome samp_size 110
SRS21535631 HIVPOX gut metagenome participants_features Adult
SRS21535631 HIVPOX gut metagenome sex female
SRS21535631 HIVPOX gut metagenome age 61
SRS21535631 HIVPOX gut metagenome study_design Case-control
SRS21535631 HIVPOX gut metagenome disease Type 2 diabetic with obesity and cardiovascular complications
SRS21535631 HIVPOX gut metagenome disease_ontology_id 9352
SRS21535631 HIVPOX gut metagenome study_title Metataxonomic strategy to decipher gut bacteriome biomarkers of type 2 diabetes and obesity in Tunisian population
SRS21535631 HIVPOX gut metagenome continent Africa
SRS21535631 HIVPOX gut metagenome region North Africa
SRS21535631 HIVPOX gut metagenome assay_type Amplicon
SRS21535631 HIVPOX gut metagenome technology Illumina

SRS21535631 HIVPOX gut metagenome platform Illumina MiSeq
SRS21535631 HIVPOX gut metagenome average_spotlength 601
SRS21535631 HIVPOX gut metagenome library_layout Paired
SRS21535631 HIVPOX gut metagenome target_amplicon V3-V4
SRS21535631 HIVPOX gut metagenome submitters Meriem FASSATOUI/Abdelmajid ABID/Rym KEFI
SRS21535631 HIVPOX gut metagenome Isolate carbon
SRS21535631 HIVPOX gut metagenome BioSampleModel Metagenome or environmental
SRS21535632 HTLV gut metagenome host Homo sapiens
SRS21535632 HTLV gut metagenome collection_date 2018
SRS21535632 HTLV gut metagenome geo_loc_name Tunisia
SRS21535632 HTLV gut metagenome lat_lon 33.886917 N 9.537499 E
SRS21535632 HTLV gut metagenome samp_size 110
SRS21535632 HTLV gut metagenome participants_features Adult
SRS21535632 HTLV gut metagenome sex female
SRS21535632 HTLV gut metagenome age 50
SRS21535632 HTLV gut metagenome study_design Case-control
SRS21535632 HTLV gut metagenome disease Type 2 diabetic with obesity and cardiovascular complications
SRS21535632 HTLV gut metagenome disease_ontology_id 9352
SRS21535632 HTLV gut metagenome study_title Metataxonomic strategy to decipher gut bacteriome biomarkers of type 2 diabetes and obesity in Tunisian population
SRS21535632 HTLV gut metagenome continent Africa
SRS21535632 HTLV gut metagenome region North Africa
SRS21535632 HTLV gut metagenome assay_type Amplicon
SRS21535632 HTLV gut metagenome technology Illumina
SRS21535632 HTLV gut metagenome platform Illumina MiSeq
SRS21535632 HTLV gut metagenome average_spotlength 601
SRS21535632 HTLV gut metagenome library_layout Paired
SRS21535632 HTLV gut metagenome target_amplicon V3-V4
SRS21535632 HTLV gut metagenome submitters Meriem FASSATOUI/Abdelmajid ABID/Rym KEFI
SRS21535632 HTLV gut metagenome Isolate jewe
SRS21535632 HTLV gut metagenome BioSampleModel Metagenome or environmental
SRS21535633 HIGHli gut metagenome host Homo sapiens
SRS21535633 HIGHli gut metagenome collection_date 2018
SRS21535633 HIGHli gut metagenome geo_loc_name Tunisia
SRS21535633 HIGHli gut metagenome lat_lon 33.886917 N 9.537499 E
SRS21535633 HIGHli gut metagenome samp_size 110
SRS21535633 HIGHli gut metagenome participants_features Adult
SRS21535633 HIGHli gut metagenome sex female
SRS21535633 HIGHli gut metagenome age 51
SRS21535633 HIGHli gut metagenome study_design Case-control
SRS21535633 HIGHli gut metagenome disease Type 2 diabetic with obesity and cardiovascular complications
SRS21535633 HIGHli gut metagenome disease_ontology_id 9352
SRS21535633 HIGHli gut metagenome study_title Metataxonomic strategy to decipher gut bacteriome biomarkers of type 2 diabetes and obesity in Tunisian population
SRS21535633 HIGHli gut metagenome continent Africa
SRS21535633 HIGHli gut metagenome region North Africa
SRS21535633 HIGHli gut metagenome assay_type Amplicon
SRS21535633 HIGHli gut metagenome technology Illumina
SRS21535633 HIGHli gut metagenome platform Illumina MiSeq
SRS21535633 HIGHli gut metagenome average_spotlength 601
SRS21535633 HIGHli gut metagenome library_layout Paired
SRS21535633 HIGHli gut metagenome target_amplicon V3-V4
SRS21535633 HIGHli gut metagenome submitters Meriem FASSATOUI/Abdelmajid ABID/Rym KEFI
SRS21535633 HIGHli gut metagenome Isolate circos
SRS21535633 HIGHli gut metagenome BioSampleModel Metagenome or environmental
SRS21535634 VPH gut metagenome host Homo sapiens
SRS21535634 VPH gut metagenome collection_date 2018
SRS21535634 VPH gut metagenome geo_loc_name Tunisia
SRS21535634 VPH gut metagenome lat_lon 33.886917 N 9.537499 E
SRS21535634 VPH gut metagenome samp_size 110
SRS21535634 VPH gut metagenome participants_features Adult
SRS21535634 VPH gut metagenome sex female
SRS21535634 VPH gut metagenome age 52
SRS21535634 VPH gut metagenome study_design Case-control
SRS21535634 VPH gut metagenome disease Type 2 diabetic with obesity and cardiovascular complications
SRS21535634 VPH gut metagenome disease_ontology_id 9352

SRS21535634 VPH gut metagenome study_title Metataxonomic strategy to decipher gut bacteriome biomarkers of type 2 diabetes and obesity in Tunisian population
SRS21535634 VPH gut metagenome continent Africa
SRS21535634 VPH gut metagenome region North Africa
SRS21535634 VPH gut metagenome assay_type Amplicon
SRS21535634 VPH gut metagenome technology Illumina
SRS21535634 VPH gut metagenome platform Illumina MiSeq
SRS21535634 VPH gut metagenome average_spotlength 601
SRS21535634 VPH gut metagenome library_layout Paired
SRS21535634 VPH gut metagenome target_amplicon V3-V4
SRS21535634 VPH gut metagenome submitters Meriem FASSATOUI/Abdelmajid ABID/Rym KEFI
SRS21535634 VPH gut metagenome Isolate mutag
SRS21535634 VPH gut metagenome BioSampleModel Metagenome or environmental
SRS21535635 LOM gut metagenome host Homo sapiens
SRS21535635 LOM gut metagenome collection_date 2018
SRS21535635 LOM gut metagenome geo_loc_name Tunisia
SRS21535635 LOM gut metagenome lat_lon 33.886917 N 9.537499 E
SRS21535635 LOM gut metagenome samp_size 110
SRS21535635 LOM gut metagenome participants_features Adult
SRS21535635 LOM gut metagenome sex male
SRS21535635 LOM gut metagenome age 59
SRS21535635 LOM gut metagenome study_design Case-control
SRS21535635 LOM gut metagenome disease Type 2 diabetic with obesity and cardiovascular complications
SRS21535635 LOM gut metagenome disease_ontology_id 9352
SRS21535635 LOM gut metagenome study_title Metataxonomic strategy to decipher gut bacteriome biomarkers of type 2 diabetes and obesity in Tunisian population
SRS21535635 LOM gut metagenome continent Africa
SRS21535635 LOM gut metagenome region North Africa
SRS21535635 LOM gut metagenome assay_type Amplicon
SRS21535635 LOM gut metagenome technology Illumina
SRS21535635 LOM gut metagenome platform Illumina MiSeq
SRS21535635 LOM gut metagenome average_spotlength 601
SRS21535635 LOM gut metagenome library_layout Paired
SRS21535635 LOM gut metagenome target_amplicon V3-V4
SRS21535635 LOM gut metagenome submitters Meriem FASSATOUI/Abdelmajid ABID/Rym KEFI
SRS21535635 LOM gut metagenome Isolate index
SRS21535635 LOM gut metagenome BioSampleModel Metagenome or environmental
SRS21535636 SEURA gut metagenome host Homo sapiens
SRS21535636 SEURA gut metagenome collection_date 2018
SRS21535636 SEURA gut metagenome geo_loc_name Tunisia
SRS21535636 SEURA gut metagenome lat_lon 33.886917 N 9.537499 E
SRS21535636 SEURA gut metagenome samp_size 110
SRS21535636 SEURA gut metagenome participants_features Adult
SRS21535636 SEURA gut metagenome sex male
SRS21535636 SEURA gut metagenome age 56
SRS21535636 SEURA gut metagenome study_design Case-control
SRS21535636 SEURA gut metagenome disease Type 2 diabetic with obesity and cardiovascular complications
SRS21535636 SEURA gut metagenome disease_ontology_id 9352
SRS21535636 SEURA gut metagenome study_title Metataxonomic strategy to decipher gut bacteriome biomarkers of type 2 diabetes and obesity in Tunisian population
SRS21535636 SEURA gut metagenome continent Africa
SRS21535636 SEURA gut metagenome region North Africa
SRS21535636 SEURA gut metagenome assay_type Amplicon
SRS21535636 SEURA gut metagenome technology Illumina
SRS21535636 SEURA gut metagenome platform Illumina MiSeq
SRS21535636 SEURA gut metagenome average_spotlength 601
SRS21535636 SEURA gut metagenome library_layout Paired
SRS21535636 SEURA gut metagenome target_amplicon V3-V4
SRS21535636 SEURA gut metagenome submitters Meriem FASSATOUI/Abdelmajid ABID/Rym KEFI
SRS21535636 SEURA gut metagenome Isolate lab
SRS21535636 SEURA gut metagenome BioSampleModel Metagenome or environmental
SRS21535637 NAIP gut metagenome host Homo sapiens
SRS21535637 NAIP gut metagenome collection_date 2018
SRS21535637 NAIP gut metagenome geo_loc_name Tunisia
SRS21535637 NAIP gut metagenome lat_lon 33.886917 N 9.537499 E
SRS21535637 NAIP gut metagenome samp_size 110
SRS21535637 NAIP gut metagenome participants_features Adult

```
SRS21535637	NAIP	gut metagenome	sex	male
SRS21535637	NAIP	gut metagenome	age	73
SRS21535637	NAIP	gut metagenome	study_design	Case-control
SRS21535637	NAIP	gut metagenome	disease	Type 2 diabetic with obesity and cardiovascular complications
SRS21535637	NAIP	gut metagenome	disease_ontology_id	9352
SRS21535637	NAIP	gut metagenome	study_title	Metataxonomic strategy to decipher gut bacteriome biomarkers of type 2 diabetes and obesity in Tunisian population
SRS21535637	NAIP	gut metagenome	continent	Africa
SRS21535637	NAIP	gut metagenome	region	North Africa
SRS21535637	NAIP	gut metagenome	assay_type	Amplicon
SRS21535637	NAIP	gut metagenome	technology	Illumina
SRS21535637	NAIP	gut metagenome	platform	Illumina MiSeq
SRS21535637	NAIP	gut metagenome	average_spotlength	601
SRS21535637	NAIP	gut metagenome	library_layout	Paired
SRS21535637	NAIP	gut metagenome	target_amplicon	V3-V4
SRS21535637	NAIP	gut metagenome	submitters	Meriem FASSATOUI/Abdelmajid ABID/Rym KEFI
SRS21535637	NAIP	gut metagenome	Isolate	bind
SRS21535637	NAIP	gut metagenome	BioSampleModel	Metagenome or environmental
SRS21535638	NEARt	gut metagenome	host	Homo sapiens
SRS21535638	NEARt	gut metagenome	collection_date	2018
SRS21535638	NEARt	gut metagenome	geo_loc_name	Tunisia
SRS21535638	NEARt	gut metagenome	lat_lon	33.886917 N 9.537499 E
SRS21535638	NEARt	gut metagenome	samp_size	110
SRS21535638	NEARt	gut metagenome	participants_features	Adult
SRS21535638	NEARt	gut metagenome	sex	male
SRS21535638	NEARt	gut metagenome	age	55
SRS21535638	NEARt	gut metagenome	study_design	Case-control
SRS21535638	NEARt	gut metagenome	disease	Type 2 diabetic with obesity and cardiovascular complications
SRS21535638	NEARt	gut metagenome	disease_ontology_id	9352
SRS21535638	NEARt	gut metagenome	study_title	Metataxonomic strategy to decipher gut bacteriome biomarkers of type 2 diabetes and obesity in Tunisian population
SRS21535638	NEARt	gut metagenome	continent	Africa
SRS21535638	NEARt	gut metagenome	region	North Africa
SRS21535638	NEARt	gut metagenome	assay_type	Amplicon
SRS21535638	NEARt	gut metagenome	technology	Illumina
SRS21535638	NEARt	gut metagenome	platform	Illumina MiSeq
SRS21535638	NEARt	gut metagenome	average_spotlength	601
SRS21535638	NEARt	gut metagenome	library_layout	Paired
SRS21535638	NEARt	gut metagenome	target_amplicon	V3-V4
SRS21535638	NEARt	gut metagenome	submitters	Meriem FASSATOUI/Abdelmajid ABID/Rym KEFI
SRS21535638	NEARt	gut metagenome	Isolate	art
SRS21535638	NEARt	gut metagenome	BioSampleModel	Metagenome or environmental
SRS21535639	DIN8	gut metagenome	host	Homo sapiens
SRS21535639	DIN8	gut metagenome	collection_date	2018
SRS21535639	DIN8	gut metagenome	geo_loc_name	Tunisia
SRS21535639	DIN8	gut metagenome	lat_lon	33.886917 N 9.537499 E
SRS21535639	DIN8	gut metagenome	samp_size	110
SRS21535639	DIN8	gut metagenome	participants_features	Adult
SRS21535639	DIN8	gut metagenome	sex	female
SRS21535639	DIN8	gut metagenome	age	31
SRS21535639	DIN8	gut metagenome	study_design	Case-control
SRS21535639	DIN8	gut metagenome	disease	Non Diabetic
SRS21535639	DIN8	gut metagenome	study_title	Metataxonomic strategy to decipher gut bacteriome biomarkers of type 2 diabetes and obesity in Tunisian population
SRS21535639	DIN8	gut metagenome	continent	Africa
SRS21535639	DIN8	gut metagenome	region	North Africa
SRS21535639	DIN8	gut metagenome	assay_type	Amplicon
SRS21535639	DIN8	gut metagenome	technology	Illumina
SRS21535639	DIN8	gut metagenome	platform	Illumina MiSeq
SRS21535639	DIN8	gut metagenome	average_spotlength	601
SRS21535639	DIN8	gut metagenome	library_layout	Paired
SRS21535639	DIN8	gut metagenome	target_amplicon	V3-V4
SRS21535639	DIN8	gut metagenome	submitters	Meriem FASSATOUI/Abdelmajid ABID/Rym KEFI
SRS21535639	DIN8	gut metagenome	Isolate	GAV
SRS21535639	DIN8	gut metagenome	BioSampleModel	Metagenome or environmental
SRS21535640	freAHS	gut metagenome	host	Homo sapiens
SRS21535640	freAHS	gut metagenome	collection_date	2018
```

```
SRS21535640	freAHS	gut metagenome	geo_loc_name	Tunisia
SRS21535640	freAHS	gut metagenome	lat_lon	33.886917 N 9.537499 E
SRS21535640	freAHS	gut metagenome	samp_size	110
SRS21535640	freAHS	gut metagenome	participants_features	Adult
SRS21535640	freAHS	gut metagenome	sex	male
SRS21535640	freAHS	gut metagenome	age	64
SRS21535640	freAHS	gut metagenome	study_design	Case-control
SRS21535640	freAHS	gut metagenome	disease	Non Diabetic
SRS21535640	freAHS	gut metagenome	study_title	Metataxonomic strategy to decipher gut
bacteriome biomarkers of type 2 diabetes and obesity in Tunisian population
SRS21535640	freAHS	gut metagenome	continent	Africa
SRS21535640	freAHS	gut metagenome	region	North Africa
SRS21535640	freAHS	gut metagenome	assay_type	Amplicon
SRS21535640	freAHS	gut metagenome	technology	Illumina
SRS21535640	freAHS	gut metagenome	platform	Illumina MiSeq
SRS21535640	freAHS	gut metagenome	average_spotlength	601
SRS21535640	freAHS	gut metagenome	library_layout	Paired
SRS21535640	freAHS	gut metagenome	target_amplicon	V3-V4
SRS21535640	freAHS	gut metagenome	submitters	Meriem FASSATOUI/Abdelmajid ABID/Rym KEFI
SRS21535640	freAHS	gut metagenome	Isolate	ISCON
SRS21535640	freAHS	gut metagenome	BioSampleModel	Metagenome or environmental
SRS21535641	N52PZSW	gut metagenome	host	Homo sapiens
SRS21535641	N52PZSW	gut metagenome	collection_date	2018
SRS21535641	N52PZSW	gut metagenome	geo_loc_name	Tunisia
SRS21535641	N52PZSW	gut metagenome	lat_lon	33.886917 N 9.537499 E
SRS21535641	N52PZSW	gut metagenome	samp_size	110
SRS21535641	N52PZSW	gut metagenome	participants_features	Adult
SRS21535641	N52PZSW	gut metagenome	sex	male
SRS21535641	N52PZSW	gut metagenome	age	57
SRS21535641	N52PZSW	gut metagenome	study_design	Case-control
SRS21535641	N52PZSW	gut metagenome	disease	Non Diabetic
SRS21535641	N52PZSW	gut metagenome	study_title	Metataxonomic strategy to decipher gut
bacteriome biomarkers of type 2 diabetes and obesity in Tunisian population
SRS21535641	N52PZSW	gut metagenome	continent	Africa
SRS21535641	N52PZSW	gut metagenome	region	North Africa
SRS21535641	N52PZSW	gut metagenome	assay_type	Amplicon
SRS21535641	N52PZSW	gut metagenome	technology	Illumina
SRS21535641	N52PZSW	gut metagenome	platform	Illumina MiSeq
SRS21535641	N52PZSW	gut metagenome	average_spotlength	595
SRS21535641	N52PZSW	gut metagenome	library_layout	Paired
SRS21535641	N52PZSW	gut metagenome	target_amplicon	V3-V4
SRS21535641	N52PZSW	gut metagenome	submitters	Meriem FASSATOUI/Abdelmajid ABID/Rym KEFI
SRS21535641	N52PZSW	gut metagenome	Isolate	AER
SRS21535641	N52PZSW	gut metagenome	BioSampleModel	Metagenome or environmental
SRS21535642	54DON	gut metagenome	host	Homo sapiens
SRS21535642	54DON	gut metagenome	collection_date	2018
SRS21535642	54DON	gut metagenome	geo_loc_name	Tunisia
SRS21535642	54DON	gut metagenome	lat_lon	33.886917 N 9.537499 E
SRS21535642	54DON	gut metagenome	samp_size	110
SRS21535642	54DON	gut metagenome	participants_features	Adult
SRS21535642	54DON	gut metagenome	sex	female
SRS21535642	54DON	gut metagenome	age	36
SRS21535642	54DON	gut metagenome	study_design	Case-control
SRS21535642	54DON	gut metagenome	disease	Non Diabetic
SRS21535642	54DON	gut metagenome	study_title	Metataxonomic strategy to decipher gut
bacteriome biomarkers of type 2 diabetes and obesity in Tunisian population
SRS21535642	54DON	gut metagenome	continent	Africa
SRS21535642	54DON	gut metagenome	region	North Africa
SRS21535642	54DON	gut metagenome	assay_type	Amplicon
SRS21535642	54DON	gut metagenome	technology	Illumina
SRS21535642	54DON	gut metagenome	platform	Illumina MiSeq
SRS21535642	54DON	gut metagenome	average_spotlength	601
SRS21535642	54DON	gut metagenome	library_layout	Paired
SRS21535642	54DON	gut metagenome	target_amplicon	V3-V4
SRS21535642	54DON	gut metagenome	submitters	Meriem FASSATOUI/Abdelmajid ABID/Rym KEFI
SRS21535642	54DON	gut metagenome	Isolate	OL
SRS21535642	54DON	gut metagenome	BioSampleModel	Metagenome or environmental
SRS21535643	fxwq	gut metagenome	host	Homo sapiens
SRS21535643	fxwq	gut metagenome	collection_date	2018
```

```
SRS21535643	fxwq	gut metagenome	geo_loc_name	Tunisia
SRS21535643	fxwq	gut metagenome	lat_lon	33.886917 N 9.537499 E
SRS21535643	fxwq	gut metagenome	samp_size	110
SRS21535643	fxwq	gut metagenome	participants_features	Adult
SRS21535643	fxwq	gut metagenome	sex	female
SRS21535643	fxwq	gut metagenome	age	49
SRS21535643	fxwq	gut metagenome	study_design	Case-control
SRS21535643	fxwq	gut metagenome	disease	Non Diabetic
SRS21535643	fxwq	gut metagenome	study_title	Metataxonomic strategy to decipher gut
bacteriome biomarkers of type 2 diabetes and obesity in Tunisian population
SRS21535643	fxwq	gut metagenome	continent	Africa
SRS21535643	fxwq	gut metagenome	region	North Africa
SRS21535643	fxwq	gut metagenome	assay_type	Amplicon
SRS21535643	fxwq	gut metagenome	technology	Illumina
SRS21535643	fxwq	gut metagenome	platform	Illumina MiSeq
SRS21535643	fxwq	gut metagenome	average_spotlength	601
SRS21535643	fxwq	gut metagenome	library_layout	Paired
SRS21535643	fxwq	gut metagenome	target_amplicon	V3-V4
SRS21535643	fxwq	gut metagenome	submitters	Meriem FASSATOUI/Abdelmajid ABID/Rym KEFI
SRS21535643	fxwq	gut metagenome	Isolate	PHYSIO
SRS21535643	fxwq	gut metagenome	BioSampleModel	Metagenome or environmental
SRS21535644	pXEN	gut metagenome	host	Homo sapiens
SRS21535644	pXEN	gut metagenome	collection_date	2018
SRS21535644	pXEN	gut metagenome	geo_loc_name	Tunisia
SRS21535644	pXEN	gut metagenome	lat_lon	33.886917 N 9.537499 E
SRS21535644	pXEN	gut metagenome	samp_size	110
SRS21535644	pXEN	gut metagenome	participants_features	Adult
SRS21535644	pXEN	gut metagenome	sex	female
SRS21535644	pXEN	gut metagenome	age	47
SRS21535644	pXEN	gut metagenome	study_design	Case-control
SRS21535644	pXEN	gut metagenome	disease	Non Diabetic
SRS21535644	pXEN	gut metagenome	study_title	Metataxonomic strategy to decipher gut
bacteriome biomarkers of type 2 diabetes and obesity in Tunisian population
SRS21535644	pXEN	gut metagenome	continent	Africa
SRS21535644	pXEN	gut metagenome	region	North Africa
SRS21535644	pXEN	gut metagenome	assay_type	Amplicon
SRS21535644	pXEN	gut metagenome	technology	Illumina
SRS21535644	pXEN	gut metagenome	platform	Illumina MiSeq
SRS21535644	pXEN	gut metagenome	average_spotlength	601
SRS21535644	pXEN	gut metagenome	library_layout	Paired
SRS21535644	pXEN	gut metagenome	target_amplicon	V3-V4
SRS21535644	pXEN	gut metagenome	submitters	Meriem FASSATOUI/Abdelmajid ABID/Rym KEFI
SRS21535644	pXEN	gut metagenome	Isolate	MERT
SRS21535644	pXEN	gut metagenome	BioSampleModel	Metagenome or environmental
SRS21535645	Rpfd	gut metagenome	host	Homo sapiens
SRS21535645	Rpfd	gut metagenome	collection_date	2018
SRS21535645	Rpfd	gut metagenome	geo_loc_name	Tunisia
SRS21535645	Rpfd	gut metagenome	lat_lon	33.886917 N 9.537499 E
SRS21535645	Rpfd	gut metagenome	samp_size	110
SRS21535645	Rpfd	gut metagenome	participants_features	Adult
SRS21535645	Rpfd	gut metagenome	sex	female
SRS21535645	Rpfd	gut metagenome	age	56
SRS21535645	Rpfd	gut metagenome	study_design	Case-control
SRS21535645	Rpfd	gut metagenome	disease	Non Diabetic
SRS21535645	Rpfd	gut metagenome	study_title	Metataxonomic strategy to decipher gut
bacteriome biomarkers of type 2 diabetes and obesity in Tunisian population
SRS21535645	Rpfd	gut metagenome	continent	Africa
SRS21535645	Rpfd	gut metagenome	region	North Africa
SRS21535645	Rpfd	gut metagenome	assay_type	Amplicon
SRS21535645	Rpfd	gut metagenome	technology	Illumina
SRS21535645	Rpfd	gut metagenome	platform	Illumina MiSeq
SRS21535645	Rpfd	gut metagenome	average_spotlength	601
SRS21535645	Rpfd	gut metagenome	library_layout	Paired
SRS21535645	Rpfd	gut metagenome	target_amplicon	V3-V4
SRS21535645	Rpfd	gut metagenome	submitters	Meriem FASSATOUI/Abdelmajid ABID/Rym KEFI
SRS21535645	Rpfd	gut metagenome	Isolate	ACY
SRS21535645	Rpfd	gut metagenome	BioSampleModel	Metagenome or environmental
SRS21535646	nORW	gut metagenome	host	Homo sapiens
SRS21535646	nORW	gut metagenome	collection_date	2018
```

SRS21535646 nORW gut metagenome geo_loc_name Tunisia
SRS21535646 nORW gut metagenome lat_lon 33.886917 N 9.537499 E
SRS21535646 nORW gut metagenome samp_size 110
SRS21535646 nORW gut metagenome participants_features Adult
SRS21535646 nORW gut metagenome sex female
SRS21535646 nORW gut metagenome age 45
SRS21535646 nORW gut metagenome study_design Case-control
SRS21535646 nORW gut metagenome disease Non Diabetic
SRS21535646 nORW gut metagenome study_title Metataxonomic strategy to decipher gut bacteriome biomarkers of type 2 diabetes and obesity in Tunisian population
SRS21535646 nORW gut metagenome continent Africa
SRS21535646 nORW gut metagenome region North Africa
SRS21535646 nORW gut metagenome assay_type Amplicon
SRS21535646 nORW gut metagenome technology Illumina
SRS21535646 nORW gut metagenome platform Illumina MiSeq
SRS21535646 nORW gut metagenome average_spotlength 601
SRS21535646 nORW gut metagenome library_layout Paired
SRS21535646 nORW gut metagenome target_amplicon V3-V4
SRS21535646 nORW gut metagenome submitters Meriem FASSATOUI/Abdelmajid ABID/Rym KEFI
SRS21535646 nORW gut metagenome Isolate CLO
SRS21535646 nORW gut metagenome BioSampleModel Metagenome or environmental
SRS21535647 TGM gut metagenome host Homo sapiens
SRS21535647 TGM gut metagenome collection_date 2018
SRS21535647 TGM gut metagenome geo_loc_name Tunisia
SRS21535647 TGM gut metagenome lat_lon 33.886917 N 9.537499 E
SRS21535647 TGM gut metagenome samp_size 110
SRS21535647 TGM gut metagenome participants_features Adult
SRS21535647 TGM gut metagenome sex male
SRS21535647 TGM gut metagenome age 42
SRS21535647 TGM gut metagenome study_design Case-control
SRS21535647 TGM gut metagenome disease Non Diabetic
SRS21535647 TGM gut metagenome study_title Metataxonomic strategy to decipher gut bacteriome biomarkers of type 2 diabetes and obesity in Tunisian population
SRS21535647 TGM gut metagenome continent Africa
SRS21535647 TGM gut metagenome region North Africa
SRS21535712 YGRAM gut metagenome geo_loc_name Tunisia
SRS21535712 YGRAM gut metagenome lat_lon 33.886917 N 9.537499 E
SRS21535712 YGRAM gut metagenome samp_size 110
SRS21535712 YGRAM gut metagenome participants_features Adult
SRS21535712 YGRAM gut metagenome sex male
SRS21535712 YGRAM gut metagenome age 54
SRS21535712 YGRAM gut metagenome study_design Case-control
SRS21535712 YGRAM gut metagenome disease Type 2 diabetic
SRS21535712 YGRAM gut metagenome disease_ontology_id 9352
SRS21535712 YGRAM gut metagenome study_title Metataxonomic strategy to decipher gut bacteriome biomarkers of type 2 diabetes and obesity in Tunisian population
SRS21535712 YGRAM gut metagenome continent Africa
SRS21535712 YGRAM gut metagenome region North Africa
SRS21535712 YGRAM gut metagenome assay_type Amplicon
SRS21535712 YGRAM gut metagenome technology Illumina
SRS21535712 YGRAM gut metagenome platform Illumina MiSeq
SRS21535712 YGRAM gut metagenome average_spotlength 601
SRS21535712 YGRAM gut metagenome library_layout Paired
SRS21535712 YGRAM gut metagenome target_amplicon V3-V4
SRS21535712 YGRAM gut metagenome submitters Meriem FASSATOUI/Abdelmajid ABID/Rym KEFI
SRS21535712 YGRAM gut metagenome Isolate AJK
SRS21535712 YGRAM gut metagenome BioSampleModel Metagenome or environmental
SRS21535713 PYZAPI gut metagenome host Homo sapiens
SRS21535713 PYZAPI gut metagenome collection_date 2018
SRS21535713 PYZAPI gut metagenome geo_loc_name Tunisia
SRS21535713 PYZAPI gut metagenome lat_lon 33.886917 N 9.537499 E
SRS21535713 PYZAPI gut metagenome samp_size 110
SRS21535713 PYZAPI gut metagenome participants_features Adult
SRS21535713 PYZAPI gut metagenome sex male
SRS21535713 PYZAPI gut metagenome age 53
SRS21535713 PYZAPI gut metagenome study_design Case-control
SRS21535713 PYZAPI gut metagenome disease Type 2 diabetic
SRS21535713 PYZAPI gut metagenome disease_ontology_id 9352
SRS21535713 PYZAPI gut metagenome study_title Metataxonomic strategy to decipher gut

bacteriome biomarkers of type 2 diabetes and obesity in Tunisian population
SRS21535713 PYZAPI gut metagenome continent Africa
SRS21535713 PYZAPI gut metagenome region North Africa
SRS21535713 PYZAPI gut metagenome assay_type Amplicon
SRS21535713 PYZAPI gut metagenome technology Illumina
SRS21535713 PYZAPI gut metagenome platform Illumina MiSeq
SRS21535713 PYZAPI gut metagenome average_spotlength 601
SRS21535713 PYZAPI gut metagenome library_layout Paired
SRS21535713 PYZAPI gut metagenome target_amplicon V3-V4
SRS21535713 PYZAPI gut metagenome submitters Meriem FASSATOUI/Abdelmajid ABID/Rym KEFI
SRS21535713 PYZAPI gut metagenome Isolate GAT
SRS21535713 PYZAPI gut metagenome BioSampleModel Metagenome or environmental
SRS21535714 UTICAQUE gut metagenome host Homo sapiens
SRS21535714 UTICAQUE gut metagenome collection_date 2018
SRS21535714 UTICAQUE gut metagenome geo_loc_name Tunisia
SRS21535714 UTICAQUE gut metagenome lat_lon 33.886917 N 9.537499 E
SRS21535714 UTICAQUE gut metagenome samp_size 110
SRS21535714 UTICAQUE gut metagenome participants_features Adult
SRS21535714 UTICAQUE gut metagenome sex male
SRS21535714 UTICAQUE gut metagenome age 60
SRS21535714 UTICAQUE gut metagenome study_design Case-control
SRS21535714 UTICAQUE gut metagenome disease Type 2 diabetic
SRS21535714 UTICAQUE gut metagenome disease_ontology_id 9352
SRS21535714 UTICAQUE gut metagenome study_title Metataxonomic strategy to decipher gut bacteriome biomarkers of type 2 diabetes and obesity in Tunisian population
SRS21535714 UTICAQUE gut metagenome continent Africa
SRS21535714 UTICAQUE gut metagenome region North Africa
SRS21535714 UTICAQUE gut metagenome assay_type Amplicon
SRS21535714 UTICAQUE gut metagenome technology Illumina
SRS21535714 UTICAQUE gut metagenome platform Illumina MiSeq
SRS21535714 UTICAQUE gut metagenome average_spotlength 601
SRS21535714 UTICAQUE gut metagenome library_layout Paired
SRS21535714 UTICAQUE gut metagenome target_amplicon V3-V4
SRS21535714 UTICAQUE gut metagenome submitters Meriem FASSATOUI/Abdelmajid ABID/Rym KEFI
SRS21535714 UTICAQUE gut metagenome Isolate STAR
SRS21535714 UTICAQUE gut metagenome BioSampleModel Metagenome or environmental
SRS21535715 YICHANGF gut metagenome host Homo sapiens
SRS21535715 YICHANGF gut metagenome collection_date 2018
SRS21535715 YICHANGF gut metagenome geo_loc_name Tunisia
SRS21535715 YICHANGF gut metagenome lat_lon 33.886917 N 9.537499 E
SRS21535715 YICHANGF gut metagenome samp_size 110
SRS21535715 YICHANGF gut metagenome participants_features Adult
SRS21535715 YICHANGF gut metagenome sex female
SRS21535715 YICHANGF gut metagenome age 56
SRS21535715 YICHANGF gut metagenome study_design Case-control
SRS21535715 YICHANGF gut metagenome disease Type 2 diabetic
SRS21535715 YICHANGF gut metagenome disease_ontology_id 9352
SRS21535715 YICHANGF gut metagenome study_title Metataxonomic strategy to decipher gut bacteriome biomarkers of type 2 diabetes and obesity in Tunisian population
SRS21535715 YICHANGF gut metagenome continent Africa
SRS21535715 YICHANGF gut metagenome region North Africa
SRS21535715 YICHANGF gut metagenome assay_type Amplicon
SRS21535715 YICHANGF gut metagenome technology Illumina
SRS21535715 YICHANGF gut metagenome platform Illumina MiSeq
SRS21535715 YICHANGF gut metagenome average_spotlength 601
SRS21535715 YICHANGF gut metagenome library_layout Paired
SRS21535715 YICHANGF gut metagenome target_amplicon V3-V4
SRS21535715 YICHANGF gut metagenome submitters Meriem FASSATOUI/Abdelmajid ABID/Rym KEFI
SRS21535715 YICHANGF gut metagenome Isolate FILM
SRS21535715 YICHANGF gut metagenome BioSampleModel Metagenome or environmental
SRS21535716 pyraSARS gut metagenome host Homo sapiens
SRS21535716 pyraSARS gut metagenome collection_date 2018
SRS21535716 pyraSARS gut metagenome geo_loc_name Tunisia
SRS21535716 pyraSARS gut metagenome lat_lon 33.886917 N 9.537499 E
SRS21535716 pyraSARS gut metagenome samp_size 110
SRS21535716 pyraSARS gut metagenome participants_features Adult
SRS21535716 pyraSARS gut metagenome sex male

```
SRS21535716	pyraSARS	gut metagenome	age	66
SRS21535716	pyraSARS	gut metagenome	study_design	Case-control
SRS21535716	pyraSARS	gut metagenome	disease	Type 2 diabetic
SRS21535716	pyraSARS	gut metagenome	disease_ontology_id	9352
SRS21535716	pyraSARS	gut metagenome	study_title	Metataxonomic strategy to decipher gut bacteriome biomarkers of type 2 diabetes and obesity in Tunisian population
SRS21535716	pyraSARS	gut metagenome	continent	Africa
SRS21535716	pyraSARS	gut metagenome	region	North Africa
SRS21535716	pyraSARS	gut metagenome	assay_type	Amplicon
SRS21535716	pyraSARS	gut metagenome	technology	Illumina
SRS21535716	pyraSARS	gut metagenome	platform	Illumina MiSeq
SRS21535716	pyraSARS	gut metagenome	average_spotlength	601
SRS21535716	pyraSARS	gut metagenome	library_layout	Paired
SRS21535716	pyraSARS	gut metagenome	target_amplicon	V3-V4
SRS21535716	pyraSARS	gut metagenome	submitters	Meriem FASSATOUI/Abdelmajid ABID/Rym KEFI
SRS21535716	pyraSARS	gut metagenome	Isolate	BRAIN
SRS21535716	pyraSARS	gut metagenome	BioSampleModel	Metagenome or environmental
SRS21535717	HYPN56	gut metagenome	host	Homo sapiens
SRS21535717	HYPN56	gut metagenome	collection_date	2018
SRS21535717	HYPN56	gut metagenome	geo_loc_name	Tunisia
SRS21535717	HYPN56	gut metagenome	lat_lon	33.886917 N 9.537499 E
SRS21535717	HYPN56	gut metagenome	samp_size	110
SRS21535717	HYPN56	gut metagenome	participants_features	Adult
SRS21535717	HYPN56	gut metagenome	sex	female
SRS21535717	HYPN56	gut metagenome	age	44
SRS21535717	HYPN56	gut metagenome	study_design	Case-control
SRS21535717	HYPN56	gut metagenome	disease	Non Diabetic
SRS21535717	HYPN56	gut metagenome	study_title	Metataxonomic strategy to decipher gut bacteriome biomarkers of type 2 diabetes and obesity in Tunisian population
SRS21535717	HYPN56	gut metagenome	continent	Africa
SRS21535717	HYPN56	gut metagenome	region	North Africa
SRS21535717	HYPN56	gut metagenome	assay_type	Amplicon
SRS21535717	HYPN56	gut metagenome	technology	Illumina
SRS21535717	HYPN56	gut metagenome	platform	Illumina MiSeq
SRS21535717	HYPN56	gut metagenome	average_spotlength	601
SRS21535717	HYPN56	gut metagenome	library_layout	Paired
SRS21535717	HYPN56	gut metagenome	target_amplicon	V3-V4
SRS21535717	HYPN56	gut metagenome	submitters	Meriem FASSATOUI/Abdelmajid ABID/Rym KEFI
SRS21535717	HYPN56	gut metagenome	Isolate	mox
SRS21535717	HYPN56	gut metagenome	BioSampleModel	Metagenome or environmental
SRS21535718	AYFLU	gut metagenome	host	Homo sapiens
SRS21535718	AYFLU	gut metagenome	collection_date	2018
SRS21535718	AYFLU	gut metagenome	geo_loc_name	Tunisia
SRS21535718	AYFLU	gut metagenome	lat_lon	33.886917 N 9.537499 E
SRS21535718	AYFLU	gut metagenome	samp_size	110
SRS21535718	AYFLU	gut metagenome	participants_features	Adult
SRS21535718	AYFLU	gut metagenome	sex	female
SRS21535718	AYFLU	gut metagenome	age	77
SRS21535718	AYFLU	gut metagenome	study_design	Case-control
SRS21535718	AYFLU	gut metagenome	disease	Type 2 diabetic
SRS21535718	AYFLU	gut metagenome	disease_ontology_id	9352
SRS21535718	AYFLU	gut metagenome	study_title	Metataxonomic strategy to decipher gut bacteriome biomarkers of type 2 diabetes and obesity in Tunisian population
SRS21535718	AYFLU	gut metagenome	continent	Africa
SRS21535718	AYFLU	gut metagenome	region	North Africa
SRS21535718	AYFLU	gut metagenome	assay_type	Amplicon
SRS21535718	AYFLU	gut metagenome	technology	Illumina
SRS21535718	AYFLU	gut metagenome	platform	Illumina MiSeq
SRS21535718	AYFLU	gut metagenome	average_spotlength	601
SRS21535718	AYFLU	gut metagenome	library_layout	Paired
SRS21535718	AYFLU	gut metagenome	target_amplicon	V3-V4
SRS21535718	AYFLU	gut metagenome	submitters	Meriem FASSATOUI/Abdelmajid ABID/Rym KEFI
SRS21535718	AYFLU	gut metagenome	Isolate	mucine
SRS21535718	AYFLU	gut metagenome	BioSampleModel	Metagenome or environmental
SRS21535719	PRINTMARS	gut metagenome	host	Homo sapiens
SRS21535719	PRINTMARS	gut metagenome	collection_date	2018
SRS21535719	PRINTMARS	gut metagenome	geo_loc_name	Tunisia
SRS21535719	PRINTMARS	gut metagenome	lat_lon	33.886917 N 9.537499 E
```

SRS21535719 PRINTMARS gut metagenome samp_size 110
SRS21535719 PRINTMARS gut metagenome participants_features Adult
SRS21535719 PRINTMARS gut metagenome sex female
SRS21535719 PRINTMARS gut metagenome age 69
SRS21535719 PRINTMARS gut metagenome study_design Case-control
SRS21535719 PRINTMARS gut metagenome disease Type 2 diabetic
SRS21535719 PRINTMARS gut metagenome disease_ontology_id 9352
SRS21535719 PRINTMARS gut metagenome study_title Metataxonomic strategy to decipher gut bacteriome biomarkers of type 2 diabetes and obesity in Tunisian population
SRS21535719 PRINTMARS gut metagenome continent Africa
SRS21535719 PRINTMARS gut metagenome region North Africa
SRS21535719 PRINTMARS gut metagenome assay_type Amplicon
SRS21535719 PRINTMARS gut metagenome technology Illumina
SRS21535719 PRINTMARS gut metagenome platform Illumina MiSeq
SRS21535719 PRINTMARS gut metagenome average_spotlength 601
SRS21535719 PRINTMARS gut metagenome library_layout Paired
SRS21535719 PRINTMARS gut metagenome target_amplicon V3-V4
SRS21535719 PRINTMARS gut metagenome submitters Meriem FASSATOUI/Abdelmajid ABID/Rym KEFI
SRS21535719 PRINTMARS gut metagenome Isolate crete
SRS21535719 PRINTMARS gut metagenome BioSampleModel Metagenome or environmental
SRS21535720 YEPOX gut metagenome host Homo sapiens
SRS21535720 YEPOX gut metagenome collection_date 2018
SRS21535720 YEPOX gut metagenome geo_loc_name Tunisia
SRS21535720 YEPOX gut metagenome lat_lon 33.886917 N 9.537499 E
SRS21535720 YEPOX gut metagenome samp_size 110
SRS21535720 YEPOX gut metagenome participants_features Adult
SRS21535720 YEPOX gut metagenome sex male
SRS21535720 YEPOX gut metagenome age 66
SRS21535720 YEPOX gut metagenome study_design Case-control
SRS21535720 YEPOX gut metagenome disease Type 2 diabetic
SRS21535720 YEPOX gut metagenome disease_ontology_id 9352
SRS21535720 YEPOX gut metagenome study_title Metataxonomic strategy to decipher gut bacteriome biomarkers of type 2 diabetes and obesity in Tunisian population
SRS21535720 YEPOX gut metagenome continent Africa
SRS21535720 YEPOX gut metagenome region North Africa
SRS21535720 YEPOX gut metagenome assay_type Amplicon
SRS21535720 YEPOX gut metagenome technology Illumina
SRS21535720 YEPOX gut metagenome platform Illumina MiSeq
SRS21535720 YEPOX gut metagenome average_spotlength 601
SRS21535720 YEPOX gut metagenome library_layout Paired
SRS21535720 YEPOX gut metagenome target_amplicon V3-V4
SRS21535720 YEPOX gut metagenome submitters Meriem FASSATOUI/Abdelmajid ABID/Rym KEFI
SRS21535720 YEPOX gut metagenome Isolate garni
SRS21535720 YEPOX gut metagenome BioSampleModel Metagenome or environmental
SRS21535721 SIGNHAL gut metagenome host Homo sapiens
SRS21535721 SIGNHAL gut metagenome collection_date 2018
SRS21535721 SIGNHAL gut metagenome geo_loc_name Tunisia
SRS21535721 SIGNHAL gut metagenome lat_lon 33.886917 N 9.537499 E
SRS21535721 SIGNHAL gut metagenome samp_size 110
SRS21535721 SIGNHAL gut metagenome participants_features Adult
SRS21535721 SIGNHAL gut metagenome sex male
SRS21535721 SIGNHAL gut metagenome age 57
SRS21535721 SIGNHAL gut metagenome study_design Case-control
SRS21535721 SIGNHAL gut metagenome disease Type 2 diabetic
SRS21535721 SIGNHAL gut metagenome disease_ontology_id 9352
SRS21535721 SIGNHAL gut metagenome study_title Metataxonomic strategy to decipher gut bacteriome biomarkers of type 2 diabetes and obesity in Tunisian population
SRS21535721 SIGNHAL gut metagenome continent Africa
SRS21535721 SIGNHAL gut metagenome region North Africa
SRS21535721 SIGNHAL gut metagenome assay_type Amplicon
SRS21535721 SIGNHAL gut metagenome technology Illumina
SRS21535721 SIGNHAL gut metagenome platform Illumina MiSeq
SRS21535721 SIGNHAL gut metagenome average_spotlength 601
SRS21535721 SIGNHAL gut metagenome library_layout Paired
SRS21535721 SIGNHAL gut metagenome target_amplicon V3-V4
SRS21535721 SIGNHAL gut metagenome submitters Meriem FASSATOUI/Abdelmajid ABID/Rym KEFI
SRS21535721 SIGNHAL gut metagenome Isolate jarre
SRS21535721 SIGNHAL gut metagenome BioSampleModel Metagenome or environmental

SRS21535722 HCfAMA gut metagenome host Homo sapiens
SRS21535722 HCfAMA gut metagenome collection_date 2018
SRS21535722 HCfAMA gut metagenome geo_loc_name Tunisia
SRS21535722 HCfAMA gut metagenome lat_lon 33.886917 N 9.537499 E
SRS21535722 HCfAMA gut metagenome samp_size 110
SRS21535722 HCfAMA gut metagenome participants_features Adult
SRS21535722 HCfAMA gut metagenome sex female
SRS21535722 HCfAMA gut metagenome age 57
SRS21535722 HCfAMA gut metagenome study_design Case-control
SRS21535722 HCfAMA gut metagenome disease Type 2 diabetic
SRS21535722 HCfAMA gut metagenome disease_ontology_id 9352
SRS21535722 HCfAMA gut metagenome study_title Metataxonomic strategy to decipher gut bacteriome biomarkers of type 2 diabetes and obesity in Tunisian population
SRS21535722 HCfAMA gut metagenome continent Africa
SRS21535722 HCfAMA gut metagenome region North Africa
SRS21535722 HCfAMA gut metagenome assay_type Amplicon
SRS21535722 HCfAMA gut metagenome technology Illumina
SRS21535722 HCfAMA gut metagenome platform Illumina MiSeq
SRS21535722 HCfAMA gut metagenome average_spotlength 601
SRS21535722 HCfAMA gut metagenome library_layout Paired
SRS21535722 HCfAMA gut metagenome target_amplicon V3-V4
SRS21535722 HCfAMA gut metagenome submitters Meriem FASSATOUI/Abdelmajid ABID/Rym KEFI
SRS21535722 HCfAMA gut metagenome Isolate gourd
SRS21535722 HCfAMA gut metagenome BioSampleModel Metagenome or environmental
SRS21535723 YSmidd gut metagenome host Homo sapiens
SRS21535723 YSmidd gut metagenome collection_date 2018
SRS21535723 YSmidd gut metagenome geo_loc_name Tunisia
SRS21535723 YSmidd gut metagenome lat_lon 33.886917 N 9.537499 E
SRS21535723 YSmidd gut metagenome samp_size 110
SRS21535723 YSmidd gut metagenome participants_features Adult
SRS21535723 YSmidd gut metagenome sex male
SRS21535723 YSmidd gut metagenome age 72
SRS21535723 YSmidd gut metagenome study_design Case-control
SRS21535723 YSmidd gut metagenome disease Type 2 diabetic
SRS21535723 YSmidd gut metagenome disease_ontology_id 9352
SRS21535723 YSmidd gut metagenome study_title Metataxonomic strategy to decipher gut bacteriome biomarkers of type 2 diabetes and obesity in Tunisian population
SRS21535723 YSmidd gut metagenome continent Africa
SRS21535723 YSmidd gut metagenome region North Africa
SRS21535723 YSmidd gut metagenome assay_type Amplicon
SRS21535723 YSmidd gut metagenome technology Illumina
SRS21535723 YSmidd gut metagenome platform Illumina MiSeq
SRS21535723 YSmidd gut metagenome average_spotlength 601
SRS21535723 YSmidd gut metagenome library_layout Paired
SRS21535723 YSmidd gut metagenome target_amplicon V3-V4
SRS21535723 YSmidd gut metagenome submitters Meriem FASSATOUI/Abdelmajid ABID/Rym KEFI
SRS21535723 YSmidd gut metagenome Isolate ver
SRS21535723 YSmidd gut metagenome BioSampleModel Metagenome or environmental
SRS21535724 NOTCHdEAF gut metagenome host Homo sapiens
SRS21535724 NOTCHdEAF gut metagenome collection_date 2018
SRS21535724 NOTCHdEAF gut metagenome geo_loc_name Tunisia
SRS21535724 NOTCHdEAF gut metagenome lat_lon 33.886917 N 9.537499 E
SRS21535724 NOTCHdEAF gut metagenome samp_size 110
SRS21535724 NOTCHdEAF gut metagenome participants_features Adult
SRS21535724 NOTCHdEAF gut metagenome sex male
SRS21535724 NOTCHdEAF gut metagenome age 60
SRS21535724 NOTCHdEAF gut metagenome study_design Case-control
SRS21535724 NOTCHdEAF gut metagenome disease Type 2 diabetic
SRS21535724 NOTCHdEAF gut metagenome disease_ontology_id 9352
SRS21535724 NOTCHdEAF gut metagenome study_title Metataxonomic strategy to decipher gut bacteriome biomarkers of type 2 diabetes and obesity in Tunisian population
SRS21535724 NOTCHdEAF gut metagenome continent Africa
SRS21535724 NOTCHdEAF gut metagenome region North Africa
SRS21535724 NOTCHdEAF gut metagenome assay_type Amplicon
SRS21535724 NOTCHdEAF gut metagenome technology Illumina
SRS21535724 NOTCHdEAF gut metagenome platform Illumina MiSeq
SRS21535724 NOTCHdEAF gut metagenome average_spotlength 601
SRS21535724 NOTCHdEAF gut metagenome library_layout Paired
SRS21535724 NOTCHdEAF gut metagenome target_amplicon V3-V4

SRS21535724 NOTCHdEAF gut metagenome submitters Meriem FASSATOUI/Abdelmajid ABID/Rym KEFI
SRS21535724 NOTCHdEAF gut metagenome Isolate lyme
SRS21535724 NOTCHdEAF gut metagenome BioSampleModel Metagenome or environmental
SRS21535725 AYURV gut metagenome host Homo sapiens
SRS21535725 AYURV gut metagenome collection_date 2018
SRS21535725 AYURV gut metagenome geo_loc_name Tunisia
SRS21535725 AYURV gut metagenome lat_lon 33.886917 N 9.537499 E
SRS21535725 AYURV gut metagenome samp_size 110
SRS21535725 AYURV gut metagenome participants_features Adult
SRS21535725 AYURV gut metagenome sex female
SRS21535725 AYURV gut metagenome age 60
SRS21535725 AYURV gut metagenome study_design Case-control
SRS21535725 AYURV gut metagenome disease Type 2 diabetic
SRS21535725 AYURV gut metagenome disease_ontology_id 9352
SRS21535725 AYURV gut metagenome study_title Metataxonomic strategy to decipher gut bacteriome biomarkers of type 2 diabetes and obesity in Tunisian population
SRS21535725 AYURV gut metagenome continent Africa
SRS21535725 AYURV gut metagenome region North Africa
SRS21535725 AYURV gut metagenome assay_type Amplicon
SRS21535725 AYURV gut metagenome technology Illumina
SRS21535725 AYURV gut metagenome platform Illumina MiSeq
SRS21535725 AYURV gut metagenome average_spotlength 601
SRS21535725 AYURV gut metagenome library_layout Paired
SRS21535725 AYURV gut metagenome target_amplicon V3-V4
SRS21535725 AYURV gut metagenome submitters Meriem FASSATOUI/Abdelmajid ABID/Rym KEFI
SRS21535725 AYURV gut metagenome Isolate westni
SRS21535725 AYURV gut metagenome BioSampleModel Metagenome or environmental
SRS21535726 YUGALe gut metagenome host Homo sapiens
SRS21535726 YUGALe gut metagenome collection_date 2018
SRS21535726 YUGALe gut metagenome geo_loc_name Tunisia
SRS21535726 YUGALe gut metagenome lat_lon 33.886917 N 9.537499 E
SRS21535726 YUGALe gut metagenome samp_size 110
SRS21535726 YUGALe gut metagenome participants_features Adult
SRS21535726 YUGALe gut metagenome sex male
SRS21535726 YUGALe gut metagenome age 48
SRS21535726 YUGALe gut metagenome study_design Case-control
SRS21535726 YUGALe gut metagenome disease Type 2 diabetic
SRS21535726 YUGALe gut metagenome disease_ontology_id 9352
SRS21535726 YUGALe gut metagenome study_title Metataxonomic strategy to decipher gut bacteriome biomarkers of type 2 diabetes and obesity in Tunisian population
SRS21535726 YUGALe gut metagenome continent Africa
SRS21535726 YUGALe gut metagenome region North Africa
SRS21535726 YUGALe gut metagenome assay_type Amplicon
SRS21535726 YUGALe gut metagenome technology Illumina
SRS21535726 YUGALe gut metagenome platform Illumina MiSeq
SRS21535726 YUGALe gut metagenome average_spotlength 601
SRS21535726 YUGALe gut metagenome library_layout Paired
SRS21535726 YUGALe gut metagenome target_amplicon V3-V4
SRS21535726 YUGALe gut metagenome submitters Meriem FASSATOUI/Abdelmajid ABID/Rym KEFI
SRS21535726 YUGALe gut metagenome Isolate ebol
SRS21535726 YUGALe gut metagenome BioSampleModel Metagenome or environmental
SRS21535727 SIMSmean gut metagenome host Homo sapiens
SRS21535727 SIMSmean gut metagenome collection_date 2018
SRS21535727 SIMSmean gut metagenome geo_loc_name Tunisia
SRS21535727 SIMSmean gut metagenome lat_lon 33.886917 N 9.537499 E
SRS21535727 SIMSmean gut metagenome samp_size 110
SRS21535727 SIMSmean gut metagenome participants_features Adult
SRS21535727 SIMSmean gut metagenome sex male
SRS21535727 SIMSmean gut metagenome age 66
SRS21535727 SIMSmean gut metagenome study_design Case-control
SRS21535727 SIMSmean gut metagenome disease Type 2 diabetic
SRS21535727 SIMSmean gut metagenome disease_ontology_id 9352
SRS21535727 SIMSmean gut metagenome study_title Metataxonomic strategy to decipher gut bacteriome biomarkers of type 2 diabetes and obesity in Tunisian population
SRS21535727 SIMSmean gut metagenome continent Africa
SRS21535727 SIMSmean gut metagenome region North Africa
SRS21535727 SIMSmean gut metagenome assay_type Amplicon
SRS21535727 SIMSmean gut metagenome technology Illumina

```
SRS21535727	SIMSmean	gut metagenome	platform	Illumina MiSeq
SRS21535727	SIMSmean	gut metagenome	average_spotlength	601
SRS21535727	SIMSmean	gut metagenome	library_layout	Paired
SRS21535727	SIMSmean	gut metagenome	target_amplicon	V3-V4
SRS21535727	SIMSmean	gut metagenome	submitters	Meriem FASSATOUI/Abdelmajid
ABID/Rym KEFI
SRS21535727	SIMSmean	gut metagenome	Isolate	leishma
SRS21535727	SIMSmean	gut metagenome	BioSampleModel	Metagenome or environmental
SRS21535728	JIH5n	gut metagenome	host	Homo sapiens
SRS21535728	JIH5n	gut metagenome	collection_date	2018
SRS21535728	JIH5n	gut metagenome	geo_loc_name	Tunisia
SRS21535728	JIH5n	gut metagenome	lat_lon	33.886917 N 9.537499 E
SRS21535728	JIH5n	gut metagenome	samp_size	110
SRS21535728	JIH5n	gut metagenome	participants_features	Adult
SRS21535728	JIH5n	gut metagenome	sex	female
SRS21535728	JIH5n	gut metagenome	age	44
SRS21535728	JIH5n	gut metagenome	study_design	Case-control
SRS21535728	JIH5n	gut metagenome	disease	Non Diabetic
SRS21535728	JIH5n	gut metagenome	study_title	Metataxonomic strategy to decipher gut
bacteriome biomarkers of type 2 diabetes and obesity in Tunisian population
SRS21535728	JIH5n	gut metagenome	continent	Africa
SRS21535728	JIH5n	gut metagenome	region	North Africa
SRS21535728	JIH5n	gut metagenome	assay_type	Amplicon
SRS21535728	JIH5n	gut metagenome	technology	Illumina
SRS21535728	JIH5n	gut metagenome	platform	Illumina MiSeq
SRS21535728	JIH5n	gut metagenome	average_spotlength	595
SRS21535728	JIH5n	gut metagenome	library_layout	Paired
SRS21535728	JIH5n	gut metagenome	target_amplicon	V3-V4
SRS21535728	JIH5n	gut metagenome	submitters	Meriem FASSATOUI/Abdelmajid ABID/Rym KEFI
SRS21535728	JIH5n	gut metagenome	Isolate	yl
SRS21535728	JIH5n	gut metagenome	BioSampleModel	Metagenome or environmental
SRS21535729	LaiGIF	gut metagenome	host	Homo sapiens
SRS21535729	LaiGIF	gut metagenome	collection_date	2018
SRS21535729	LaiGIF	gut metagenome	geo_loc_name	Tunisia
SRS21535729	LaiGIF	gut metagenome	lat_lon	33.886917 N 9.537499 E
SRS21535729	LaiGIF	gut metagenome	samp_size	110
SRS21535729	LaiGIF	gut metagenome	participants_features	Adult
SRS21535729	LaiGIF	gut metagenome	sex	male
SRS21535729	LaiGIF	gut metagenome	age	48
SRS21535729	LaiGIF	gut metagenome	study_design	Case-control
SRS21535729	LaiGIF	gut metagenome	disease	Type 2 diabetic
SRS21535729	LaiGIF	gut metagenome	disease_ontology_id	9352
SRS21535729	LaiGIF	gut metagenome	study_title	Metataxonomic strategy to decipher gut
bacteriome biomarkers of type 2 diabetes and obesity in Tunisian population
SRS21535729	LaiGIF	gut metagenome	continent	Africa
SRS21535729	LaiGIF	gut metagenome	region	North Africa
SRS21535729	LaiGIF	gut metagenome	assay_type	Amplicon
SRS21535729	LaiGIF	gut metagenome	technology	Illumina
SRS21535729	LaiGIF	gut metagenome	platform	Illumina MiSeq
SRS21535729	LaiGIF	gut metagenome	average_spotlength	601
SRS21535729	LaiGIF	gut metagenome	library_layout	Paired
SRS21535729	LaiGIF	gut metagenome	target_amplicon	V3-V4
SRS21535729	LaiGIF	gut metagenome	submitters	Meriem FASSATOUI/Abdelmajid ABID/Rym KEFI
SRS21535729	LaiGIF	gut metagenome	Isolate	nia
SRS21535729	LaiGIF	gut metagenome	BioSampleModel	Metagenome or environmental
SRS21535730	CAMasf	gut metagenome	host	Homo sapiens
SRS21535730	CAMasf	gut metagenome	collection_date	2018
SRS21535730	CAMasf	gut metagenome	geo_loc_name	Tunisia
SRS21535730	CAMasf	gut metagenome	lat_lon	33.886917 N 9.537499 E
SRS21535730	CAMasf	gut metagenome	samp_size	110
SRS21535730	CAMasf	gut metagenome	participants_features	Adult
SRS21535730	CAMasf	gut metagenome	sex	female
SRS21535730	CAMasf	gut metagenome	age	66
SRS21535730	CAMasf	gut metagenome	study_design	Case-control
SRS21535730	CAMasf	gut metagenome	disease	Type 2 diabetic
SRS21535730	CAMasf	gut metagenome	disease_ontology_id	9352
SRS21535730	CAMasf	gut metagenome	study_title	Metataxonomic strategy to decipher gut
bacteriome biomarkers of type 2 diabetes and obesity in Tunisian population
SRS21535730	CAMasf	gut metagenome	continent	Africa
```

```
SRS21535730	CAMasf	gut metagenome	region	North Africa
SRS21535730	CAMasf	gut metagenome	assay_type	Amplicon
SRS21535730	CAMasf	gut metagenome	technology	Illumina
SRS21535730	CAMasf	gut metagenome	platform	Illumina MiSeq
SRS21535730	CAMasf	gut metagenome	average_spotlength	601
SRS21535730	CAMasf	gut metagenome	library_layout	Paired
SRS21535730	CAMasf	gut metagenome	target_amplicon	V3-V4
SRS21535730	CAMasf	gut metagenome	submitters	Meriem FASSATOUI/Abdelmajid ABID/Rym KEFI
SRS21535730	CAMasf	gut metagenome	Isolate	BRUC
SRS21535730	CAMasf	gut metagenome	BioSampleModel	Metagenome or environmental
SRS21535731	HEADes	gut metagenome	host	Homo sapiens
SRS21535731	HEADes	gut metagenome	collection_date	2018
SRS21535731	HEADes	gut metagenome	geo_loc_name	Tunisia
SRS21535731	HEADes	gut metagenome	lat_lon	33.886917 N 9.537499 E
SRS21535731	HEADes	gut metagenome	samp_size	110
SRS21535731	HEADes	gut metagenome	participants_features	Adult
SRS21535731	HEADes	gut metagenome	sex	female
SRS21535731	HEADes	gut metagenome	age	59
SRS21535731	HEADes	gut metagenome	study_design	Case-control
SRS21535731	HEADes	gut metagenome	disease	Type 2 diabetic
SRS21535731	HEADes	gut metagenome	disease_ontology_id	9352
SRS21535731	HEADes	gut metagenome	study_title	Metataxonomic strategy to decipher gut
bacteriome biomarkers of type 2 diabetes and obesity in Tunisian population
SRS21535731	HEADes	gut metagenome	continent	Africa
SRS21535731	HEADes	gut metagenome	region	North Africa
SRS21535731	HEADes	gut metagenome	assay_type	Amplicon
SRS21535731	HEADes	gut metagenome	technology	Illumina
SRS21535731	HEADes	gut metagenome	platform	Illumina MiSeq
SRS21535731	HEADes	gut metagenome	average_spotlength	601
SRS21535731	HEADes	gut metagenome	library_layout	Paired
SRS21535731	HEADes	gut metagenome	target_amplicon	V3-V4
SRS21535731	HEADes	gut metagenome	submitters	Meriem FASSATOUI/Abdelmajid ABID/Rym KEFI
SRS21535731	HEADes	gut metagenome	Isolate	CHLOROF
SRS21535731	HEADes	gut metagenome	BioSampleModel	Metagenome or environmental
SRS21535732	HERPSimplex	gut metagenome	host	Homo sapiens
SRS21535732	HERPSimplex	gut metagenome	collection_date	2018
SRS21535732	HERPSimplex	gut metagenome	geo_loc_name	Tunisia
SRS21535732	HERPSimplex	gut metagenome	lat_lon	33.886917 N 9.537499 E
SRS21535732	HERPSimplex	gut metagenome	samp_size	110
SRS21535732	HERPSimplex	gut metagenome	participants_features	Adult
SRS21535732	HERPSimplex	gut metagenome	sex	female
SRS21535732	HERPSimplex	gut metagenome	age	55
SRS21535732	HERPSimplex	gut metagenome	study_design	Case-control
SRS21535732	HERPSimplex	gut metagenome	disease	Type 2 diabetic
SRS21535732	HERPSimplex	gut metagenome	disease_ontology_id	9352
SRS21535732	HERPSimplex	gut metagenome	study_title	Metataxonomic strategy to decipher
gut bacteriome biomarkers of type 2 diabetes and obesity in Tunisian population
SRS21535732	HERPSimplex	gut metagenome	continent	Africa
SRS21535732	HERPSimplex	gut metagenome	region	North Africa
SRS21535732	HERPSimplex	gut metagenome	assay_type	Amplicon
SRS21535732	HERPSimplex	gut metagenome	technology	Illumina
SRS21535732	HERPSimplex	gut metagenome	platform	Illumina MiSeq
SRS21535732	HERPSimplex	gut metagenome	average_spotlength	601
SRS21535732	HERPSimplex	gut metagenome	library_layout	Paired
SRS21535647	TGM	gut metagenome	assay_type	Amplicon
SRS21535647	TGM	gut metagenome	technology	Illumina
SRS21535647	TGM	gut metagenome	platform	Illumina MiSeq
SRS21535647	TGM	gut metagenome	average_spotlength	601
SRS21535647	TGM	gut metagenome	library_layout	Paired
SRS21535647	TGM	gut metagenome	target_amplicon	V3-V4
SRS21535647	TGM	gut metagenome	submitters	Meriem FASSATOUI/Abdelmajid ABID/Rym KEFI
SRS21535647	TGM	gut metagenome	Isolate	VIR
SRS21535647	TGM	gut metagenome	BioSampleModel	Metagenome or environmental
SRS21535648	CIC20	gut metagenome	host	Homo sapiens
SRS21535648	CIC20	gut metagenome	collection_date	2018
SRS21535648	CIC20	gut metagenome	geo_loc_name	Tunisia
SRS21535648	CIC20	gut metagenome	lat_lon	33.886917 N 9.537499 E
SRS21535648	CIC20	gut metagenome	samp_size	110
SRS21535648	CIC20	gut metagenome	participants_features	Adult
```

SRS21535648 CIC20 gut metagenome sex female
SRS21535648 CIC20 gut metagenome age 58
SRS21535648 CIC20 gut metagenome study_design Case-control
SRS21535648 CIC20 gut metagenome disease Non Diabetic
SRS21535648 CIC20 gut metagenome study_title Metataxonomic strategy to decipher gut bacteriome biomarkers of type 2 diabetes and obesity in Tunisian population
SRS21535648 CIC20 gut metagenome continent Africa
SRS21535648 CIC20 gut metagenome region North Africa
SRS21535648 CIC20 gut metagenome assay_type Amplicon
SRS21535648 CIC20 gut metagenome technology Illumina
SRS21535648 CIC20 gut metagenome platform Illumina MiSeq
SRS21535648 CIC20 gut metagenome average_spotlength 595
SRS21535648 CIC20 gut metagenome library_layout Paired
SRS21535648 CIC20 gut metagenome target_amplicon V3-V4
SRS21535648 CIC20 gut metagenome submitters Meriem FASSATOUI/Abdelmajid ABID/Rym KEFI
SRS21535648 CIC20 gut metagenome Isolate min
SRS21535648 CIC20 gut metagenome BioSampleModel Metagenome or environmental
SRS21535649 mER67 gut metagenome host Homo sapiens
SRS21535649 mER67 gut metagenome collection_date 2018
SRS21535649 mER67 gut metagenome geo_loc_name Tunisia
SRS21535649 mER67 gut metagenome lat_lon 33.886917 N 9.537499 E
SRS21535649 mER67 gut metagenome samp_size 110
SRS21535649 mER67 gut metagenome participants_features Adult
SRS21535649 mER67 gut metagenome sex female
SRS21535649 mER67 gut metagenome age 42
SRS21535649 mER67 gut metagenome study_design Case-control
SRS21535649 mER67 gut metagenome disease Non Diabetic
SRS21535649 mER67 gut metagenome study_title Metataxonomic strategy to decipher gut bacteriome biomarkers of type 2 diabetes and obesity in Tunisian population
SRS21535649 mER67 gut metagenome continent Africa
SRS21535649 mER67 gut metagenome region North Africa
SRS21535649 mER67 gut metagenome assay_type Amplicon
SRS21535649 mER67 gut metagenome technology Illumina
SRS21535649 mER67 gut metagenome platform Illumina MiSeq
SRS21535649 mER67 gut metagenome average_spotlength 601
SRS21535649 mER67 gut metagenome library_layout Paired
SRS21535649 mER67 gut metagenome target_amplicon V3-V4
SRS21535649 mER67 gut metagenome submitters Meriem FASSATOUI/Abdelmajid ABID/Rym KEFI
SRS21535649 mER67 gut metagenome Isolate EFFE
SRS21535649 mER67 gut metagenome BioSampleModel Metagenome or environmental
SRS21535650 987MANIF gut metagenome host Homo sapiens
SRS21535650 987MANIF gut metagenome collection_date 2018
SRS21535650 987MANIF gut metagenome geo_loc_name Tunisia
SRS21535650 987MANIF gut metagenome lat_lon 33.886917 N 9.537499 E
SRS21535650 987MANIF gut metagenome samp_size 110
SRS21535650 987MANIF gut metagenome participants_features Adult
SRS21535650 987MANIF gut metagenome sex female
SRS21535650 987MANIF gut metagenome age 47
SRS21535650 987MANIF gut metagenome study_design Case-control
SRS21535650 987MANIF gut metagenome disease Non Diabetic
SRS21535650 987MANIF gut metagenome study_title Metataxonomic strategy to decipher gut bacteriome biomarkers of type 2 diabetes and obesity in Tunisian population
SRS21535650 987MANIF gut metagenome continent Africa
SRS21535650 987MANIF gut metagenome region North Africa
SRS21535650 987MANIF gut metagenome assay_type Amplicon
SRS21535650 987MANIF gut metagenome technology Illumina
SRS21535650 987MANIF gut metagenome platform Illumina MiSeq
SRS21535650 987MANIF gut metagenome average_spotlength 601
SRS21535650 987MANIF gut metagenome library_layout Paired
SRS21535650 987MANIF gut metagenome target_amplicon V3-V4
SRS21535650 987MANIF gut metagenome submitters Meriem FASSATOUI/Abdelmajid ABID/Rym KEFI
SRS21535650 987MANIF gut metagenome Isolate RAL
SRS21535650 987MANIF gut metagenome BioSampleModel Metagenome or environmental
SRS21535651 pHAGEW gut metagenome host Homo sapiens
SRS21535651 pHAGEW gut metagenome collection_date 2018
SRS21535651 pHAGEW gut metagenome geo_loc_name Tunisia
SRS21535651 pHAGEW gut metagenome lat_lon 33.886917 N 9.537499 E
SRS21535651 pHAGEW gut metagenome samp_size 110

SRS21535651 pHAGEW gut metagenome participants_features Adult
SRS21535651 pHAGEW gut metagenome sex female
SRS21535651 pHAGEW gut metagenome age 47
SRS21535651 pHAGEW gut metagenome study_design Case-control
SRS21535651 pHAGEW gut metagenome disease Non Diabetic
SRS21535651 pHAGEW gut metagenome study_title Metataxonomic strategy to decipher gut bacteriome biomarkers of type 2 diabetes and obesity in Tunisian population
SRS21535651 pHAGEW gut metagenome continent Africa
SRS21535651 pHAGEW gut metagenome region North Africa
SRS21535651 pHAGEW gut metagenome assay_type Amplicon
SRS21535651 pHAGEW gut metagenome technology Illumina
SRS21535651 pHAGEW gut metagenome platform Illumina MiSeq
SRS21535651 pHAGEW gut metagenome average_spotlength 595
SRS21535651 pHAGEW gut metagenome library_layout Paired
SRS21535651 pHAGEW gut metagenome target_amplicon V3-V4
SRS21535651 pHAGEW gut metagenome submitters Meriem FASSATOUI/Abdelmajid ABID/Rym KEFI
SRS21535651 pHAGEW gut metagenome Isolate GON
SRS21535651 pHAGEW gut metagenome BioSampleModel Metagenome or environmental
SRS21535652 XABFAR gut metagenome host Homo sapiens
SRS21535652 XABFAR gut metagenome collection_date 2018
SRS21535652 XABFAR gut metagenome geo_loc_name Tunisia
SRS21535652 XABFAR gut metagenome lat_lon 33.886917 N 9.537499 E
SRS21535652 XABFAR gut metagenome samp_size 110
SRS21535652 XABFAR gut metagenome participants_features Adult
SRS21535652 XABFAR gut metagenome sex female
SRS21535652 XABFAR gut metagenome age 67
SRS21535652 XABFAR gut metagenome study_design Case-control
SRS21535652 XABFAR gut metagenome disease Non Diabetic
SRS21535652 XABFAR gut metagenome study_title Metataxonomic strategy to decipher gut bacteriome biomarkers of type 2 diabetes and obesity in Tunisian population
SRS21535652 XABFAR gut metagenome continent Africa
SRS21535652 XABFAR gut metagenome region North Africa
SRS21535652 XABFAR gut metagenome assay_type Amplicon
SRS21535652 XABFAR gut metagenome technology Illumina
SRS21535652 XABFAR gut metagenome platform Illumina MiSeq
SRS21535652 XABFAR gut metagenome average_spotlength 601
SRS21535652 XABFAR gut metagenome library_layout Paired
SRS21535652 XABFAR gut metagenome target_amplicon V3-V4
SRS21535652 XABFAR gut metagenome submitters Meriem FASSATOUI/Abdelmajid ABID/Rym KEFI
SRS21535652 XABFAR gut metagenome Isolate VIT
SRS21535652 XABFAR gut metagenome BioSampleModel Metagenome or environmental
SRS21535653 YLABO4 gut metagenome host Homo sapiens
SRS21535653 YLABO4 gut metagenome collection_date 2018
SRS21535653 YLABO4 gut metagenome geo_loc_name Tunisia
SRS21535653 YLABO4 gut metagenome lat_lon 33.886917 N 9.537499 E
SRS21535653 YLABO4 gut metagenome samp_size 110
SRS21535653 YLABO4 gut metagenome participants_features Adult
SRS21535653 YLABO4 gut metagenome sex female
SRS21535653 YLABO4 gut metagenome age 64
SRS21535653 YLABO4 gut metagenome study_design Case-control
SRS21535653 YLABO4 gut metagenome disease Non Diabetic
SRS21535653 YLABO4 gut metagenome study_title Metataxonomic strategy to decipher gut bacteriome biomarkers of type 2 diabetes and obesity in Tunisian population
SRS21535653 YLABO4 gut metagenome continent Africa
SRS21535653 YLABO4 gut metagenome region North Africa
SRS21535653 YLABO4 gut metagenome assay_type Amplicon
SRS21535653 YLABO4 gut metagenome technology Illumina
SRS21535653 YLABO4 gut metagenome platform Illumina MiSeq
SRS21535653 YLABO4 gut metagenome average_spotlength 595
SRS21535653 YLABO4 gut metagenome library_layout Paired
SRS21535653 YLABO4 gut metagenome target_amplicon V3-V4
SRS21535653 YLABO4 gut metagenome submitters Meriem FASSATOUI/Abdelmajid ABID/Rym KEFI
SRS21535653 YLABO4 gut metagenome Isolate B12
SRS21535653 YLABO4 gut metagenome BioSampleModel Metagenome or environmental
SRS21535654 FIRMTBGE gut metagenome host Homo sapiens
SRS21535654 FIRMTBGE gut metagenome collection_date 2018
SRS21535654 FIRMTBGE gut metagenome geo_loc_name Tunisia
SRS21535654 FIRMTBGE gut metagenome lat_lon 33.886917 N 9.537499 E
SRS21535654 FIRMTBGE gut metagenome samp_size 110

SRS21535654 FIRMTBGE gut metagenome participants_features Adult
SRS21535654 FIRMTBGE gut metagenome sex male
SRS21535654 FIRMTBGE gut metagenome age 73
SRS21535654 FIRMTBGE gut metagenome study_design Case-control
SRS21535654 FIRMTBGE gut metagenome disease Non Diabetic
SRS21535654 FIRMTBGE gut metagenome study_title Metataxonomic strategy to decipher gut bacteriome biomarkers of type 2 diabetes and obesity in Tunisian population
SRS21535654 FIRMTBGE gut metagenome continent Africa
SRS21535654 FIRMTBGE gut metagenome region North Africa
SRS21535654 FIRMTBGE gut metagenome assay_type Amplicon
SRS21535654 FIRMTBGE gut metagenome technology Illumina
SRS21535654 FIRMTBGE gut metagenome platform Illumina MiSeq
SRS21535654 FIRMTBGE gut metagenome average_spotlength 601
SRS21535654 FIRMTBGE gut metagenome library_layout Paired
SRS21535654 FIRMTBGE gut metagenome target_amplicon V3-V4
SRS21535654 FIRMTBGE gut metagenome submitters Meriem FASSATOUI/Abdelmajid ABID/Rym KEFI
SRS21535654 FIRMTBGE gut metagenome Isolate FOLATE
SRS21535654 FIRMTBGE gut metagenome BioSampleModel Metagenome or environmental
SRS21535655 EBOU gut metagenome host Homo sapiens
SRS21535655 EBOU gut metagenome collection_date 2018
SRS21535655 EBOU gut metagenome geo_loc_name Tunisia
SRS21535655 EBOU gut metagenome lat_lon 33.886917 N 9.537499 E
SRS21535655 EBOU gut metagenome samp_size 110
SRS21535655 EBOU gut metagenome participants_features Adult
SRS21535655 EBOU gut metagenome sex female
SRS21535655 EBOU gut metagenome age 54
SRS21535655 EBOU gut metagenome study_design Case-control
SRS21535655 EBOU gut metagenome disease Non Diabetic
SRS21535655 EBOU gut metagenome study_title Metataxonomic strategy to decipher gut bacteriome biomarkers of type 2 diabetes and obesity in Tunisian population
SRS21535655 EBOU gut metagenome continent Africa
SRS21535655 EBOU gut metagenome region North Africa
SRS21535655 EBOU gut metagenome assay_type Amplicon
SRS21535655 EBOU gut metagenome technology Illumina
SRS21535655 EBOU gut metagenome platform Illumina MiSeq
SRS21535655 EBOU gut metagenome average_spotlength 601
SRS21535655 EBOU gut metagenome library_layout Paired
SRS21535655 EBOU gut metagenome target_amplicon V3-V4
SRS21535655 EBOU gut metagenome submitters Meriem FASSATOUI/Abdelmajid ABID/Rym KEFI
SRS21535655 EBOU gut metagenome Isolate FER
SRS21535655 EBOU gut metagenome BioSampleModel Metagenome or environmental
SRS21535656 NHKJ gut metagenome host Homo sapiens
SRS21535656 NHKJ gut metagenome collection_date 2018
SRS21535656 NHKJ gut metagenome geo_loc_name Tunisia
SRS21535656 NHKJ gut metagenome lat_lon 33.886917 N 9.537499 E
SRS21535656 NHKJ gut metagenome samp_size 110
SRS21535656 NHKJ gut metagenome participants_features Adult
SRS21535656 NHKJ gut metagenome sex female
SRS21535656 NHKJ gut metagenome age 50
SRS21535656 NHKJ gut metagenome study_design Case-control
SRS21535656 NHKJ gut metagenome disease Non Diabetic
SRS21535656 NHKJ gut metagenome study_title Metataxonomic strategy to decipher gut bacteriome biomarkers of type 2 diabetes and obesity in Tunisian population
SRS21535656 NHKJ gut metagenome continent Africa
SRS21535656 NHKJ gut metagenome region North Africa
SRS21535656 NHKJ gut metagenome assay_type Amplicon
SRS21535656 NHKJ gut metagenome technology Illumina
SRS21535656 NHKJ gut metagenome platform Illumina MiSeq
SRS21535656 NHKJ gut metagenome average_spotlength 601
SRS21535656 NHKJ gut metagenome library_layout Paired
SRS21535656 NHKJ gut metagenome target_amplicon V3-V4
SRS21535656 NHKJ gut metagenome submitters Meriem FASSATOUI/Abdelmajid ABID/Rym KEFI
SRS21535656 NHKJ gut metagenome Isolate VITD
SRS21535656 NHKJ gut metagenome BioSampleModel Metagenome or environmental
SRS21535657 WESTERN gut metagenome host Homo sapiens
SRS21535657 WESTERN gut metagenome collection_date 2018
SRS21535657 WESTERN gut metagenome geo_loc_name Tunisia
SRS21535657 WESTERN gut metagenome lat_lon 33.886917 N 9.537499 E

SRS21535657 WESTERN gut metagenome samp_size 110
SRS21535657 WESTERN gut metagenome participants_features Adult
SRS21535657 WESTERN gut metagenome sex female
SRS21535657 WESTERN gut metagenome age 58
SRS21535657 WESTERN gut metagenome study_design Case-control
SRS21535657 WESTERN gut metagenome disease Non Diabetic
SRS21535657 WESTERN gut metagenome study_title Metataxonomic strategy to decipher gut bacteriome biomarkers of type 2 diabetes and obesity in Tunisian population
SRS21535657 WESTERN gut metagenome continent Africa
SRS21535657 WESTERN gut metagenome region North Africa
SRS21535657 WESTERN gut metagenome assay_type Amplicon
SRS21535657 WESTERN gut metagenome technology Illumina
SRS21535657 WESTERN gut metagenome platform Illumina MiSeq
SRS21535657 WESTERN gut metagenome average_spotlength 601
SRS21535657 WESTERN gut metagenome library_layout Paired
SRS21535657 WESTERN gut metagenome target_amplicon V3-V4
SRS21535657 WESTERN gut metagenome submitters Meriem FASSATOUI/Abdelmajid ABID/Rym KEFI
SRS21535657 WESTERN gut metagenome Isolate SUN
SRS21535657 WESTERN gut metagenome BioSampleModel Metagenome or environmental
SRS21535658 dOOGlo gut metagenome host Homo sapiens
SRS21535658 dOOGlo gut metagenome collection_date 2018
SRS21535658 dOOGlo gut metagenome geo_loc_name Tunisia
SRS21535658 dOOGlo gut metagenome lat_lon 33.886917 N 9.537499 E
SRS21535658 dOOGlo gut metagenome samp_size 110
SRS21535658 dOOGlo gut metagenome participants_features Adult
SRS21535658 dOOGlo gut metagenome sex female
SRS21535658 dOOGlo gut metagenome age 63
SRS21535658 dOOGlo gut metagenome study_design Case-control
SRS21535658 dOOGlo gut metagenome disease Type 2 diabetic with obesity and cardiovascular complications
SRS21535658 dOOGlo gut metagenome disease_ontology_id 9352
SRS21535658 dOOGlo gut metagenome study_title Metataxonomic strategy to decipher gut bacteriome biomarkers of type 2 diabetes and obesity in Tunisian population
SRS21535658 dOOGlo gut metagenome continent Africa
SRS21535658 dOOGlo gut metagenome region North Africa
SRS21535658 dOOGlo gut metagenome assay_type Amplicon
SRS21535658 dOOGlo gut metagenome technology Illumina
SRS21535658 dOOGlo gut metagenome platform Illumina MiSeq
SRS21535658 dOOGlo gut metagenome average_spotlength 601
SRS21535658 dOOGlo gut metagenome library_layout Paired
SRS21535658 dOOGlo gut metagenome target_amplicon V3-V4
SRS21535658 dOOGlo gut metagenome submitters Meriem FASSATOUI/Abdelmajid ABID/Rym KEFI
SRS21535658 dOOGlo gut metagenome Isolate PALUd
SRS21535658 dOOGlo gut metagenome BioSampleModel Metagenome or environmental
SRS21535659 ALGip gut metagenome host Homo sapiens
SRS21535659 ALGip gut metagenome collection_date 2018
SRS21535659 ALGip gut metagenome geo_loc_name Tunisia
SRS21535659 ALGip gut metagenome lat_lon 33.886917 N 9.537499 E
SRS21535659 ALGip gut metagenome samp_size 110
SRS21535659 ALGip gut metagenome participants_features Adult
SRS21535659 ALGip gut metagenome sex female
SRS21535659 ALGip gut metagenome age 65
SRS21535659 ALGip gut metagenome study_design Case-control
SRS21535659 ALGip gut metagenome disease Type 2 diabetic with obesity and cardiovascular complications
SRS21535659 ALGip gut metagenome disease_ontology_id 9352
SRS21535659 ALGip gut metagenome study_title Metataxonomic strategy to decipher gut bacteriome biomarkers of type 2 diabetes and obesity in Tunisian population
SRS21535659 ALGip gut metagenome continent Africa
SRS21535659 ALGip gut metagenome region North Africa
SRS21535659 ALGip gut metagenome assay_type Amplicon
SRS21535659 ALGip gut metagenome technology Illumina
SRS21535659 ALGip gut metagenome platform Illumina MiSeq
SRS21535659 ALGip gut metagenome average_spotlength 601
SRS21535659 ALGip gut metagenome library_layout Paired
SRS21535659 ALGip gut metagenome target_amplicon V3-V4
SRS21535659 ALGip gut metagenome submitters Meriem FASSATOUI/Abdelmajid ABID/Rym KEFI
SRS21535659 ALGip gut metagenome Isolate prism
SRS21535659 ALGip gut metagenome BioSampleModel Metagenome or environmental

SRS21535660 ArCALM gut metagenome host Homo sapiens
SRS21535660 ArCALM gut metagenome collection_date 2018
SRS21535660 ArCALM gut metagenome geo_loc_name Tunisia
SRS21535660 ArCALM gut metagenome lat_lon 33.886917 N 9.537499 E
SRS21535660 ArCALM gut metagenome samp_size 110
SRS21535660 ArCALM gut metagenome participants_features Adult
SRS21535660 ArCALM gut metagenome sex male
SRS21535660 ArCALM gut metagenome age 50
SRS21535660 ArCALM gut metagenome study_design Case-control
SRS21535660 ArCALM gut metagenome disease Type 2 diabetic with obesity and cardiovascular complications
SRS21535660 ArCALM gut metagenome disease_ontology_id 9352
SRS21535660 ArCALM gut metagenome study_title Metataxonomic strategy to decipher gut bacteriome biomarkers of type 2 diabetes and obesity in Tunisian population
SRS21535660 ArCALM gut metagenome continent Africa
SRS21535660 ArCALM gut metagenome region North Africa
SRS21535660 ArCALM gut metagenome assay_type Amplicon
SRS21535660 ArCALM gut metagenome technology Illumina
SRS21535660 ArCALM gut metagenome platform Illumina MiSeq
SRS21535660 ArCALM gut metagenome average_spotlength 601
SRS21535660 ArCALM gut metagenome library_layout Paired
SRS21535660 ArCALM gut metagenome target_amplicon V3-V4
SRS21535660 ArCALM gut metagenome submitters Meriem FASSATOUI/Abdelmajid ABID/Rym KEFI
SRS21535660 ArCALM gut metagenome Isolate fusib
SRS21535660 ArCALM gut metagenome BioSampleModel Metagenome or environmental
SRS21535661 6KU gut metagenome host Homo sapiens
SRS21535661 6KU gut metagenome collection_date 2018
SRS21535661 6KU gut metagenome geo_loc_name Tunisia
SRS21535661 6KU gut metagenome lat_lon 33.886917 N 9.537499 E
SRS21535661 6KU gut metagenome samp_size 110
SRS21535661 6KU gut metagenome participants_features Adult
SRS21535661 6KU gut metagenome sex female
SRS21535661 6KU gut metagenome age 59
SRS21535661 6KU gut metagenome study_design Case-control
SRS21535661 6KU gut metagenome disease Non Diabetic
SRS21535661 6KU gut metagenome study_title Metataxonomic strategy to decipher gut bacteriome biomarkers of type 2 diabetes and obesity in Tunisian population
SRS21535661 6KU gut metagenome continent Africa
SRS21535661 6KU gut metagenome region North Africa
SRS21535661 6KU gut metagenome assay_type Amplicon
SRS21535661 6KU gut metagenome technology Illumina
SRS21535661 6KU gut metagenome platform Illumina MiSeq
SRS21535661 6KU gut metagenome average_spotlength 601
SRS21535661 6KU gut metagenome library_layout Paired
SRS21535661 6KU gut metagenome target_amplicon V3-V4
SRS21535661 6KU gut metagenome submitters Meriem FASSATOUI/Abdelmajid ABID/Rym KEFI
SRS21535661 6KU gut metagenome Isolate MO
SRS21535661 6KU gut metagenome BioSampleModel Metagenome or environmental
SRS21535662 TREATM gut metagenome host Homo sapiens
SRS21535662 TREATM gut metagenome collection_date 2018
SRS21535662 TREATM gut metagenome geo_loc_name Tunisia
SRS21535662 TREATM gut metagenome lat_lon 33.886917 N 9.537499 E
SRS21535662 TREATM gut metagenome samp_size 110
SRS21535662 TREATM gut metagenome participants_features Adult
SRS21535662 TREATM gut metagenome sex male
SRS21535662 TREATM gut metagenome age 51
SRS21535662 TREATM gut metagenome study_design Case-control
SRS21535662 TREATM gut metagenome disease Type 2 diabetic with obesity and cardiovascular complications
SRS21535662 TREATM gut metagenome disease_ontology_id 9352
SRS21535662 TREATM gut metagenome study_title Metataxonomic strategy to decipher gut bacteriome biomarkers of type 2 diabetes and obesity in Tunisian population
SRS21535662 TREATM gut metagenome continent Africa
SRS21535662 TREATM gut metagenome region North Africa
SRS21535662 TREATM gut metagenome assay_type Amplicon
SRS21535662 TREATM gut metagenome technology Illumina
SRS21535662 TREATM gut metagenome platform Illumina MiSeq
SRS21535662 TREATM gut metagenome average_spotlength 601
SRS21535662 TREATM gut metagenome library_layout Paired

SRS21535662 TREATM gut metagenome target_amplicon V3-V4
SRS21535662 TREATM gut metagenome submitters Meriem FASSATOUI/Abdelmajid ABID/Rym KEFI
SRS21535662 TREATM gut metagenome Isolate Atb
SRS21535662 TREATM gut metagenome BioSampleModel Metagenome or environmental
SRS21535663 VALUENVD9 gut metagenome host Homo sapiens
SRS21535663 VALUENVD9 gut metagenome collection_date 2018
SRS21535663 VALUENVD9 gut metagenome geo_loc_name Tunisia
SRS21535663 VALUENVD9 gut metagenome lat_lon 33.886917 N 9.537499 E
SRS21535663 VALUENVD9 gut metagenome samp_size 110
SRS21535663 VALUENVD9 gut metagenome participants_features Adult
SRS21535663 VALUENVD9 gut metagenome sex male
SRS21535663 VALUENVD9 gut metagenome age 63
SRS21535663 VALUENVD9 gut metagenome study_design Case-control
SRS21535663 VALUENVD9 gut metagenome disease Type 2 diabetic with obesity and cardiovascular complications
SRS21535663 VALUENVD9 gut metagenome disease_ontology_id 9352
SRS21535663 VALUENVD9 gut metagenome study_title Metataxonomic strategy to decipher gut bacteriome biomarkers of type 2 diabetes and obesity in Tunisian population
SRS21535663 VALUENVD9 gut metagenome continent Africa
SRS21535663 VALUENVD9 gut metagenome region North Africa
SRS21535663 VALUENVD9 gut metagenome assay_type Amplicon
SRS21535663 VALUENVD9 gut metagenome technology Illumina
SRS21535663 VALUENVD9 gut metagenome platform Illumina MiSeq
SRS21535663 VALUENVD9 gut metagenome average_spotlength 601
SRS21535663 VALUENVD9 gut metagenome library_layout Paired
SRS21535663 VALUENVD9 gut metagenome target_amplicon V3-V4
SRS21535663 VALUENVD9 gut metagenome submitters Meriem FASSATOUI/Abdelmajid ABID/Rym KEFI
SRS21535663 VALUENVD9 gut metagenome Isolate fongi
SRS21535663 VALUENVD9 gut metagenome BioSampleModel Metagenome or environmental
SRS21535664 WIFIaml gut metagenome host Homo sapiens
SRS21535664 WIFIaml gut metagenome collection_date 2018
SRS21535664 WIFIaml gut metagenome geo_loc_name Tunisia
SRS21535664 WIFIaml gut metagenome lat_lon 33.886917 N 9.537499 E
SRS21535664 WIFIaml gut metagenome samp_size 110
SRS21535664 WIFIaml gut metagenome participants_features Adult
SRS21535664 WIFIaml gut metagenome sex female
SRS21535664 WIFIaml gut metagenome age 62
SRS21535664 WIFIaml gut metagenome study_design Case-control
SRS21535664 WIFIaml gut metagenome disease Type 2 diabetic with obesity and cardiovascular complications
SRS21535664 WIFIaml gut metagenome disease_ontology_id 9352
SRS21535664 WIFIaml gut metagenome study_title Metataxonomic strategy to decipher gut bacteriome biomarkers of type 2 diabetes and obesity in Tunisian population
SRS21535664 WIFIaml gut metagenome continent Africa
SRS21535664 WIFIaml gut metagenome region North Africa
SRS21535664 WIFIaml gut metagenome assay_type Amplicon
SRS21535664 WIFIaml gut metagenome technology Illumina
SRS21535664 WIFIaml gut metagenome platform Illumina MiSeq
SRS21535664 WIFIaml gut metagenome average_spotlength 601
SRS21535664 WIFIaml gut metagenome library_layout Paired
SRS21535664 WIFIaml gut metagenome target_amplicon V3-V4
SRS21535664 WIFIaml gut metagenome submitters Meriem FASSATOUI/Abdelmajid ABID/Rym KEFI
SRS21535664 WIFIaml gut metagenome Isolate cartridge
SRS21535664 WIFIaml gut metagenome BioSampleModel Metagenome or environmental
SRS21535665 SOUrce gut metagenome host Homo sapiens
SRS21535665 SOUrce gut metagenome collection_date 2018
SRS21535665 SOUrce gut metagenome geo_loc_name Tunisia
SRS21535665 SOUrce gut metagenome lat_lon 33.886917 N 9.537499 E
SRS21535665 SOUrce gut metagenome samp_size 110
SRS21535665 SOUrce gut metagenome participants_features Adult
SRS21535665 SOUrce gut metagenome sex female
SRS21535665 SOUrce gut metagenome age 66
SRS21535665 SOUrce gut metagenome study_design Case-control
SRS21535665 SOUrce gut metagenome disease Type 2 diabetic with obesity and cardiovascular complications
SRS21535665 SOUrce gut metagenome disease_ontology_id 9352
SRS21535665 SOUrce gut metagenome study_title Metataxonomic strategy to decipher gut bacteriome biomarkers of type 2 diabetes and obesity in Tunisian population

SRS21535665 SOUrce gut metagenome continent Africa
SRS21535665 SOUrce gut metagenome region North Africa
SRS21535665 SOUrce gut metagenome assay_type Amplicon
SRS21535665 SOUrce gut metagenome technology Illumina
SRS21535665 SOUrce gut metagenome platform Illumina MiSeq
SRS21535665 SOUrce gut metagenome average_spotlength 601
SRS21535665 SOUrce gut metagenome library_layout Paired
SRS21535665 SOUrce gut metagenome target_amplicon V3-V4
SRS21535665 SOUrce gut metagenome submitters Meriem FASSATOUI/Abdelmajid ABID/Rym KEFI
SRS21535665 SOUrce gut metagenome Isolate grif
SRS21535665 SOUrce gut metagenome BioSampleModel Metagenome or environmental
SRS21535666 ScR95a gut metagenome host Homo sapiens
SRS21535666 ScR95a gut metagenome collection_date 2018
SRS21535666 ScR95a gut metagenome geo_loc_name Tunisia
SRS21535666 ScR95a gut metagenome lat_lon 33.886917 N 9.537499 E
SRS21535666 ScR95a gut metagenome samp_size 110
SRS21535666 ScR95a gut metagenome participants_features Adult
SRS21535666 ScR95a gut metagenome sex female
SRS21535666 ScR95a gut metagenome age 63
SRS21535666 ScR95a gut metagenome study_design Case-control
SRS21535666 ScR95a gut metagenome disease Type 2 diabetic with obesity and cardiovascular complications
SRS21535666 ScR95a gut metagenome disease_ontology_id 9352
SRS21535666 ScR95a gut metagenome study_title Metataxonomic strategy to decipher gut bacteriome biomarkers of type 2 diabetes and obesity in Tunisian population
SRS21535666 ScR95a gut metagenome continent Africa
SRS21535666 ScR95a gut metagenome region North Africa
SRS21535666 ScR95a gut metagenome assay_type Amplicon
SRS21535666 ScR95a gut metagenome technology Illumina
SRS21535666 ScR95a gut metagenome platform Illumina MiSeq
SRS21535666 ScR95a gut metagenome average_spotlength 601
SRS21535666 ScR95a gut metagenome library_layout Paired
SRS21535666 ScR95a gut metagenome target_amplicon V3-V4
SRS21535666 ScR95a gut metagenome submitters Meriem FASSATOUI/Abdelmajid ABID/Rym KEFI
SRS21535666 ScR95a gut metagenome Isolate maneg
SRS21535666 ScR95a gut metagenome BioSampleModel Metagenome or environmental
SRS21535667 HOLid gut metagenome host Homo sapiens
SRS21535667 HOLid gut metagenome collection_date 2018
SRS21535667 HOLid gut metagenome geo_loc_name Tunisia
SRS21535667 HOLid gut metagenome lat_lon 33.886917 N 9.537499 E
SRS21535667 HOLid gut metagenome samp_size 110
SRS21535667 HOLid gut metagenome participants_features Adult
SRS21535667 HOLid gut metagenome sex female
SRS21535667 HOLid gut metagenome age 76
SRS21535667 HOLid gut metagenome study_design Case-control
SRS21535667 HOLid gut metagenome disease Type 2 diabetic with obesity and cardiovascular complications
SRS21535667 HOLid gut metagenome disease_ontology_id 9352
SRS21535667 HOLid gut metagenome study_title Metataxonomic strategy to decipher gut bacteriome biomarkers of type 2 diabetes and obesity in Tunisian population
SRS21535667 HOLid gut metagenome continent Africa
SRS21535667 HOLid gut metagenome region North Africa
SRS21535667 HOLid gut metagenome assay_type Amplicon
SRS21535667 HOLid gut metagenome technology Illumina
SRS21535667 HOLid gut metagenome platform Illumina MiSeq
SRS21535667 HOLid gut metagenome average_spotlength 601
SRS21535667 HOLid gut metagenome library_layout Paired
SRS21535667 HOLid gut metagenome target_amplicon V3-V4
SRS21535667 HOLid gut metagenome submitters Meriem FASSATOUI/Abdelmajid ABID/Rym KEFI
SRS21535667 HOLid gut metagenome Isolate cof
SRS21535667 HOLid gut metagenome BioSampleModel Metagenome or environmental
SRS21535668 wsqLOS gut metagenome host Homo sapiens
SRS21535668 wsqLOS gut metagenome collection_date 2018
SRS21535668 wsqLOS gut metagenome geo_loc_name Tunisia
SRS21535668 wsqLOS gut metagenome lat_lon 33.886917 N 9.537499 E
SRS21535668 wsqLOS gut metagenome samp_size 110
SRS21535668 wsqLOS gut metagenome participants_features Adult
SRS21535668 wsqLOS gut metagenome sex male
SRS21535668 wsqLOS gut metagenome age 66

```
SRS21535668	wsqLOS	gut metagenome	study_design	Case-control
SRS21535668	wsqLOS	gut metagenome	disease	Type 2 diabetic with obesity and cardiovascular complications
SRS21535668	wsqLOS	gut metagenome	disease_ontology_id	9352
SRS21535668	wsqLOS	gut metagenome	study_title	Metataxonomic strategy to decipher gut bacteriome biomarkers of type 2 diabetes and obesity in Tunisian population
SRS21535668	wsqLOS	gut metagenome	continent	Africa
SRS21535668	wsqLOS	gut metagenome	region	North Africa
SRS21535668	wsqLOS	gut metagenome	assay_type	Amplicon
SRS21535732	HERPSimplex	gut metagenome	target_amplicon	V3-V4
SRS21535732	HERPSimplex	gut metagenome	submitters	Meriem FASSATOUI/Abdelmajid ABID/Rym KEFI
SRS21535732	HERPSimplex	gut metagenome	Isolate	FIEV
SRS21535732	HERPSimplex	gut metagenome	BioSampleModel	Metagenome or environmental
SRS21535733	DIMzest	gut metagenome	host	Homo sapiens
SRS21535733	DIMzest	gut metagenome	collection_date	2018
SRS21535733	DIMzest	gut metagenome	geo_loc_name	Tunisia
SRS21535733	DIMzest	gut metagenome	lat_lon	33.886917 N 9.537499 E
SRS21535733	DIMzest	gut metagenome	samp_size	110
SRS21535733	DIMzest	gut metagenome	participants_features	Adult
SRS21535733	DIMzest	gut metagenome	sex	male
SRS21535733	DIMzest	gut metagenome	age	57
SRS21535733	DIMzest	gut metagenome	study_design	Case-control
SRS21535733	DIMzest	gut metagenome	disease	Type 2 diabetic with obesity and cardiovascular complications
SRS21535733	DIMzest	gut metagenome	disease_ontology_id	9352
SRS21535733	DIMzest	gut metagenome	study_title	Metataxonomic strategy to decipher gut bacteriome biomarkers of type 2 diabetes and obesity in Tunisian population
SRS21535733	DIMzest	gut metagenome	continent	Africa
SRS21535733	DIMzest	gut metagenome	region	North Africa
SRS21535733	DIMzest	gut metagenome	assay_type	Amplicon
SRS21535733	DIMzest	gut metagenome	technology	Illumina
SRS21535733	DIMzest	gut metagenome	platform	Illumina MiSeq
SRS21535733	DIMzest	gut metagenome	average_spotlength	601
SRS21535733	DIMzest	gut metagenome	library_layout	Paired
SRS21535733	DIMzest	gut metagenome	target_amplicon	V3-V4
SRS21535733	DIMzest	gut metagenome	submitters	Meriem FASSATOUI/Abdelmajid ABID/Rym KEFI
SRS21535733	DIMzest	gut metagenome	Isolate	JAUNE
SRS21535733	DIMzest	gut metagenome	BioSampleModel	Metagenome or environmental
SRS21535734	BARlap	gut metagenome	host	Homo sapiens
SRS21535734	BARlap	gut metagenome	collection_date	2018
SRS21535734	BARlap	gut metagenome	geo_loc_name	Tunisia
SRS21535734	BARlap	gut metagenome	lat_lon	33.886917 N 9.537499 E
SRS21535734	BARlap	gut metagenome	samp_size	110
SRS21535734	BARlap	gut metagenome	participants_features	Adult
SRS21535734	BARlap	gut metagenome	sex	female
SRS21535734	BARlap	gut metagenome	age	57
SRS21535734	BARlap	gut metagenome	study_design	Case-control
SRS21535734	BARlap	gut metagenome	disease	Type 2 diabetic with obesity and cardiovascular complications
SRS21535734	BARlap	gut metagenome	disease_ontology_id	9352
SRS21535734	BARlap	gut metagenome	study_title	Metataxonomic strategy to decipher gut bacteriome biomarkers of type 2 diabetes and obesity in Tunisian population
SRS21535734	BARlap	gut metagenome	continent	Africa
SRS21535734	BARlap	gut metagenome	region	North Africa
SRS21535734	BARlap	gut metagenome	assay_type	Amplicon
SRS21535734	BARlap	gut metagenome	technology	Illumina
SRS21535734	BARlap	gut metagenome	platform	Illumina MiSeq
SRS21535734	BARlap	gut metagenome	average_spotlength	601
SRS21535734	BARlap	gut metagenome	library_layout	Paired
SRS21535734	BARlap	gut metagenome	target_amplicon	V3-V4
SRS21535734	BARlap	gut metagenome	submitters	Meriem FASSATOUI/Abdelmajid ABID/Rym KEFI
SRS21535734	BARlap	gut metagenome	Isolate	MENIN
SRS21535734	BARlap	gut metagenome	BioSampleModel	Metagenome or environmental
SRS21535735	TopJAR	gut metagenome	host	Homo sapiens
SRS21535735	TopJAR	gut metagenome	collection_date	2018
SRS21535735	TopJAR	gut metagenome	geo_loc_name	Tunisia
SRS21535735	TopJAR	gut metagenome	lat_lon	33.886917 N 9.537499 E
SRS21535735	TopJAR	gut metagenome	samp_size	110
```

SRS21535735 TopJAR gut metagenome participants_features Adult
SRS21535735 TopJAR gut metagenome sex male
SRS21535735 TopJAR gut metagenome age 72
SRS21535735 TopJAR gut metagenome study_design Case-control
SRS21535735 TopJAR gut metagenome disease Type 2 diabetic with obesity and cardiovascular complications
SRS21535735 TopJAR gut metagenome disease_ontology_id 9352
SRS21535735 TopJAR gut metagenome study_title Metataxonomic strategy to decipher gut bacteriome biomarkers of type 2 diabetes and obesity in Tunisian population
SRS21535735 TopJAR gut metagenome continent Africa
SRS21535735 TopJAR gut metagenome region North Africa
SRS21535735 TopJAR gut metagenome assay_type Amplicon
SRS21535735 TopJAR gut metagenome technology Illumina
SRS21535735 TopJAR gut metagenome platform Illumina MiSeq
SRS21535735 TopJAR gut metagenome average_spotlength 601
SRS21535735 TopJAR gut metagenome library_layout Paired
SRS21535735 TopJAR gut metagenome target_amplicon V3-V4
SRS21535735 TopJAR gut metagenome submitters Meriem FASSATOUI/Abdelmajid ABID/Rym KEFI
SRS21535735 TopJAR gut metagenome Isolate MALAR
SRS21535735 TopJAR gut metagenome BioSampleModel Metagenome or environmental
SRS21535668 wsqLOS gut metagenome technology Illumina
SRS21535668 wsqLOS gut metagenome platform Illumina MiSeq
SRS21535668 wsqLOS gut metagenome average_spotlength 601
SRS21535668 wsqLOS gut metagenome library_layout Paired
SRS21535668 wsqLOS gut metagenome target_amplicon V3-V4
SRS21535668 wsqLOS gut metagenome submitters Meriem FASSATOUI/Abdelmajid ABID/Rym KEFI
SRS21535668 wsqLOS gut metagenome Isolate polya
SRS21535668 wsqLOS gut metagenome BioSampleModel Metagenome or environmental
SRS21535669 Tcytop gut metagenome host Homo sapiens
SRS21535669 Tcytop gut metagenome collection_date 2018
SRS21535669 Tcytop gut metagenome geo_loc_name Tunisia
SRS21535669 Tcytop gut metagenome lat_lon 33.886917 N 9.537499 E
SRS21535669 Tcytop gut metagenome samp_size 110
SRS21535669 Tcytop gut metagenome participants_features Adult
SRS21535669 Tcytop gut metagenome sex female
SRS21535669 Tcytop gut metagenome age 50
SRS21535669 Tcytop gut metagenome study_design Case-control
SRS21535669 Tcytop gut metagenome disease Type 2 diabetic with obesity and cardiovascular complications
SRS21535669 Tcytop gut metagenome disease_ontology_id 9352
SRS21535669 Tcytop gut metagenome study_title Metataxonomic strategy to decipher gut bacteriome biomarkers of type 2 diabetes and obesity in Tunisian population
SRS21535669 Tcytop gut metagenome continent Africa
SRS21535669 Tcytop gut metagenome region North Africa
SRS21535669 Tcytop gut metagenome assay_type Amplicon
SRS21535669 Tcytop gut metagenome technology Illumina
SRS21535669 Tcytop gut metagenome platform Illumina MiSeq
SRS21535669 Tcytop gut metagenome average_spotlength 601
SRS21535669 Tcytop gut metagenome library_layout Paired
SRS21535669 Tcytop gut metagenome target_amplicon V3-V4
SRS21535669 Tcytop gut metagenome submitters Meriem FASSATOUI/Abdelmajid ABID/Rym KEFI
SRS21535669 Tcytop gut metagenome Isolate dntp
SRS21535669 Tcytop gut metagenome BioSampleModel Metagenome or environmental
SRS21535670 BONEnarrow gut metagenome host Homo sapiens
SRS21535670 BONEnarrow gut metagenome collection_date 2018
SRS21535670 BONEnarrow gut metagenome geo_loc_name Tunisia
SRS21535670 BONEnarrow gut metagenome lat_lon 33.886917 N 9.537499 E
SRS21535670 BONEnarrow gut metagenome samp_size 110
SRS21535670 BONEnarrow gut metagenome participants_features Adult
SRS21535670 BONEnarrow gut metagenome sex female
SRS21535670 BONEnarrow gut metagenome age 57
SRS21535670 BONEnarrow gut metagenome study_design Case-control
SRS21535670 BONEnarrow gut metagenome disease Type 2 diabetic with obesity and cardiovascular complications
SRS21535670 BONEnarrow gut metagenome disease_ontology_id 9352
SRS21535670 BONEnarrow gut metagenome study_title Metataxonomic strategy to decipher gut bacteriome biomarkers of type 2 diabetes and obesity in Tunisian population
SRS21535670 BONEnarrow gut metagenome continent Africa
SRS21535670 BONEnarrow gut metagenome region North Africa

SRS21535670 BONEnarrow gut metagenome assay_type Amplicon
SRS21535670 BONEnarrow gut metagenome technology Illumina
SRS21535670 BONEnarrow gut metagenome platform Illumina MiSeq
SRS21535670 BONEnarrow gut metagenome average_spotlength 601
SRS21535670 BONEnarrow gut metagenome library_layout Paired
SRS21535670 BONEnarrow gut metagenome target_amplicon V3-V4
SRS21535670 BONEnarrow gut metagenome submitters Meriem FASSATOUI/Abdelmajid ABID/Rym KEFI
SRS21535670 BONEnarrow gut metagenome Isolate mgcl
SRS21535670 BONEnarrow gut metagenome BioSampleModel Metagenome or environmental
SRS21535671 EBV gut metagenome host Homo sapiens
SRS21535671 EBV gut metagenome collection_date 2018
SRS21535671 EBV gut metagenome geo_loc_name Tunisia
SRS21535671 EBV gut metagenome lat_lon 33.886917 N 9.537499 E
SRS21535671 EBV gut metagenome samp_size 110
SRS21535671 EBV gut metagenome participants_features Adult
SRS21535671 EBV gut metagenome sex female
SRS21535671 EBV gut metagenome age 66
SRS21535671 EBV gut metagenome study_design Case-control
SRS21535671 EBV gut metagenome disease Type 2 diabetic with obesity and cardiovascular complications
SRS21535671 EBV gut metagenome disease_ontology_id 9352
SRS21535671 EBV gut metagenome study_title Metataxonomic strategy to decipher gut bacteriome biomarkers of type 2 diabetes and obesity in Tunisian population
SRS21535671 EBV gut metagenome continent Africa
SRS21535671 EBV gut metagenome region North Africa
SRS21535671 EBV gut metagenome assay_type Amplicon
SRS21535671 EBV gut metagenome technology Illumina
SRS21535671 EBV gut metagenome platform Illumina MiSeq
SRS21535671 EBV gut metagenome average_spotlength 601
SRS21535671 EBV gut metagenome library_layout Paired
SRS21535671 EBV gut metagenome target_amplicon V3-V4
SRS21535671 EBV gut metagenome submitters Meriem FASSATOUI/Abdelmajid ABID/Rym KEFI
SRS21535671 EBV gut metagenome Isolate prime
SRS21535671 EBV gut metagenome BioSampleModel Metagenome or environmental
SRS21535672 JO gut metagenome host Homo sapiens
SRS21535672 JO gut metagenome collection_date 2018
SRS21535672 JO gut metagenome geo_loc_name Tunisia
SRS21535672 JO gut metagenome lat_lon 33.886917 N 9.537499 E
SRS21535672 JO gut metagenome samp_size 110
SRS21535672 JO gut metagenome participants_features Adult
SRS21535672 JO gut metagenome sex male
SRS21535672 JO gut metagenome age 64
SRS21535672 JO gut metagenome study_design Case-control
SRS21535672 JO gut metagenome disease Non Diabetic
SRS21535672 JO gut metagenome study_title Metataxonomic strategy to decipher gut bacteriome biomarkers of type 2 diabetes and obesity in Tunisian population
SRS21535672 JO gut metagenome continent Africa
SRS21535672 JO gut metagenome region North Africa
SRS21535672 JO gut metagenome assay_type Amplicon
SRS21535672 JO gut metagenome technology Illumina
SRS21535672 JO gut metagenome platform Illumina MiSeq
SRS21535672 JO gut metagenome average_spotlength 601
SRS21535672 JO gut metagenome library_layout Paired
SRS21535672 JO gut metagenome target_amplicon V3-V4
SRS21535672 JO gut metagenome submitters Meriem FASSATOUI/Abdelmajid ABID/Rym KEFI
SRS21535672 JO gut metagenome Isolate LIGHT
SRS21535672 JO gut metagenome BioSampleModel Metagenome or environmental
SRS21535673 DROMA3 gut metagenome host Homo sapiens
SRS21535673 DROMA3 gut metagenome collection_date 2018
SRS21535673 DROMA3 gut metagenome geo_loc_name Tunisia
SRS21535673 DROMA3 gut metagenome lat_lon 33.886917 N 9.537499 E
SRS21535673 DROMA3 gut metagenome samp_size 110
SRS21535673 DROMA3 gut metagenome participants_features Adult
SRS21535673 DROMA3 gut metagenome sex female
SRS21535673 DROMA3 gut metagenome age 40
SRS21535673 DROMA3 gut metagenome study_design Case-control
SRS21535673 DROMA3 gut metagenome disease Non Diabetic
SRS21535673 DROMA3 gut metagenome study_title Metataxonomic strategy to decipher gut

```
bacteriome biomarkers of type 2 diabetes and obesity in Tunisian population
SRS21535673	DROMA3	gut metagenome	continent	Africa
SRS21535673	DROMA3	gut metagenome	region	North Africa
SRS21535673	DROMA3	gut metagenome	assay_type	Amplicon
SRS21535673	DROMA3	gut metagenome	technology	Illumina
SRS21535673	DROMA3	gut metagenome	platform	Illumina MiSeq
SRS21535673	DROMA3	gut metagenome	average_spotlength	601
SRS21535673	DROMA3	gut metagenome	library_layout	Paired
SRS21535673	DROMA3	gut metagenome	target_amplicon V3-V4
SRS21535673	DROMA3	gut metagenome	submitters	Meriem FASSATOUI/Abdelmajid ABID/Rym KEFI
SRS21535673	DROMA3	gut metagenome	Isolate gly
SRS21535673	DROMA3	gut metagenome	BioSampleModel	Metagenome or environmental
SRS21535674	Q9SA	gut metagenome	host	Homo sapiens
SRS21535674	Q9SA	gut metagenome	collection_date 2018
SRS21535674	Q9SA	gut metagenome	geo_loc_name	Tunisia
SRS21535674	Q9SA	gut metagenome	lat_lon 33.886917 N 9.537499 E
SRS21535674	Q9SA	gut metagenome	samp_size	110
SRS21535674	Q9SA	gut metagenome	participants_features	Adult
SRS21535674	Q9SA	gut metagenome	sex	female
SRS21535674	Q9SA	gut metagenome	age	58
SRS21535674	Q9SA	gut metagenome	study_design	Case-control
SRS21535674	Q9SA	gut metagenome	disease Non Diabetic
SRS21535674	Q9SA	gut metagenome	study_title	Metataxonomic strategy to decipher gut
bacteriome biomarkers of type 2 diabetes and obesity in Tunisian population
SRS21535674	Q9SA	gut metagenome	continent	Africa
SRS21535674	Q9SA	gut metagenome	region	North Africa
SRS21535674	Q9SA	gut metagenome	assay_type	Amplicon
SRS21535674	Q9SA	gut metagenome	technology	Illumina
SRS21535674	Q9SA	gut metagenome	platform	Illumina MiSeq
SRS21535674	Q9SA	gut metagenome	average_spotlength	601
SRS21535674	Q9SA	gut metagenome	library_layout	Paired
SRS21535674	Q9SA	gut metagenome	target_amplicon V3-V4
SRS21535674	Q9SA	gut metagenome	submitters	Meriem FASSATOUI/Abdelmajid ABID/Rym KEFI
SRS21535674	Q9SA	gut metagenome	Isolate PROT
SRS21535674	Q9SA	gut metagenome	BioSampleModel	Metagenome or environmental
SRS21535675	ADC	gut metagenome	host	Homo sapiens
SRS21535675	ADC	gut metagenome	collection_date 2018
SRS21535675	ADC	gut metagenome	geo_loc_name	Tunisia
SRS21535675	ADC	gut metagenome	lat_lon 33.886917 N 9.537499 E
SRS21535675	ADC	gut metagenome	samp_size	110
SRS21535675	ADC	gut metagenome	participants_features	Adult
SRS21535675	ADC	gut metagenome	sex	female
SRS21535675	ADC	gut metagenome	age	46
SRS21535675	ADC	gut metagenome	study_design	Case-control
SRS21535675	ADC	gut metagenome	disease Non Diabetic
SRS21535675	ADC	gut metagenome	study_title	Metataxonomic strategy to decipher gut
bacteriome biomarkers of type 2 diabetes and obesity in Tunisian population
SRS21535675	ADC	gut metagenome	continent	Africa
SRS21535675	ADC	gut metagenome	region	North Africa
SRS21535675	ADC	gut metagenome	assay_type	Amplicon
SRS21535675	ADC	gut metagenome	technology	Illumina
SRS21535675	ADC	gut metagenome	platform	Illumina MiSeq
SRS21535675	ADC	gut metagenome	average_spotlength	601
SRS21535675	ADC	gut metagenome	library_layout	Paired
SRS21535675	ADC	gut metagenome	target_amplicon V3-V4
SRS21535675	ADC	gut metagenome	submitters	Meriem FASSATOUI/Abdelmajid ABID/Rym KEFI
SRS21535675	ADC	gut metagenome	Isolate CREA
SRS21535675	ADC	gut metagenome	BioSampleModel	Metagenome or environmental
SRS21535676	96Hz	gut metagenome	host	Homo sapiens
SRS21535676	96Hz	gut metagenome	collection_date 2018
SRS21535676	96Hz	gut metagenome	geo_loc_name	Tunisia
SRS21535676	96Hz	gut metagenome	lat_lon 33.886917 N 9.537499 E
SRS21535676	96Hz	gut metagenome	samp_size	110
SRS21535676	96Hz	gut metagenome	participants_features	Adult
SRS21535676	96Hz	gut metagenome	sex	female
SRS21535676	96Hz	gut metagenome	age	48
SRS21535676	96Hz	gut metagenome	study_design	Case-control
SRS21535676	96Hz	gut metagenome	disease Non Diabetic
SRS21535676	96Hz	gut metagenome	study_title	Metataxonomic strategy to decipher gut
```

```
bacteriome biomarkers of type 2 diabetes and obesity in Tunisian population
SRS21535676	96Hz	gut metagenome	continent	Africa
SRS21535676	96Hz	gut metagenome	region	North Africa
SRS21535676	96Hz	gut metagenome	assay_type	Amplicon
SRS21535676	96Hz	gut metagenome	technology	Illumina
SRS21535676	96Hz	gut metagenome	platform	Illumina MiSeq
SRS21535676	96Hz	gut metagenome	average_spotlength	601
SRS21535676	96Hz	gut metagenome	library_layout	Paired
SRS21535676	96Hz	gut metagenome	target_amplicon	V3-V4
SRS21535676	96Hz	gut metagenome	submitters	Meriem FASSATOUI/Abdelmajid ABID/Rym KEFI
SRS21535676	96Hz	gut metagenome	Isolate	NFS
SRS21535676	96Hz	gut metagenome	BioSampleModel	Metagenome or environmental
SRS21535677	VHSME	gut metagenome	host	Homo sapiens
SRS21535677	VHSME	gut metagenome	collection_date	2018
SRS21535677	VHSME	gut metagenome	geo_loc_name	Tunisia
SRS21535677	VHSME	gut metagenome	lat_lon	33.886917 N 9.537499 E
SRS21535677	VHSME	gut metagenome	samp_size	110
SRS21535677	VHSME	gut metagenome	participants_features	Adult
SRS21535677	VHSME	gut metagenome	sex	female
SRS21535677	VHSME	gut metagenome	age	46
SRS21535677	VHSME	gut metagenome	study_design	Case-control
SRS21535677	VHSME	gut metagenome	disease	Non Diabetic
SRS21535677	VHSME	gut metagenome	study_title	Metataxonomic strategy to decipher gut
bacteriome biomarkers of type 2 diabetes and obesity in Tunisian population
SRS21535677	VHSME	gut metagenome	continent	Africa
SRS21535677	VHSME	gut metagenome	region	North Africa
SRS21535677	VHSME	gut metagenome	assay_type	Amplicon
SRS21535677	VHSME	gut metagenome	technology	Illumina
SRS21535677	VHSME	gut metagenome	platform	Illumina MiSeq
SRS21535677	VHSME	gut metagenome	average_spotlength	601
SRS21535677	VHSME	gut metagenome	library_layout	Paired
SRS21535677	VHSME	gut metagenome	target_amplicon	V3-V4
SRS21535677	VHSME	gut metagenome	submitters	Meriem FASSATOUI/Abdelmajid ABID/Rym KEFI
SRS21535677	VHSME	gut metagenome	Isolate	PLAQ
SRS21535677	VHSME	gut metagenome	BioSampleModel	Metagenome or environmental
SRS21535678	VEYU	gut metagenome	host	Homo sapiens
SRS21535678	VEYU	gut metagenome	collection_date	2018
SRS21535678	VEYU	gut metagenome	geo_loc_name	Tunisia
SRS21535678	VEYU	gut metagenome	lat_lon	33.886917 N 9.537499 E
SRS21535678	VEYU	gut metagenome	samp_size	110
SRS21535678	VEYU	gut metagenome	participants_features	Adult
SRS21535678	VEYU	gut metagenome	sex	male
SRS21535678	VEYU	gut metagenome	age	46
SRS21535678	VEYU	gut metagenome	study_design	Case-control
SRS21535678	VEYU	gut metagenome	disease	Non Diabetic
SRS21535678	VEYU	gut metagenome	study_title	Metataxonomic strategy to decipher gut
bacteriome biomarkers of type 2 diabetes and obesity in Tunisian population
SRS21535678	VEYU	gut metagenome	continent	Africa
SRS21535678	VEYU	gut metagenome	region	North Africa
SRS21535678	VEYU	gut metagenome	assay_type	Amplicon
SRS21535678	VEYU	gut metagenome	technology	Illumina
SRS21535678	VEYU	gut metagenome	platform	Illumina MiSeq
SRS21535678	VEYU	gut metagenome	average_spotlength	601
SRS21535678	VEYU	gut metagenome	library_layout	Paired
SRS21535678	VEYU	gut metagenome	target_amplicon	V3-V4
SRS21535678	VEYU	gut metagenome	submitters	Meriem FASSATOUI/Abdelmajid ABID/Rym KEFI
SRS21535678	VEYU	gut metagenome	Isolate	TIME
SRS21535678	VEYU	gut metagenome	BioSampleModel	Metagenome or environmental
SRS21535679	EFORT	gut metagenome	host	Homo sapiens
SRS21535679	EFORT	gut metagenome	collection_date	2018
SRS21535679	EFORT	gut metagenome	geo_loc_name	Tunisia
SRS21535679	EFORT	gut metagenome	lat_lon	33.886917 N 9.537499 E
SRS21535679	EFORT	gut metagenome	samp_size	110
SRS21535679	EFORT	gut metagenome	participants_features	Adult
SRS21535679	EFORT	gut metagenome	sex	male
SRS21535679	EFORT	gut metagenome	age	71
SRS21535679	EFORT	gut metagenome	study_design	Case-control
SRS21535679	EFORT	gut metagenome	disease	Non Diabetic
SRS21535679	EFORT	gut metagenome	study_title	Metataxonomic strategy to decipher gut
```

bacteriome biomarkers of type 2 diabetes and obesity in Tunisian population
SRS21535679 EFORT gut metagenome continent Africa
SRS21535679 EFORT gut metagenome region North Africa
SRS21535679 EFORT gut metagenome assay_type Amplicon
SRS21535679 EFORT gut metagenome technology Illumina
SRS21535679 EFORT gut metagenome platform Illumina MiSeq
SRS21535679 EFORT gut metagenome average_spotlength 601
SRS21535679 EFORT gut metagenome library_layout Paired
SRS21535679 EFORT gut metagenome target_amplicon V3-V4
SRS21535679 EFORT gut metagenome submitters Meriem FASSATOUI/Abdelmajid ABID/Rym KEFI
SRS21535679 EFORT gut metagenome Isolate GLYC
SRS21535679 EFORT gut metagenome BioSampleModel Metagenome or environmental
SRS21535680 TULEAD gut metagenome host Homo sapiens
SRS21535680 TULEAD gut metagenome collection_date 2018
SRS21535680 TULEAD gut metagenome geo_loc_name Tunisia
SRS21535680 TULEAD gut metagenome lat_lon 33.886917 N 9.537499 E
SRS21535680 TULEAD gut metagenome samp_size 110
SRS21535680 TULEAD gut metagenome participants_features Adult
SRS21535680 TULEAD gut metagenome sex female
SRS21535680 TULEAD gut metagenome age 59
SRS21535680 TULEAD gut metagenome study_design Case-control
SRS21535680 TULEAD gut metagenome disease Non Diabetic
SRS21535680 TULEAD gut metagenome study_title Metataxonomic strategy to decipher gut
bacteriome biomarkers of type 2 diabetes and obesity in Tunisian population
SRS21535680 TULEAD gut metagenome continent Africa
SRS21535680 TULEAD gut metagenome region North Africa
SRS21535680 TULEAD gut metagenome assay_type Amplicon
SRS21535680 TULEAD gut metagenome technology Illumina
SRS21535680 TULEAD gut metagenome platform Illumina MiSeq
SRS21535680 TULEAD gut metagenome average_spotlength 601
SRS21535680 TULEAD gut metagenome library_layout Paired
SRS21535680 TULEAD gut metagenome target_amplicon V3-V4
SRS21535680 TULEAD gut metagenome submitters Meriem FASSATOUI/Abdelmajid ABID/Rym KEFI
SRS21535680 TULEAD gut metagenome Isolate BILIRUB
SRS21535680 TULEAD gut metagenome BioSampleModel Metagenome or environmental
SRS21535681 yALTOP gut metagenome host Homo sapiens
SRS21535681 yALTOP gut metagenome collection_date 2018
SRS21535681 yALTOP gut metagenome geo_loc_name Tunisia
SRS21535681 yALTOP gut metagenome lat_lon 33.886917 N 9.537499 E
SRS21535681 yALTOP gut metagenome samp_size 110
SRS21535681 yALTOP gut metagenome participants_features Adult
SRS21535681 yALTOP gut metagenome sex male
SRS21535681 yALTOP gut metagenome age 67
SRS21535681 yALTOP gut metagenome study_design Case-control
SRS21535681 yALTOP gut metagenome disease Non Diabetic
SRS21535681 yALTOP gut metagenome study_title Metataxonomic strategy to decipher gut
bacteriome biomarkers of type 2 diabetes and obesity in Tunisian population
SRS21535681 yALTOP gut metagenome continent Africa
SRS21535681 yALTOP gut metagenome region North Africa
SRS21535681 yALTOP gut metagenome assay_type Amplicon
SRS21535681 yALTOP gut metagenome technology Illumina
SRS21535681 yALTOP gut metagenome platform Illumina MiSeq
SRS21535681 yALTOP gut metagenome average_spotlength 601
SRS21535681 yALTOP gut metagenome library_layout Paired
SRS21535681 yALTOP gut metagenome target_amplicon V3-V4
SRS21535681 yALTOP gut metagenome submitters Meriem FASSATOUI/Abdelmajid ABID/Rym KEFI
SRS21535681 yALTOP gut metagenome Isolate CHOL
SRS21535681 yALTOP gut metagenome BioSampleModel Metagenome or environmental
SRS21535682 CYASOU gut metagenome host Homo sapiens
SRS21535682 CYASOU gut metagenome collection_date 2018
SRS21535682 CYASOU gut metagenome geo_loc_name Tunisia
SRS21535682 CYASOU gut metagenome lat_lon 33.886917 N 9.537499 E
SRS21535682 CYASOU gut metagenome samp_size 110
SRS21535682 CYASOU gut metagenome participants_features Adult
SRS21535682 CYASOU gut metagenome sex female
SRS21535682 CYASOU gut metagenome age 49
SRS21535682 CYASOU gut metagenome study_design Case-control
SRS21535682 CYASOU gut metagenome disease Non Diabetic
SRS21535682 CYASOU gut metagenome study_title Metataxonomic strategy to decipher gut

bacteriome biomarkers of type 2 diabetes and obesity in Tunisian population
SRS21535682 CYASOU gut metagenome continent Africa
SRS21535682 CYASOU gut metagenome region North Africa
SRS21535682 CYASOU gut metagenome assay_type Amplicon
SRS21535682 CYASOU gut metagenome technology Illumina
SRS21535682 CYASOU gut metagenome platform Illumina MiSeq
SRS21535682 CYASOU gut metagenome average_spotlength 601
SRS21535682 CYASOU gut metagenome library_layout Paired
SRS21535682 CYASOU gut metagenome target_amplicon V3-V4
SRS21535682 CYASOU gut metagenome submitters Meriem FASSATOUI/Abdelmajid ABID/Rym KEFI
SRS21535682 CYASOU gut metagenome Isolate HDL
SRS21535682 CYASOU gut metagenome BioSampleModel Metagenome or environmental
SRS21535683 NOvz65 gut metagenome host Homo sapiens
SRS21535683 NOvz65 gut metagenome collection_date 2018
SRS21535683 NOvz65 gut metagenome geo_loc_name Tunisia
SRS21535683 NOvz65 gut metagenome lat_lon 33.886917 N 9.537499 E
SRS21535683 NOvz65 gut metagenome samp_size 110
SRS21535683 NOvz65 gut metagenome participants_features Adult
SRS21535683 NOvz65 gut metagenome sex female
SRS21535683 NOvz65 gut metagenome age 38
SRS21535683 NOvz65 gut metagenome study_design Case-control
SRS21535683 NOvz65 gut metagenome disease Non Diabetic
SRS21535683 NOvz65 gut metagenome study_title Metataxonomic strategy to decipher gut
bacteriome biomarkers of type 2 diabetes and obesity in Tunisian population
SRS21535683 NOvz65 gut metagenome continent Africa
SRS21535683 NOvz65 gut metagenome region North Africa
SRS21535683 NOvz65 gut metagenome assay_type Amplicon
SRS21535683 NOvz65 gut metagenome technology Illumina
SRS21535683 NOvz65 gut metagenome platform Illumina MiSeq
SRS21535683 NOvz65 gut metagenome average_spotlength 601
SRS21535683 NOvz65 gut metagenome library_layout Paired
SRS21535683 NOvz65 gut metagenome target_amplicon V3-V4
SRS21535683 NOvz65 gut metagenome submitters Meriem FASSATOUI/Abdelmajid ABID/Rym KEFI
SRS21535683 NOvz65 gut metagenome Isolate LDL
SRS21535683 NOvz65 gut metagenome BioSampleModel Metagenome or environmental
SRS21535684 vAS7 gut metagenome host Homo sapiens
SRS21535684 vAS7 gut metagenome collection_date 2018
SRS21535684 vAS7 gut metagenome geo_loc_name Tunisia
SRS21535684 vAS7 gut metagenome lat_lon 33.886917 N 9.537499 E
SRS21535684 vAS7 gut metagenome samp_size 110
SRS21535684 vAS7 gut metagenome participants_features Adult
SRS21535684 vAS7 gut metagenome sex female
SRS21535684 vAS7 gut metagenome age 45
SRS21535684 vAS7 gut metagenome study_design Case-control
SRS21535684 vAS7 gut metagenome disease Non Diabetic
SRS21535684 vAS7 gut metagenome study_title Metataxonomic strategy to decipher gut
bacteriome biomarkers of type 2 diabetes and obesity in Tunisian population
SRS21535684 vAS7 gut metagenome continent Africa
SRS21535684 vAS7 gut metagenome region North Africa
SRS21535684 vAS7 gut metagenome assay_type Amplicon
SRS21535684 vAS7 gut metagenome technology Illumina
SRS21535684 vAS7 gut metagenome platform Illumina MiSeq
SRS21535684 vAS7 gut metagenome average_spotlength 601
SRS21535684 vAS7 gut metagenome library_layout Paired
SRS21535684 vAS7 gut metagenome target_amplicon V3-V4
SRS21535684 vAS7 gut metagenome submitters Meriem FASSATOUI/Abdelmajid ABID/Rym KEFI
SRS21535684 vAS7 gut metagenome Isolate cos
SRS21535684 vAS7 gut metagenome BioSampleModel Metagenome or environmental
SRS21535685 SIJR gut metagenome host Homo sapiens
SRS21535685 SIJR gut metagenome collection_date 2018
SRS21535685 SIJR gut metagenome geo_loc_name Tunisia
SRS21535685 SIJR gut metagenome lat_lon 33.886917 N 9.537499 E
SRS21535685 SIJR gut metagenome samp_size 110
SRS21535685 SIJR gut metagenome participants_features Adult
SRS21535685 SIJR gut metagenome sex male
SRS21535685 SIJR gut metagenome age 42
SRS21535685 SIJR gut metagenome study_design Case-control
SRS21535685 SIJR gut metagenome disease Non Diabetic
SRS21535685 SIJR gut metagenome study_title Metataxonomic strategy to decipher gut

```
bacteriome biomarkers of type 2 diabetes and obesity in Tunisian population
SRS21535685	SIJR	gut metagenome	continent	Africa
SRS21535685	SIJR	gut metagenome	region	North Africa
SRS21535685	SIJR	gut metagenome	assay_type	Amplicon
SRS21535685	SIJR	gut metagenome	technology	Illumina
SRS21535685	SIJR	gut metagenome	platform	Illumina MiSeq
SRS21535685	SIJR	gut metagenome	average_spotlength	601
SRS21535685	SIJR	gut metagenome	library_layout	Paired
SRS21535685	SIJR	gut metagenome	target_amplicon	V3-V4
SRS21535685	SIJR	gut metagenome	submitters	Meriem FASSATOUI/Abdelmajid ABID/Rym KEFI
SRS21535685	SIJR	gut metagenome	Isolate	TG
SRS21535685	SIJR	gut metagenome	BioSampleModel	Metagenome or environmental
SRS21535686	PHOCY	gut metagenome	host	Homo sapiens
SRS21535686	PHOCY	gut metagenome	collection_date	2018
SRS21535686	PHOCY	gut metagenome	geo_loc_name	Tunisia
SRS21535686	PHOCY	gut metagenome	lat_lon	33.886917 N 9.537499 E
SRS21535686	PHOCY	gut metagenome	samp_size	110
SRS21535686	PHOCY	gut metagenome	participants_features	Adult
SRS21535686	PHOCY	gut metagenome	sex	male
SRS21535686	PHOCY	gut metagenome	age	43
SRS21535686	PHOCY	gut metagenome	study_design	Case-control
SRS21535686	PHOCY	gut metagenome	disease	Non Diabetic
SRS21535686	PHOCY	gut metagenome	study_title	Metataxonomic strategy to decipher gut
bacteriome biomarkers of type 2 diabetes and obesity in Tunisian population
SRS21535686	PHOCY	gut metagenome	continent	Africa
SRS21535686	PHOCY	gut metagenome	region	North Africa
SRS21535686	PHOCY	gut metagenome	assay_type	Amplicon
SRS21535686	PHOCY	gut metagenome	technology	Illumina
SRS21535686	PHOCY	gut metagenome	platform	Illumina MiSeq
SRS21535686	PHOCY	gut metagenome	average_spotlength	601
SRS21535686	PHOCY	gut metagenome	library_layout	Paired
SRS21535686	PHOCY	gut metagenome	target_amplicon	V3-V4
SRS21535686	PHOCY	gut metagenome	submitters	Meriem FASSATOUI/Abdelmajid ABID/Rym KEFI
SRS21535686	PHOCY	gut metagenome	Isolate	PHADIA
SRS21535686	PHOCY	gut metagenome	BioSampleModel	Metagenome or environmental
SRS21535687	GOVUA	gut metagenome	host	Homo sapiens
SRS21535687	GOVUA	gut metagenome	collection_date	2018
SRS21535687	GOVUA	gut metagenome	geo_loc_name	Tunisia
SRS21535687	GOVUA	gut metagenome	lat_lon	33.886917 N 9.537499 E
SRS21535687	GOVUA	gut metagenome	samp_size	110
SRS21535687	GOVUA	gut metagenome	participants_features	Adult
SRS21535687	GOVUA	gut metagenome	sex	male
SRS21535687	GOVUA	gut metagenome	age	69
SRS21535687	GOVUA	gut metagenome	study_design	Case-control
SRS21535687	GOVUA	gut metagenome	disease	Non Diabetic
SRS21535687	GOVUA	gut metagenome	study_title	Metataxonomic strategy to decipher gut
bacteriome biomarkers of type 2 diabetes and obesity in Tunisian population
SRS21535687	GOVUA	gut metagenome	continent	Africa
SRS21535687	GOVUA	gut metagenome	region	North Africa
SRS21535687	GOVUA	gut metagenome	assay_type	Amplicon
SRS21535687	GOVUA	gut metagenome	technology	Illumina
SRS21535687	GOVUA	gut metagenome	platform	Illumina MiSeq
SRS21535687	GOVUA	gut metagenome	average_spotlength	601
SRS21535687	GOVUA	gut metagenome	library_layout	Paired
SRS21535687	GOVUA	gut metagenome	target_amplicon	V3-V4
SRS21535687	GOVUA	gut metagenome	submitters	Meriem FASSATOUI/Abdelmajid ABID/Rym KEFI
SRS21535687	GOVUA	gut metagenome	Isolate	TOP
SRS21535687	GOVUA	gut metagenome	BioSampleModel	Metagenome or environmental
SRS21535688	PSTUM	gut metagenome	host	Homo sapiens
SRS21535688	PSTUM	gut metagenome	collection_date	2018
SRS21535688	PSTUM	gut metagenome	geo_loc_name	Tunisia
SRS21535688	PSTUM	gut metagenome	lat_lon	33.886917 N 9.537499 E
SRS21535688	PSTUM	gut metagenome	samp_size	110
SRS21535688	PSTUM	gut metagenome	participants_features	Adult
SRS21535688	PSTUM	gut metagenome	sex	female
SRS21535688	PSTUM	gut metagenome	age	60
SRS21535688	PSTUM	gut metagenome	study_design	Case-control
SRS21535688	PSTUM	gut metagenome	disease	Non Diabetic
SRS21535688	PSTUM	gut metagenome	study_title	Metataxonomic strategy to decipher gut
```

```
bacteriome biomarkers of type 2 diabetes and obesity in Tunisian population
SRS21535688	PSTUM	gut metagenome	continent	Africa
SRS21535688	PSTUM	gut metagenome	region	North Africa
SRS21535688	PSTUM	gut metagenome	assay_type	Amplicon
SRS21535688	PSTUM	gut metagenome	technology	Illumina
SRS21535688	PSTUM	gut metagenome	platform	Illumina MiSeq
SRS21535688	PSTUM	gut metagenome	average_spotlength	601
SRS21535688	PSTUM	gut metagenome	library_layout	Paired
SRS21535688	PSTUM	gut metagenome	target_amplicon	V3-V4
SRS21535688	PSTUM	gut metagenome	submitters	Meriem FASSATOUI/Abdelmajid ABID/Rym KEFI
SRS21535688	PSTUM	gut metagenome	Isolate	ELCTRO
SRS21535688	PSTUM	gut metagenome	BioSampleModel	Metagenome or environmental
SRS21535689	WGNEA	gut metagenome	host	Homo sapiens
SRS21535689	WGNEA	gut metagenome	collection_date	2018
SRS21535689	WGNEA	gut metagenome	geo_loc_name	Tunisia
SRS21535689	WGNEA	gut metagenome	lat_lon	33.886917 N 9.537499 E
SRS21535689	WGNEA	gut metagenome	samp_size	110
SRS21535689	WGNEA	gut metagenome	participants_features	Adult
SRS21535689	WGNEA	gut metagenome	sex	female
SRS21535689	WGNEA	gut metagenome	age	60
SRS21535689	WGNEA	gut metagenome	study_design	Case-control
SRS21535689	WGNEA	gut metagenome	disease	Non Diabetic
SRS21535689	WGNEA	gut metagenome	study_title	Metataxonomic strategy to decipher gut
bacteriome biomarkers of type 2 diabetes and obesity in Tunisian population
SRS21535689	WGNEA	gut metagenome	continent	Africa
SRS21535689	WGNEA	gut metagenome	region	North Africa
SRS21535689	WGNEA	gut metagenome	assay_type	Amplicon
SRS21535689	WGNEA	gut metagenome	technology	Illumina
SRS21535689	WGNEA	gut metagenome	platform	Illumina MiSeq
SRS21535689	WGNEA	gut metagenome	average_spotlength	601
SRS21535689	WGNEA	gut metagenome	library_layout	Paired
SRS21535689	WGNEA	gut metagenome	target_amplicon	V3-V4
SRS21535689	WGNEA	gut metagenome	submitters	Meriem FASSATOUI/Abdelmajid ABID/Rym KEFI
SRS21535689	WGNEA	gut metagenome	Isolate	DIURE
SRS21535689	WGNEA	gut metagenome	BioSampleModel	Metagenome or environmental
SRS21535690	OnUJI	gut metagenome	host	Homo sapiens
SRS21535690	OnUJI	gut metagenome	collection_date	2018
SRS21535690	OnUJI	gut metagenome	geo_loc_name	Tunisia
SRS21535690	OnUJI	gut metagenome	lat_lon	33.886917 N 9.537499 E
SRS21535690	OnUJI	gut metagenome	samp_size	110
SRS21535690	OnUJI	gut metagenome	participants_features	Adult
SRS21535690	OnUJI	gut metagenome	sex	male
SRS21535690	OnUJI	gut metagenome	age	71
SRS21535690	OnUJI	gut metagenome	study_design	Case-control
SRS21535690	OnUJI	gut metagenome	disease	Non Diabetic
SRS21535690	OnUJI	gut metagenome	study_title	Metataxonomic strategy to decipher gut
bacteriome biomarkers of type 2 diabetes and obesity in Tunisian population
SRS21535690	OnUJI	gut metagenome	continent	Africa
SRS21535690	OnUJI	gut metagenome	region	North Africa
SRS21535690	OnUJI	gut metagenome	assay_type	Amplicon
SRS21535690	OnUJI	gut metagenome	technology	Illumina
SRS21535690	OnUJI	gut metagenome	platform	Illumina MiSeq
SRS21535690	OnUJI	gut metagenome	average_spotlength	601
SRS21535690	OnUJI	gut metagenome	library_layout	Paired
SRS21535690	OnUJI	gut metagenome	target_amplicon	V3-V4
SRS21535690	OnUJI	gut metagenome	submitters	Meriem FASSATOUI/Abdelmajid ABID/Rym KEFI
SRS21535690	OnUJI	gut metagenome	Isolate	TUB
SRS21535690	OnUJI	gut metagenome	BioSampleModel	Metagenome or environmental
SRS21535691	KHXWAQ	gut metagenome	host	Homo sapiens
SRS21535691	KHXWAQ	gut metagenome	collection_date	2018
SRS21535691	KHXWAQ	gut metagenome	geo_loc_name	Tunisia
SRS21535691	KHXWAQ	gut metagenome	lat_lon	33.886917 N 9.537499 E
SRS21535691	KHXWAQ	gut metagenome	samp_size	110
SRS21535691	KHXWAQ	gut metagenome	participants_features	Adult
SRS21535691	KHXWAQ	gut metagenome	study_design	Case-control
SRS21535691	KHXWAQ	gut metagenome	disease	Non Diabetic
SRS21535691	KHXWAQ	gut metagenome	study_title	Metataxonomic strategy to decipher gut
bacteriome biomarkers of type 2 diabetes and obesity in Tunisian population
SRS21535691	KHXWAQ	gut metagenome	continent	Africa
```

SRS21535691 KHXWAQ gut metagenome region North Africa
SRS21535691 KHXWAQ gut metagenome assay_type Amplicon
SRS21535691 KHXWAQ gut metagenome technology Illumina
SRS21535691 KHXWAQ gut metagenome platform Illumina MiSeq
SRS21535691 KHXWAQ gut metagenome average_spotlength 601
SRS21535691 KHXWAQ gut metagenome library_layout Paired
SRS21535691 KHXWAQ gut metagenome target_amplicon V3-V4
SRS21535691 KHXWAQ gut metagenome submitters Meriem FASSATOUI/Abdelmajid ABID/Rym KEFI
SRS21535691 KHXWAQ gut metagenome Isolate BOVIS
SRS21535691 KHXWAQ gut metagenome BioSampleModel Metagenome or environmental
SRS21535692 THHYB gut metagenome host Homo sapiens
SRS21535692 THHYB gut metagenome collection_date 2018
SRS21535692 THHYB gut metagenome geo_loc_name Tunisia
SRS21535692 THHYB gut metagenome lat_lon 33.886917 N 9.537499 E
SRS21535692 THHYB gut metagenome samp_size 110
SRS21535692 THHYB gut metagenome participants_features Adult
SRS21535692 THHYB gut metagenome sex male
SRS21535692 THHYB gut metagenome age 61
SRS21535692 THHYB gut metagenome study_design Case-control
SRS21535692 THHYB gut metagenome disease Non Diabetic
SRS21535692 THHYB gut metagenome study_title Metataxonomic strategy to decipher gut bacteriome biomarkers of type 2 diabetes and obesity in Tunisian population
SRS21535692 THHYB gut metagenome continent Africa
SRS21535692 THHYB gut metagenome region North Africa
SRS21535692 THHYB gut metagenome assay_type Amplicon
SRS21535692 THHYB gut metagenome technology Illumina
SRS21535692 THHYB gut metagenome platform Illumina MiSeq
SRS21535692 THHYB gut metagenome average_spotlength 601
SRS21535692 THHYB gut metagenome library_layout Paired
SRS21535692 THHYB gut metagenome target_amplicon V3-V4
SRS21535692 THHYB gut metagenome submitters Meriem FASSATOUI/Abdelmajid ABID/Rym KEFI
SRS21535692 THHYB gut metagenome Isolate DEEPLEX
SRS21535692 THHYB gut metagenome BioSampleModel Metagenome or environmental
SRS21535693 UTAZA gut metagenome host Homo sapiens
SRS21535693 UTAZA gut metagenome collection_date 2018
SRS21535693 UTAZA gut metagenome geo_loc_name Tunisia
SRS21535693 UTAZA gut metagenome lat_lon 33.886917 N 9.537499 E
SRS21535693 UTAZA gut metagenome samp_size 110
SRS21535693 UTAZA gut metagenome participants_features Adult
SRS21535693 UTAZA gut metagenome sex female
SRS21535693 UTAZA gut metagenome age 30
SRS21535693 UTAZA gut metagenome study_design Case-control
SRS21535693 UTAZA gut metagenome disease Obese
SRS21535693 UTAZA gut metagenome disease_ontology_id 9970
SRS21535693 UTAZA gut metagenome study_title Metataxonomic strategy to decipher gut bacteriome biomarkers of type 2 diabetes and obesity in Tunisian population
SRS21535693 UTAZA gut metagenome continent Africa
SRS21535693 UTAZA gut metagenome region North Africa
SRS21535693 UTAZA gut metagenome assay_type Amplicon
SRS21535693 UTAZA gut metagenome technology Illumina
SRS21535693 UTAZA gut metagenome platform Illumina MiSeq
SRS21535693 UTAZA gut metagenome average_spotlength 601
SRS21535693 UTAZA gut metagenome library_layout Paired
SRS21535693 UTAZA gut metagenome target_amplicon V3-V4
SRS21535693 UTAZA gut metagenome submitters Meriem FASSATOUI/Abdelmajid ABID/Rym KEFI
SRS21535693 UTAZA gut metagenome Isolate GUTmicr
SRS21535693 UTAZA gut metagenome BioSampleModel Metagenome or environmental
SRS21535694 VSAL gut metagenome host Homo sapiens
SRS21535694 VSAL gut metagenome collection_date 2018
SRS21535694 VSAL gut metagenome geo_loc_name Tunisia
SRS21535694 VSAL gut metagenome lat_lon 33.886917 N 9.537499 E
SRS21535694 VSAL gut metagenome samp_size 110
SRS21535694 VSAL gut metagenome participants_features Adult
SRS21535694 VSAL gut metagenome sex male
SRS21535694 VSAL gut metagenome age 50
SRS21535694 VSAL gut metagenome study_design Case-control
SRS21535694 VSAL gut metagenome disease Obese
SRS21535694 VSAL gut metagenome disease_ontology_id 9970
SRS21535694 VSAL gut metagenome study_title Metataxonomic strategy to decipher gut

```
bacteriome biomarkers of type 2 diabetes and obesity in Tunisian population
SRS21535694     VSAL    gut metagenome  continent       Africa
SRS21535694     VSAL    gut metagenome  region  North Africa
SRS21535694     VSAL    gut metagenome  assay_type      Amplicon
SRS21535694     VSAL    gut metagenome  technology      Illumina
SRS21535694     VSAL    gut metagenome  platform        Illumina MiSeq
SRS21535694     VSAL    gut metagenome  average_spotlength      601
SRS21535694     VSAL    gut metagenome  library_layout  Paired
SRS21535694     VSAL    gut metagenome  target_amplicon V3-V4
SRS21535694     VSAL    gut metagenome  submitters      Meriem FASSATOUI/Abdelmajid ABID/Rym KEFI
SRS21535694     VSAL    gut metagenome  Isolate saliva
SRS21535694     VSAL    gut metagenome  BioSampleModel  Metagenome or environmental
SRS21535695     1UR     gut metagenome  host    Homo sapiens
SRS21535695     1UR     gut metagenome  collection_date 2018
SRS21535695     1UR     gut metagenome  geo_loc_name    Tunisia
SRS21535695     1UR     gut metagenome  lat_lon 33.886917 N 9.537499 E
SRS21535695     1UR     gut metagenome  samp_size       110
SRS21535695     1UR     gut metagenome  participants_features   Adult
SRS21535695     1UR     gut metagenome  sex     female
SRS21535695     1UR     gut metagenome  age     36
SRS21535695     1UR     gut metagenome  study_design    Case-control
SRS21535695     1UR     gut metagenome  disease Non Diabetic
SRS21535695     1UR     gut metagenome  study_title     Metataxonomic strategy to decipher gut
bacteriome biomarkers of type 2 diabetes and obesity in Tunisian population
SRS21535695     1UR     gut metagenome  continent       Africa
SRS21535695     1UR     gut metagenome  region  North Africa
SRS21535695     1UR     gut metagenome  assay_type      Amplicon
SRS21535695     1UR     gut metagenome  technology      Illumina
SRS21535695     1UR     gut metagenome  platform        Illumina MiSeq
SRS21535695     1UR     gut metagenome  average_spotlength      601
SRS21535695     1UR     gut metagenome  library_layout  Paired
SRS21535695     1UR     gut metagenome  target_amplicon V3-V4
SRS21535695     1UR     gut metagenome  submitters      Meriem FASSATOUI/Abdelmajid ABID/Rym KEFI
SRS21535695     1UR     gut metagenome  Isolate yl
SRS21535695     1UR     gut metagenome  BioSampleModel  Metagenome or environmental
SRS21535696     ETUI    gut metagenome  host    Homo sapiens
SRS21535696     ETUI    gut metagenome  collection_date 2018
SRS21535696     ETUI    gut metagenome  geo_loc_name    Tunisia
SRS21535696     ETUI    gut metagenome  lat_lon 33.886917 N 9.537499 E
SRS21535696     ETUI    gut metagenome  samp_size       110
SRS21535696     ETUI    gut metagenome  participants_features   Adult
SRS21535696     ETUI    gut metagenome  sex     female
SRS21535696     ETUI    gut metagenome  age     46
SRS21535696     ETUI    gut metagenome  study_design    Case-control
SRS21535696     ETUI    gut metagenome  disease Obese
SRS21535696     ETUI    gut metagenome  disease_ontology_id     9970
SRS21535696     ETUI    gut metagenome  study_title     Metataxonomic strategy to decipher gut
bacteriome biomarkers of type 2 diabetes and obesity in Tunisian population
SRS21535696     ETUI    gut metagenome  continent       Africa
SRS21535696     ETUI    gut metagenome  region  North Africa
SRS21535696     ETUI    gut metagenome  assay_type      Amplicon
SRS21535696     ETUI    gut metagenome  technology      Illumina
SRS21535696     ETUI    gut metagenome  platform        Illumina MiSeq
SRS21535696     ETUI    gut metagenome  average_spotlength      601
SRS21535696     ETUI    gut metagenome  library_layout  Paired
SRS21535696     ETUI    gut metagenome  target_amplicon V3-V4
SRS21535696     ETUI    gut metagenome  submitters      Meriem FASSATOUI/Abdelmajid ABID/Rym KEFI
SRS21535696     ETUI    gut metagenome  Isolate fec
SRS21535696     ETUI    gut metagenome  BioSampleModel  Metagenome or environmental
SRS21535697     GUMT    gut metagenome  host    Homo sapiens
SRS21535697     GUMT    gut metagenome  collection_date 2018
SRS21535697     GUMT    gut metagenome  geo_loc_name    Tunisia
SRS21535697     GUMT    gut metagenome  lat_lon 33.886917 N 9.537499 E
SRS21535697     GUMT    gut metagenome  samp_size       110
SRS21535697     GUMT    gut metagenome  participants_features   Adult
SRS21535697     GUMT    gut metagenome  sex     female
SRS21535697     GUMT    gut metagenome  age     43
SRS21535697     GUMT    gut metagenome  study_design    Case-control
SRS21535697     GUMT    gut metagenome  disease Obese
```

SRS21535697 GUMT gut metagenome disease_ontology_id 9970
SRS21535697 GUMT gut metagenome study_title Metataxonomic strategy to decipher gut bacteriome biomarkers of type 2 diabetes and obesity in Tunisian population
SRS21535697 GUMT gut metagenome continent Africa
SRS21535697 GUMT gut metagenome region North Africa
SRS21535697 GUMT gut metagenome assay_type Amplicon
SRS21535697 GUMT gut metagenome technology Illumina
SRS21535697 GUMT gut metagenome platform Illumina MiSeq
SRS21535697 GUMT gut metagenome average_spotlength 601
SRS21535697 GUMT gut metagenome library_layout Paired
SRS21535697 GUMT gut metagenome target_amplicon V3-V4
SRS21535697 GUMT gut metagenome submitters Meriem FASSATOUI/Abdelmajid ABID/Rym KEFI
SRS21535697 GUMT gut metagenome Isolate culture
SRS21535697 GUMT gut metagenome BioSampleModel Metagenome or environmental
SRS21535698 IOPDY gut metagenome host Homo sapiens
SRS21535698 IOPDY gut metagenome collection_date 2018
SRS21535698 IOPDY gut metagenome geo_loc_name Tunisia
SRS21535698 IOPDY gut metagenome lat_lon 33.886917 N 9.537499 E
SRS21535698 IOPDY gut metagenome samp_size 110
SRS21535698 IOPDY gut metagenome participants_features Adult
SRS21535698 IOPDY gut metagenome sex female
SRS21535698 IOPDY gut metagenome age 52
SRS21535698 IOPDY gut metagenome study_design Case-control
SRS21535698 IOPDY gut metagenome disease Obese
SRS21535698 IOPDY gut metagenome disease_ontology_id 9970
SRS21535698 IOPDY gut metagenome study_title Metataxonomic strategy to decipher gut bacteriome biomarkers of type 2 diabetes and obesity in Tunisian population
SRS21535698 IOPDY gut metagenome continent Africa
SRS21535698 IOPDY gut metagenome region North Africa
SRS21535698 IOPDY gut metagenome assay_type Amplicon
SRS21535698 IOPDY gut metagenome technology Illumina
SRS21535698 IOPDY gut metagenome platform Illumina MiSeq
SRS21535698 IOPDY gut metagenome average_spotlength 601
SRS21535698 IOPDY gut metagenome library_layout Paired
SRS21535698 IOPDY gut metagenome target_amplicon V3-V4
SRS21535698 IOPDY gut metagenome submitters Meriem FASSATOUI/Abdelmajid ABID/Rym KEFI
SRS21535698 IOPDY gut metagenome Isolate colony
SRS21535698 IOPDY gut metagenome BioSampleModel Metagenome or environmental
SRS21535699 PUNALU gut metagenome host Homo sapiens
SRS21535699 PUNALU gut metagenome collection_date 2018
SRS21535699 PUNALU gut metagenome geo_loc_name Tunisia
SRS21535699 PUNALU gut metagenome lat_lon 33.886917 N 9.537499 E
SRS21535699 PUNALU gut metagenome samp_size 110
SRS21535699 PUNALU gut metagenome participants_features Adult
SRS21535699 PUNALU gut metagenome sex female
SRS21535699 PUNALU gut metagenome age 58
SRS21535699 PUNALU gut metagenome study_design Case-control
SRS21535699 PUNALU gut metagenome disease Obese
SRS21535699 PUNALU gut metagenome disease_ontology_id 9970
SRS21535699 PUNALU gut metagenome study_title Metataxonomic strategy to decipher gut bacteriome biomarkers of type 2 diabetes and obesity in Tunisian population
SRS21535699 PUNALU gut metagenome continent Africa
SRS21535699 PUNALU gut metagenome region North Africa
SRS21535699 PUNALU gut metagenome assay_type Amplicon
SRS21535699 PUNALU gut metagenome technology Illumina
SRS21535699 PUNALU gut metagenome platform Illumina MiSeq
SRS21535699 PUNALU gut metagenome average_spotlength 601
SRS21535699 PUNALU gut metagenome library_layout Paired
SRS21535699 PUNALU gut metagenome target_amplicon V3-V4
SRS21535699 PUNALU gut metagenome submitters Meriem FASSATOUI/Abdelmajid ABID/Rym KEFI
SRS21535699 PUNALU gut metagenome Isolate hour
SRS21535699 PUNALU gut metagenome BioSampleModel Metagenome or environmental
SRS21535700 VOtSNE gut metagenome host Homo sapiens
SRS21535700 VOtSNE gut metagenome collection_date 2018
SRS21535700 VOtSNE gut metagenome geo_loc_name Tunisia
SRS21535700 VOtSNE gut metagenome lat_lon 33.886917 N 9.537499 E
SRS21535700 VOtSNE gut metagenome samp_size 110
SRS21535700 VOtSNE gut metagenome participants_features Adult
SRS21535700 VOtSNE gut metagenome sex male

```
SRS21535700	VOtSNE	gut metagenome	age	42
SRS21535700	VOtSNE	gut metagenome	study_design	Case-control
SRS21535700	VOtSNE	gut metagenome	disease	Obese
SRS21535700	VOtSNE	gut metagenome	disease_ontology_id	9970
SRS21535700	VOtSNE	gut metagenome	study_title	Metataxonomic strategy to decipher gut bacteriome biomarkers of type 2 diabetes and obesity in Tunisian population
SRS21535700	VOtSNE	gut metagenome	continent	Africa
SRS21535700	VOtSNE	gut metagenome	region	North Africa
SRS21535700	VOtSNE	gut metagenome	assay_type	Amplicon
SRS21535700	VOtSNE	gut metagenome	technology	Illumina
SRS21535700	VOtSNE	gut metagenome	platform	Illumina MiSeq
SRS21535700	VOtSNE	gut metagenome	average_spotlength	601
SRS21535700	VOtSNE	gut metagenome	library_layout	Paired
SRS21535700	VOtSNE	gut metagenome	target_amplicon	V3-V4
SRS21535700	VOtSNE	gut metagenome	submitters	Meriem FASSATOUI/Abdelmajid ABID/Rym KEFI
SRS21535700	VOtSNE	gut metagenome	Isolate	mont
SRS21535700	VOtSNE	gut metagenome	BioSampleModel	Metagenome or environmental
SRS21535701	UMEGO	gut metagenome	host	Homo sapiens
SRS21535701	UMEGO	gut metagenome	collection_date	2018
SRS21535701	UMEGO	gut metagenome	geo_loc_name	Tunisia
SRS21535701	UMEGO	gut metagenome	lat_lon	33.886917 N 9.537499 E
SRS21535701	UMEGO	gut metagenome	samp_size	110
SRS21535701	UMEGO	gut metagenome	participants_features	Adult
SRS21535701	UMEGO	gut metagenome	sex	female
SRS21535701	UMEGO	gut metagenome	age	61
SRS21535701	UMEGO	gut metagenome	study_design	Case-control
SRS21535701	UMEGO	gut metagenome	disease	Obese
SRS21535701	UMEGO	gut metagenome	disease_ontology_id	9970
SRS21535701	UMEGO	gut metagenome	study_title	Metataxonomic strategy to decipher gut bacteriome biomarkers of type 2 diabetes and obesity in Tunisian population
SRS21535701	UMEGO	gut metagenome	continent	Africa
SRS21535701	UMEGO	gut metagenome	region	North Africa
SRS21535701	UMEGO	gut metagenome	assay_type	Amplicon
SRS21535701	UMEGO	gut metagenome	technology	Illumina
SRS21535701	UMEGO	gut metagenome	platform	Illumina MiSeq
SRS21535701	UMEGO	gut metagenome	average_spotlength	601
SRS21535701	UMEGO	gut metagenome	library_layout	Paired
SRS21535701	UMEGO	gut metagenome	target_amplicon	V3-V4
SRS21535701	UMEGO	gut metagenome	submitters	Meriem FASSATOUI/Abdelmajid ABID/Rym KEFI
SRS21535701	UMEGO	gut metagenome	Isolate	sec
SRS21535701	UMEGO	gut metagenome	BioSampleModel	Metagenome or environmental
SRS21535702	TRISBO	gut metagenome	host	Homo sapiens
SRS21535702	TRISBO	gut metagenome	collection_date	2018
SRS21535702	TRISBO	gut metagenome	geo_loc_name	Tunisia
SRS21535702	TRISBO	gut metagenome	lat_lon	33.886917 N 9.537499 E
SRS21535702	TRISBO	gut metagenome	samp_size	110
SRS21535702	TRISBO	gut metagenome	participants_features	Adult
SRS21535702	TRISBO	gut metagenome	sex	male
SRS21535702	TRISBO	gut metagenome	age	55
SRS21535702	TRISBO	gut metagenome	study_design	Case-control
SRS21535702	TRISBO	gut metagenome	disease	Obese
SRS21535702	TRISBO	gut metagenome	disease_ontology_id	9970
SRS21535702	TRISBO	gut metagenome	study_title	Metataxonomic strategy to decipher gut bacteriome biomarkers of type 2 diabetes and obesity in Tunisian population
SRS21535702	TRISBO	gut metagenome	continent	Africa
SRS21535702	TRISBO	gut metagenome	region	North Africa
SRS21535702	TRISBO	gut metagenome	assay_type	Amplicon
SRS21535702	TRISBO	gut metagenome	technology	Illumina
SRS21535702	TRISBO	gut metagenome	platform	Illumina MiSeq
SRS21535702	TRISBO	gut metagenome	average_spotlength	601
SRS21535702	TRISBO	gut metagenome	library_layout	Paired
SRS21535702	TRISBO	gut metagenome	target_amplicon	V3-V4
SRS21535702	TRISBO	gut metagenome	submitters	Meriem FASSATOUI/Abdelmajid ABID/Rym KEFI
SRS21535702	TRISBO	gut metagenome	Isolate	braca
SRS21535702	TRISBO	gut metagenome	BioSampleModel	Metagenome or environmental
SRS21535703	JOMQ	gut metagenome	host	Homo sapiens
SRS21535703	JOMQ	gut metagenome	collection_date	2018
SRS21535703	JOMQ	gut metagenome	geo_loc_name	Tunisia
SRS21535703	JOMQ	gut metagenome	lat_lon	33.886917 N 9.537499 E
```

```
SRS21535703	JOMQ	gut metagenome	samp_size	110
SRS21535703	JOMQ	gut metagenome	participants_features	Adult
SRS21535703	JOMQ	gut metagenome	sex	male
SRS21535703	JOMQ	gut metagenome	age	49
SRS21535703	JOMQ	gut metagenome	study_design	Case-control
SRS21535703	JOMQ	gut metagenome	disease	Obese
SRS21535703	JOMQ	gut metagenome	disease_ontology_id	9970
SRS21535703	JOMQ	gut metagenome	study_title	Metataxonomic strategy to decipher gut
bacteriome biomarkers of type 2 diabetes and obesity in Tunisian population
SRS21535703	JOMQ	gut metagenome	continent	Africa
SRS21535703	JOMQ	gut metagenome	region	North Africa
SRS21535703	JOMQ	gut metagenome	assay_type	Amplicon
SRS21535703	JOMQ	gut metagenome	technology	Illumina
SRS21535703	JOMQ	gut metagenome	platform	Illumina MiSeq
SRS21535703	JOMQ	gut metagenome	average_spotlength	601
SRS21535703	JOMQ	gut metagenome	library_layout	Paired
SRS21535703	JOMQ	gut metagenome	target_amplicon	V3-V4
SRS21535703	JOMQ	gut metagenome	submitters	Meriem FASSATOUI/Abdelmajid ABID/Rym KEFI
SRS21535703	JOMQ	gut metagenome	Isolate	hadz
SRS21535703	JOMQ	gut metagenome	BioSampleModel	Metagenome or environmental
SRS21535704	YHWA	gut metagenome	host	Homo sapiens
SRS21535704	YHWA	gut metagenome	collection_date	2018
SRS21535704	YHWA	gut metagenome	geo_loc_name	Tunisia
SRS21535704	YHWA	gut metagenome	lat_lon	33.886917 N 9.537499 E
SRS21535704	YHWA	gut metagenome	samp_size	110
SRS21535704	YHWA	gut metagenome	participants_features	Adult
SRS21535704	YHWA	gut metagenome	sex	male
SRS21535704	YHWA	gut metagenome	age	59
SRS21535704	YHWA	gut metagenome	study_design	Case-control
SRS21535704	YHWA	gut metagenome	disease	Type 2 diabetic
SRS21535704	YHWA	gut metagenome	disease_ontology_id	9352
SRS21535704	YHWA	gut metagenome	study_title	Metataxonomic strategy to decipher gut
bacteriome biomarkers of type 2 diabetes and obesity in Tunisian population
SRS21535704	YHWA	gut metagenome	continent	Africa
SRS21535704	YHWA	gut metagenome	region	North Africa
SRS21535704	YHWA	gut metagenome	assay_type	Amplicon
SRS21535704	YHWA	gut metagenome	technology	Illumina
SRS21535704	YHWA	gut metagenome	platform	Illumina MiSeq
SRS21535704	YHWA	gut metagenome	average_spotlength	601
SRS21535704	YHWA	gut metagenome	library_layout	Paired
SRS21535704	YHWA	gut metagenome	target_amplicon	V3-V4
SRS21535704	YHWA	gut metagenome	submitters	Meriem FASSATOUI/Abdelmajid ABID/Rym KEFI
SRS21535704	YHWA	gut metagenome	Isolate	MASSA
SRS21535704	YHWA	gut metagenome	BioSampleModel	Metagenome or environmental
SRS21535705	PROPKJ	gut metagenome	host	Homo sapiens
SRS21535705	PROPKJ	gut metagenome	collection_date	2018
SRS21535705	PROPKJ	gut metagenome	geo_loc_name	Tunisia
SRS21535705	PROPKJ	gut metagenome	lat_lon	33.886917 N 9.537499 E
SRS21535705	PROPKJ	gut metagenome	samp_size	110
SRS21535705	PROPKJ	gut metagenome	participants_features	Adult
SRS21535705	PROPKJ	gut metagenome	sex	male
SRS21535705	PROPKJ	gut metagenome	age	66
SRS21535705	PROPKJ	gut metagenome	study_design	Case-control
SRS21535705	PROPKJ	gut metagenome	disease	Type 2 diabetic
SRS21535705	PROPKJ	gut metagenome	disease_ontology_id	9352
SRS21535705	PROPKJ	gut metagenome	study_title	Metataxonomic strategy to decipher gut
bacteriome biomarkers of type 2 diabetes and obesity in Tunisian population
SRS21535705	PROPKJ	gut metagenome	continent	Africa
SRS21535705	PROPKJ	gut metagenome	region	North Africa
SRS21535705	PROPKJ	gut metagenome	assay_type	Amplicon
SRS21535705	PROPKJ	gut metagenome	technology	Illumina
SRS21535705	PROPKJ	gut metagenome	platform	Illumina MiSeq
SRS21535705	PROPKJ	gut metagenome	average_spotlength	601
SRS21535705	PROPKJ	gut metagenome	library_layout	Paired
SRS21535705	PROPKJ	gut metagenome	target_amplicon	V3-V4
SRS21535705	PROPKJ	gut metagenome	submitters	Meriem FASSATOUI/Abdelmajid ABID/Rym KEFI
SRS21535705	PROPKJ	gut metagenome	Isolate	PYGMY
SRS21535705	PROPKJ	gut metagenome	BioSampleModel	Metagenome or environmental
SRS21535706	G89	gut metagenome	host	Homo sapiens
```

```
SRS21535706	G89	gut metagenome	collection_date	2018
SRS21535706	G89	gut metagenome	geo_loc_name	Tunisia
SRS21535706	G89	gut metagenome	lat_lon	33.886917 N 9.537499 E
SRS21535706	G89	gut metagenome	samp_size	110
SRS21535706	G89	gut metagenome	participants_features	Adult
SRS21535706	G89	gut metagenome	sex	male
SRS21535706	G89	gut metagenome	age	45
SRS21535706	G89	gut metagenome	study_design	Case-control
SRS21535706	G89	gut metagenome	disease	Non Diabetic
SRS21535706	G89	gut metagenome	study_title	Metataxonomic strategy to decipher gut
bacteriome biomarkers of type 2 diabetes and obesity in Tunisian population
SRS21535706	G89	gut metagenome	continent	Africa
SRS21535706	G89	gut metagenome	region	North Africa
SRS21535706	G89	gut metagenome	assay_type	Amplicon
SRS21535706	G89	gut metagenome	technology	Illumina
SRS21535706	G89	gut metagenome	platform	Illumina MiSeq
SRS21535706	G89	gut metagenome	average_spotlength	595
SRS21535706	G89	gut metagenome	library_layout	Paired
SRS21535706	G89	gut metagenome	target_amplicon	V3-V4
SRS21535706	G89	gut metagenome	submitters	Meriem FASSATOUI/Abdelmajid ABID/Rym KEFI
SRS21535706	G89	gut metagenome	Isolate	cla
SRS21535706	G89	gut metagenome	BioSampleModel	Metagenome or environmental
SRS21535707	CLOWIZ	gut metagenome	host	Homo sapiens
SRS21535707	CLOWIZ	gut metagenome	collection_date	2018
SRS21535707	CLOWIZ	gut metagenome	geo_loc_name	Tunisia
SRS21535707	CLOWIZ	gut metagenome	lat_lon	33.886917 N 9.537499 E
SRS21535707	CLOWIZ	gut metagenome	samp_size	110
SRS21535707	CLOWIZ	gut metagenome	participants_features	Adult
SRS21535707	CLOWIZ	gut metagenome	sex	male
SRS21535707	CLOWIZ	gut metagenome	age	66
SRS21535707	CLOWIZ	gut metagenome	study_design	Case-control
SRS21535707	CLOWIZ	gut metagenome	disease	Type 2 diabetic
SRS21535707	CLOWIZ	gut metagenome	disease_ontology_id	9352
SRS21535707	CLOWIZ	gut metagenome	study_title	Metataxonomic strategy to decipher gut
bacteriome biomarkers of type 2 diabetes and obesity in Tunisian population
SRS21535707	CLOWIZ	gut metagenome	continent	Africa
SRS21535707	CLOWIZ	gut metagenome	region	North Africa
SRS21535707	CLOWIZ	gut metagenome	assay_type	Amplicon
SRS21535707	CLOWIZ	gut metagenome	technology	Illumina
SRS21535707	CLOWIZ	gut metagenome	platform	Illumina MiSeq
SRS21535707	CLOWIZ	gut metagenome	average_spotlength	601
SRS21535707	CLOWIZ	gut metagenome	library_layout	Paired
SRS21535707	CLOWIZ	gut metagenome	target_amplicon	V3-V4
SRS21535707	CLOWIZ	gut metagenome	submitters	Meriem FASSATOUI/Abdelmajid ABID/Rym KEFI
SRS21535707	CLOWIZ	gut metagenome	Isolate	BEJA
SRS21535707	CLOWIZ	gut metagenome	BioSampleModel	Metagenome or environmental
SRS21535708	JNCRO	gut metagenome	host	Homo sapiens
SRS21535708	JNCRO	gut metagenome	collection_date	2018
SRS21535708	JNCRO	gut metagenome	geo_loc_name	Tunisia
SRS21535708	JNCRO	gut metagenome	lat_lon	33.886917 N 9.537499 E
SRS21535708	JNCRO	gut metagenome	samp_size	110
SRS21535708	JNCRO	gut metagenome	participants_features	Adult
SRS21535708	JNCRO	gut metagenome	sex	male
SRS21535708	JNCRO	gut metagenome	age	58
SRS21535708	JNCRO	gut metagenome	study_design	Case-control
SRS21535708	JNCRO	gut metagenome	disease	Type 2 diabetic
SRS21535708	JNCRO	gut metagenome	disease_ontology_id	9352
SRS21535708	JNCRO	gut metagenome	study_title	Metataxonomic strategy to decipher gut
bacteriome biomarkers of type 2 diabetes and obesity in Tunisian population
SRS21535708	JNCRO	gut metagenome	continent	Africa
SRS21535708	JNCRO	gut metagenome	region	North Africa
SRS21535708	JNCRO	gut metagenome	assay_type	Amplicon
SRS21535708	JNCRO	gut metagenome	technology	Illumina
SRS21535708	JNCRO	gut metagenome	platform	Illumina MiSeq
SRS21535708	JNCRO	gut metagenome	average_spotlength	601
SRS21535708	JNCRO	gut metagenome	library_layout	Paired
SRS21535708	JNCRO	gut metagenome	target_amplicon	V3-V4
SRS21535708	JNCRO	gut metagenome	submitters	Meriem FASSATOUI/Abdelmajid ABID/Rym KEFI
SRS21535708	JNCRO	gut metagenome	Isolate	ALUR
```

SRS21535708 JNCRO gut metagenome BioSampleModel Metagenome or environmental
SRS21535709 GYMMOD gut metagenome host Homo sapiens
SRS21535709 GYMMOD gut metagenome collection_date 2018
SRS21535709 GYMMOD gut metagenome geo_loc_name Tunisia
SRS21535709 GYMMOD gut metagenome lat_lon 33.886917 N 9.537499 E
SRS21535709 GYMMOD gut metagenome samp_size 110
SRS21535709 GYMMOD gut metagenome participants_features Adult
SRS21535709 GYMMOD gut metagenome sex male
SRS21535709 GYMMOD gut metagenome age 57
SRS21535709 GYMMOD gut metagenome study_design Case-control
SRS21535709 GYMMOD gut metagenome disease Type 2 diabetic
SRS21535709 GYMMOD gut metagenome disease_ontology_id 9352
SRS21535709 GYMMOD gut metagenome study_title Metataxonomic strategy to decipher gut bacteriome biomarkers of type 2 diabetes and obesity in Tunisian population
SRS21535709 GYMMOD gut metagenome continent Africa
SRS21535709 GYMMOD gut metagenome region North Africa
SRS21535709 GYMMOD gut metagenome assay_type Amplicon
SRS21535709 GYMMOD gut metagenome technology Illumina
SRS21535709 GYMMOD gut metagenome platform Illumina MiSeq
SRS21535709 GYMMOD gut metagenome average_spotlength 601
SRS21535709 GYMMOD gut metagenome library_layout Paired
SRS21535709 GYMMOD gut metagenome target_amplicon V3-V4
SRS21535709 GYMMOD gut metagenome submitters Meriem FASSATOUI/Abdelmajid ABID/Rym KEFI
SRS21535709 GYMMOD gut metagenome Isolate MOMB
SRS21535709 GYMMOD gut metagenome BioSampleModel Metagenome or environmental
SRS21535710 nFAST gut metagenome host Homo sapiens
SRS21535710 nFAST gut metagenome collection_date 2018
SRS21535710 nFAST gut metagenome geo_loc_name Tunisia
SRS21535710 nFAST gut metagenome lat_lon 33.886917 N 9.537499 E
SRS21535710 nFAST gut metagenome samp_size 110
SRS21535710 nFAST gut metagenome participants_features Adult
SRS21535710 nFAST gut metagenome sex female
SRS21535710 nFAST gut metagenome age 63
SRS21535710 nFAST gut metagenome study_design Case-control
SRS21535710 nFAST gut metagenome disease Type 2 diabetic
SRS21535710 nFAST gut metagenome disease_ontology_id 9352
SRS21535710 nFAST gut metagenome study_title Metataxonomic strategy to decipher gut bacteriome biomarkers of type 2 diabetes and obesity in Tunisian population
SRS21535710 nFAST gut metagenome continent Africa
SRS21535710 nFAST gut metagenome region North Africa
SRS21535710 nFAST gut metagenome assay_type Amplicon
SRS21535710 nFAST gut metagenome technology Illumina
SRS21535710 nFAST gut metagenome platform Illumina MiSeq
SRS21535710 nFAST gut metagenome average_spotlength 601
SRS21535710 nFAST gut metagenome library_layout Paired
SRS21535710 nFAST gut metagenome target_amplicon V3-V4
SRS21535710 nFAST gut metagenome submitters Meriem FASSATOUI/Abdelmajid ABID/Rym KEFI
SRS21535710 nFAST gut metagenome Isolate COPT
SRS21535710 nFAST gut metagenome BioSampleModel Metagenome or environmental
SRS21535711 BIVIR gut metagenome host Homo sapiens
SRS21535711 BIVIR gut metagenome collection_date 2018
SRS21535711 BIVIR gut metagenome geo_loc_name Tunisia
SRS21535711 BIVIR gut metagenome lat_lon 33.886917 N 9.537499 E
SRS21535711 BIVIR gut metagenome samp_size 110
SRS21535711 BIVIR gut metagenome participants_features Adult
SRS21535711 BIVIR gut metagenome sex male
SRS21535711 BIVIR gut metagenome age 46
SRS21535711 BIVIR gut metagenome study_design Case-control
SRS21535711 BIVIR gut metagenome disease Type 2 diabetic
SRS21535711 BIVIR gut metagenome disease_ontology_id 9352
SRS21535711 BIVIR gut metagenome study_title Metataxonomic strategy to decipher gut bacteriome biomarkers of type 2 diabetes and obesity in Tunisian population
SRS21535711 BIVIR gut metagenome continent Africa
SRS21535711 BIVIR gut metagenome region North Africa
SRS21535711 BIVIR gut metagenome assay_type Amplicon
SRS21535711 BIVIR gut metagenome technology Illumina
SRS21535711 BIVIR gut metagenome platform Illumina MiSeq
SRS21535711 BIVIR gut metagenome average_spotlength 601
SRS21535711 BIVIR gut metagenome library_layout Paired

```
SRS21535711	BIVIR	gut metagenome	target_amplicon V3-V4
SRS21535711	BIVIR	gut metagenome	submitters	Meriem FASSATOUI/Abdelmajid ABID/Rym KEFI
SRS21535711	BIVIR	gut metagenome	Isolate CREDIT
SRS21535711	BIVIR	gut metagenome	BioSampleModel	Metagenome or environmental
SRS21535712	YGRAM	gut metagenome	host	Homo sapiens
SRS21535712	YGRAM	gut metagenome	collection_date 2018
```
